# Supplementary material for: A near-continuous archaeological record of Pleistocene human occupation at Leang Bulu Bettue, Sulawesi, Indonesia
Source: PLoS One. 2025 Dec 23;20(12):e0337993. doi: 10.1371/journal.pone.0337993 (PMC12725638; doi:10.1371/journal.pone.0337993)
Supplement: S1 File — (DOCX) [file pone.0337993.s017.docx]

**SUPPORTING INFORMATION**

**SEDIMENT ANALYSIS**

**Sample Collection**

Sediment samples were collected from LBB in 2013 from square A1 in the Cave Mouth Trench and in 2017 from squares -C2 and -H2 in the Shelter Trench. A single sediment sample from each layer was collected from the Cave Mouth Trench to a total depth of 6.6 m. Sediment samples from the Shelter Trench were taken in vertical columns in 2 cm increments from the west wall of the southwest corner of square -C2 and the west wall in the centre of square -H2. The total depth of the column in square -C2 was 232 cm and the total depth of the column in square -H2 was 208 cm. Approximately 100 g of sediment was taken for each sample.

**Grain size analysis**

Sediment grain size for samples from the Cave Mouth Trench was measured using a Malvern Mastersizer 2000 at the University of Wollongong. Sediment grain size for samples from the Shelter Trench was measured at Flinders University via an Endecotts laboratory test sieve pack comprising 1 mm, 0.5 mm, 0.25 mm, 0.125 mm and 0.063 mm aperture sieves. These were vibrated for 5 minutes on an Endecotts Minor vibration device. The sieve fractions used correspond to the very coarse sand, coarse sand, medium sand, fine sand, very fine sand and clay/silt grain sizes in the Wentworth scale [1].

The results were converted into percentage weights of the total mass analysed and plotted in a column using a custom R script, shown in S1-3 Figs. The distribution of grain sizes for each layer was calculated using the Gradistat program [2].

**Magnetic susceptibility analysis**

Magnetic susceptibility analysis of the samples from the Shelter Trench was undertaken both on the excavation wall prior to sample collection and on unconsolidated sediment samples in the laboratory at Flinders University. Field measurements were undertaken using a Bartington MS3 with a MSF single frequency sensor. Low and high frequency Magnetic Susceptibility (MS) measurements were taken in the laboratory using a Bartington MS3 with a MS2B dual frequency sensor. Samples of ~20 gm were measured for 5 seconds each, including a blank measurement before and after each sample and corrected for mass. These measurements were repeated three times and then averaged to determine χ and χfd% for each sample from both square -C2 and square -H2. The mass corrected high frequency (HF) and low frequency (LF) χ as well as χfd% were plotted compared to depth using a custom R script, as shown in S1 and S2 Figs.

**Geochemical analysis**

ICP-AES analysis on 24 samples from the Shelter Trench was undertaken by ALS Metallurgy in Spain. These samples were collected from the stratigraphic column in square -C2 in depths increments of 10 cm. They were finely crushed so that 70% had a grain size of less than 2 mm before being pulverised in an agate mill, after which they were dissolved in an Aqua Regia solution before being analysed with an ICP-AES to determine the concentration of the following elements: Ag, Al, As, Ba, Be, Bi, Ca, Cd, Co, Cu, Cr, Cu, Fe, Ga, K, La, Mg, Mn, Mo, Na, Ni, P, S, Sb, Sc, Sr, Th, Ti, Tl, U, V, W and Zn. The results were plotted as ppm against depth using a custom R script, as shown in S1-2 Figs.

XRD analysis on three sediment samples from the Shelter Trench was undertaken by ALS Metallurgy in Spain. These three samples were taken from the stratigraphic column in square -C2 from depths of ~62-66 cm (layer 3), 110-114 cm (layer 4a) and 182-186 cm (layer 5). The samples were pressed into a back-packed sample holder before using a Panalytical Empyrean XRD for analysis.

XRD analysis was undertaken at the University of Wollongong on all sediment samples from square A1 in the Cave Mouth Trench. Bulk samples were homogenised in a Tema crusher and XRD analysis was conducted at the same institution using a Philips X-ray diffractometer and Siroquant software (version 4), applying the Rietveld-based approach.

FTIR analysis was undertaken on all sediment samples from square -C2 in the Shelter Trench at the University of Cambridge using a Thermo Nicolet iS5 Ft-IR Spectrometer equipped with an iD1 Transmission Accessory using the KBR method. Approximately 1 g of sediment was ground with an agate mortar and pestle before being mixed with KBR and pressed into a die with 1.8 tons of pressure. The results were compared to the Weizmann Institute FTIR database to determine the mineralogy of each sample.

**Isotope geochemistry**

Strontium, neodymium and lead isotope analysis was undertaken on the <2 micron size fraction of sediment samples collected at depths of 10-12 cm (layer 2), 100-102 cm (layer 4a) and 200-202 cm (layer 5) from square -C2 in the Shelter Trench. These sub-samples were extracted using gravity separation and digested using HF, HNO3 and HCL. Potential interferences were removed with ion exchange chromatography prior to measuring using a Phoenix TIMS instrument at the University of Adelaide.

Results

**Grain size**

The grain size distribution from both sections is characterised by dramatic alternations between thin layers with large percentages (up to 35%) of pebble/cobble/boulder grains (which are fragments of limestone) and those with larger percentages of sand and clay/silt. The clay/silt percentage reaches more than 25% in parts of the sections and is, overall, much higher in section -C2 than -H2. The total percentage of sand sized grains is generally high, although these (as shown by the XRD, ICP-AES and FTIR) are largely limestone fragments and/or undispersed clays rather than quartz grains.

**Magnetic Susceptibility**

Mass corrected χ values cover a large range of 4.59 x 10^-7^ to 1.89 x 10^-5^ (section C2, low frequency), 4.4 x 10^-7^ to 1.88 x 10^-5^ (section C2, high frequency), 4.59 x 10.-7 to 1.89 x 10-5 χfd% values cover a similar, modest, range in both sections of 0.56% to 3.99% (section -C2) and 0.53% to 3.99% (section -H2).

**Geochemistry**

The ICP-AES analysis reveals that the major chemical components of the sediments in section C2 are Ca (1.24%-32.7% of each sample), Al (1.86%-12.5% of the sample) and Fe (0.67%-15.65% of the samples). The relative percentage of Ca and Al/Fe vary antithetically to one another, with Ca being high and Al/Fe being low in carbonate dominated layers and vice-versa in clay dominated layers.

The XRD results (S1 Table) show that samples from layer 3 and layer 5 are dominated by calcite (60% and 69%, respectively) and have smaller contributions of K-feldspar (19%, 6%), Illite (5% and 75), Kaolinite (3% and 4%) and “Clay Mineral” (mainly Smectite) (7% and 4%). The sample from layer 4a is dominated by K-feldspar (26%), “Clay Mineral” (17%), Illite (13%), Boemite (7%) and Goethite (6%).

The FTIR results show peaks dominated by calcite, carbonate and clays through the section.

**Isotope Geochemistry**

The Sr^87^/Sr^86^ and Nd^143^/Nd^144^ isotope results values are from a limited range of 708783 to .708464 and .512642 to .512651 (S2 Table). Lead isotope results are also from a very small range for all systems.

**SITE DESCRIPTION, EXCAVATION METHODS AND FINDS ANALYSIS**

The Maros-Pangkep karst system harbours a vast array of caves, cliff-foot shelters, dolines, niches and recesses [3]. Most habitable sites contain indications of prehistoric occupation, and hence this area has long been of interest to archaeologists [4-23]. The karsts are accessible from the western coast by following the river systems that drain the intervening coastal plain. The latter is likely to have been covered by lowland rainforest during warm and humid interglacial periods and characterised by more open vegetation (including tropical grasslands) during glacial phases [24, 25].

LBB is located at 4°59'31.18" S latitude and 119°40'5.53" E at an elevation of 18 m above sea level (Fig 1). It is approximately 30 km northeast of Makassar and around 20 km inland from the western shoreline of Sulawesi’s southern arm. Concerning the latter, at the height of the LGM a 60-km-wide shelf area adjacent to the present-day coast (the Spermonde shelf, now a shallow coral reef system) was exposed as dry land [26] (Fig 1). LBB was ~80 km inland at that time. Situated in the Tompobalang area at the foot of a karst tower, LBB is one of many archaeological sites in the Leang-Leang valley. It is around 1.5 km from the cliff-foot rock-shelter Leang Burung 2 [13], 1 km from Leang Timpuseng with its rock art dated to at least 40 ka [27], and 300 m from Ulu Leang 1 cave [10]. The latter is the flagship site of the mid- to late-Holocene foraging culture (~8 to 1.5 kyr cal BP) known as the Toalean, which is only found in a ~10,000 km^2^ area of South Sulawesi [5, 28].

*Cave Mouth Trench*

Initial fieldwork at LBB in 2013 involved the excavation of a 2 x 1 m test-pit (squares A1-A2) into undisturbed floor deposits in the front part of the cave, just inside the mouth and beneath a part of the ceiling containing rock art (faded hand stencils overlaid by Austronesian art) and a projecting ledge with active stalactites. The test-pit was benched at about 1.6 m below datum (BD) and a smaller (1 x 1 m) test-pit was then excavated to a depth of 6.5 m BD. In 2014, squares A1 and A2 were extended one metre to the north (squares B1/B2) and south (squares -A1/-A2), and the combined 2 x 3 m trench was excavated to a depth of ~4 m BD. In 2015, we extended this trench vertically to around 7 m BD, at which point we excavated a one metre square test-pit (square -A2) to a depth of 8 m BD. Work was abandoned on the Cave Mouth Trench due to groundwater inundation in the deepest square.

*Shelter Trench*

During the 2015 season we also undertook excavations in the rock-shelter area immediately outside and to the south of the cave mouth. Initially, we opened up a 1 x 1 m test-pit in the central floor area of the shelter (square -H2), the northern edge of which was located 6 m to the south of square -A2. The objective of this test-pit was to determine whether the upper cave stratigraphy revealed by the Cave Mouth Trench (layers 3 and 4a-b) continued into the rock-shelter area. After the test-pit in square -H2 confirmed that the cave stratigraphy did extend into the shelter we expanded the scale of the latter excavations. Work in -H2 continued, while we commenced excavation of a 1 m-wide by 5 m-long trench (squares -G2, F2, -E2, -D2 and -C2) running north from square -H2 to just inside the cave mouth. We left a 1 m-wide baulk between the northern edge of the shelter trench (-C2) and the southern edge of the Cave Mouth Trench (-A2). Excavation continued in 2017, widening the 2015 squares in the Shelter Trench to the south and east. We also excavated a test-pit (square -Z2) in the southernmost part of the shelter (results are reported in [29 and 30]).

In 2018 the excavation continued in the Shelter Trench. The focus was on expanding upon the work in viable squares. However, excavation was disrupted in some areas or could not be continued at all due to practical constraints. While the deposits exposed in the Cave Mouth Trench were largely free of large rocks and other such obstructions in the stratified deposit, excavations in the Shelter Trench revealed masses of limestone roof-fall blocks and collapsed stalactites that presented serious practical difficulties. The largest of these blocks, enormous sections of the overhead cliff-face, were located in squares -D2, -E2 and -F2, effectively forcing us to discontinue our excavations in these squares at depths of between 1.5 to 2 m below datum. Excavations in square -H2 also revealed a number of somewhat smaller, but still troublesome, limestone blocks at depths of around 3 m below datum. This concentrated mass of fallen debris has impeded our efforts to correlate the deepest strata exposed in the Shelter Trench with those of the Cave Mouth Trench to the north (see below).

Work in the Shelter Trench resumed in 2019, during which a total of 20 squares was excavated, including existing squares and four new squares (-J2, -J1, J1/1, and -J1/2), reaching a maximum depth of 540 cm below datum in square -C2. Extension of the squares to the south was necessary due to a crack forming in the southern trench wall at the end of the 2018 excavation season. Excavation continued in the central part of the main units, particularly in the eastern section. Excavation on the western side of the main trench was limited to a few squares (-H2, -G1, -H1, -I1) due once more to the presence of boulders.

Fieldwork recommenced in 2023 after a hiatus in field research related to the impacts of the COVID-19 pandemic. That work involved continuing excavations in the existing Shelter Trench and opening new squares in the J row (-J2, -J1, J1/1, and -J1/2). A total of 12 squares was excavated, with the deepest square reaching a depth of 8 m BD.

**TREATMENT AND ANALYSIS OF FINDS**

Finds in some strata (especially layers 4a-f) were so numerous, and the bones (and in some cases, chert artefacts [30]) so fragile, that it was necessary to excavate much of these portions of the deposit using small bamboo spatulas and probing tools, rather than with trowels. Certain archaeological finds received special treatment according to their type and condition. For example, fragile bones were stabilized with a mixture of B-76 Paraloid resin and acetone and in some instances removed along with a portion of the surrounding sediment (i.e., lifting *en bloc*). This was also done in some cases where delicate finds were concentrated *en masse*.

Before removing them from their original context, all identified archaeological finds were 3D-plotted with a Total Station (Trimble M3 and Trimble S7 Robotic). In some instances, Total Station recording combined with photogrammetry was applied to non-artefactual objects, such as limestone blocks. The photogrammetry method employed involved capturing images of the object from a close distance (Close Range Photogrammetry) using a convergent photo-taking technique, where multiple photographs are taken around the object with a digital camera. This approach ensures an accurate representation of the real shape of the photographed object. The resulting data was then processed using Agisoft Metashape Professional software versions 1.x (64 bit) - 2.x (64 bit). Before exporting to a .dae file, registration of the object was conducted using points that were previously measured (Object Control Points) with a Total Station. The results were integrated with the plotted artefacts and other findings, enabling a realistic reconstruction of the excavation squares based on available data. Excavated sediment was labelled according to spit and layer, weighed, and then wet-sieved through 3 mm and 1 mm meshes. The sieved residues were dried, bagged according to their labels, and sorted into categories (lithic, faunal, non-artefactual materials, and so on).

All plotted finds, loose finds, block finds, and sieved sediment residues were transported to our basecamp in the nearby Leang-Leang Archaeological Park for further treatment. Initial handling of the finds involved various processes including washing, conservation, photographing, photogrammetry, 3D laser scanning, and temporary storage. Washing was performed only on finds that were deemed non-fragile, using water and soft-bristled brushes. Fragile finds were handled by the conservation team. The conservation process included cleaning, consolidation, and reconstruction. Some finds were encased in calcite deposits from limestone weathering mixed with sediment, requiring the use of a weak solution of acetic acid to remove the calcite without damaging the finds. To nullify the potentially damaging effects of the acetic acid, finds so treated were afterwards immersed in distilled water for at least twice as long as they had been in the acetic acid solution. They were then dried very slowly. Particularly delicate finds, once dry, were consolidated with a mixture of acetone and B-76 Paraloid resin. After conservation, finds were stored at the BRIN office in Makassar.

Analyses of the artefact and faunal assemblages recovered during excavations at LBB are still ongoing and the laborious process of sorting through the large stockpile of wet-sieved residues (3-mm and 1-mm) is underway. Considerable numbers of faunal remains and stone artefacts are likely contained within cemented masses of ashy deposit removed in bulk from certain stratigraphic layers within the Shelter Trench (e.g., layer 4c, square -H2). To build a general picture of the archaeological sequence at the site, we have undertaken priority analyses of 3D-plotted finds from selected excavation squares that span key areas of the stratigraphic record, in particular the deep deposits. These results are reported here.

For the present study we have analysed all excavated faunal remains from square -A1 down to a total depth of 6.1 m (6.2 m BD), as well as all faunal remains from square -A2 from below 4.9 m depth (5.06 m BD) and down to the deepest level at a depth BD of 8 m (layer 13). In addition, special finds such as complete megafauna elements, but originating from other squares, were also examined, measured, and, in some cases, photographed. Each bone item was described in terms of skeletal element, and attributed to the highest taxonomic level, representing either class (e.g., reptilian versus mammalia), order (Proboscidea), family or subfamily (Bovini), genus, and, in some cases, species. In the case of determinable and diagnostic elements, standard measurements were taken. In addition, various taphonomic characteristics, such as signs of burning, bone breakage patterns (fresh versus dry bone breakage), presence of cut marks or other indications of butchery, rounding caused by water transport, and polish as a potential sign of use-wear, were identified and recorded following standards in the literature (e.g., 31-35]).

Although a considerable proportion of the bones consists of small vertebrates, especially those originating from the upper stratigraphy (i.e., layers 4a-b, 4a-f), the main analytical focus was on megafauna remains. For instance, no attempt has yet been made to assign rodent to genera or species, but merely a distinction on family level was made (e.g., Muridae versus Sciuridae). However, in future the small faunal remains certainly warrant a more detailed study as these can yield important insight into paleoenvironmental factors. In addition, rare bird bone fragments have not been analysed other than being assigned to the Aves class. Most attention was focused on the megafauna remains, including the following groups: Proboscidea (including the genera *Palaeoloxodon* and *Stegodon*), Bovidae (cattle, and various *Bubalus* [*Anoa*] species), Suidae (including the genera *Sus*, *Babyrousa* and *Celebochoerus*), and a number of smaller vertebrate groups, such as the endemic Phalangeridae (*Ailurops ursinus*, and *Strigocuscus celebensis*), Primates (including the genera *Macaca* and *Tarsius*), and Viverridae. Cervidae and cattle were only found present in the upper two spits (layer 1). These animals represent taxa that were introduced recently to Sulawesi by modern humans.

Of long bone fragments, the cortical bone thickness (CBT) was recorded in order to obtain basic information regarding body size, even when the element or taxon could not be determined. Based on the CBT the fragments were then assigned a body size class based on comparison with other taxa of which the identity was known. The size classes distinguished are: 1) very small to small vertebrates (shrew to rat size); 2) small- to medium-sized vertebrates (e.g., primates, Phalangeridae, viverrids); 3) medium-sized vertebrates (suidae size); 4) medium- to large-sized (anoa size); 5) large-sized (cattle); and 6) very large-sized (Proboscideans). The resulting size data should be considered as tentative. They have been used to obtain a basic assessment of the percentage of large versus small vertebrates, grouping all size classes below cuscus size (including cuscus-sized vertebrates) together and lumping all size classes above cuscus size in the informal “large” vertebrate group. This conflation was done to investigate the relative preponderance of “small” vertebrates versus megafauna and changes therein throughout the sequence.

In the deeper fluvial layers (layer 10a-e and layer 13), a noteworthy proportion of the finds consists of heavily fossilized fish teeth, including from large extinct sharks, as well as crustacean fragments and other unidentifiable remains of marine fauna obtained from wet-sieving. These elements are obviously reworked and most likely originate from the bedrock limestone of the Tonasa Formation. However, a few fish vertebra and other skeletal fish parts, that are clearly much less heavily fossilized, do occur in the Phase II deposits. These appear not to be reworked. Some heavily fossilized rib fragments of Sirenia (dugong family) in layer 10 are characterized by a very dense bone structure and heavy rounding. These are also probably reworked from the local limestone bedrock, like the majority of the fish teeth.

To date, we have completed technological analyses of all LBB stone artefacts measuring >3 mm in maximum dimension recovered from square -H1 (*n* = 4328, [36]), as well as a large sample of artefacts recovered from the other 34 squares of the main excavation. In total, 25,788 individual lithic artefacts have been analysed, a sample that makes up approximately 53% of the estimated 48,468 artefacts recovered from the site up until now (see [30]). The primary analytical goal was to generate models of the reduction sequences that produced the assemblage for comparison with other regional lithic collections, as part of author YLP’s PhD research project [36]. Analysis involved classifying all stone artefacts measuring over 3 mm, including broken artefacts, into a technological typology, recording artefact attributes, and collecting standard metric data to facilitate empirical comparisons. Technological classifications and technical terms follow protocols published elsewhere [37]. Limited inspection for residues (ochre and silica gloss) was conducted under low-power magnification (hand-held Dino-Lite digital microscope with up to up to 220x magnification, combined with hand lenses of 10x and 20x magnification). Further analysis using high-power techniques would likely reveal other examples.

**STRATIGRAPHIC OBSERVATIONS (PHASE II)**

In colour and composition, layer 4a inside the rock-shelter looks much like it does inside the cave mouth (i.e., dark brown clayey texture), especially in the squares closest to the southern wall of the main cave excavation (squares -C2 and -D2). The mean grain size of this unit in square -C2 is fine sand and in -H2 is medium sand and it is poorly to very poorly sorted. The XRD results show that this layer is dominated by K-feldspar and “Clay Mineral”. However, the sedimentary matrix becomes noticeably drier, and slightly less compact, towards the central floor area of the rock-shelter. This change in the sedimentary layer from the cave mouth to the shelter area is exemplified by the condition of this deposit in the southernmost square (-H2), where it is distinctly less consolidated and moist, and consequently the sedimentary matrix is more friable. Bone and shell preservation is generally also better in the Shelter Trench compared with the Cave Mouth Trench. For instance, in square -H2 (Shelter Trench), wet-sieving yielded large numbers of near-complete rodent mandibles as well as abundant complete rat long bones, faunal elements that were not recovered from layer 4a in the Cave Mouth Trench. The marked increase in the quantity of loose, complete shells within layer 4a in the shelter also suggests better survival of organics in drier portions of the deposit. Practically all the shells are of the freshwater gastropod Tylomelania (formerly known as Brotia perfecta [38]), most of which appear to have been burnt. No marine shell species were recovered.

Fragments of brecciated archaeological sediment were also found within layer 4a, enveloping various finds, especially bones and stone artefacts. Brecciation is likely to have occurred in parts of the cave floor owing to dripwater from overhead speleothem formations (e.g., stalactites), a process that continues at the site today. Concentrations of brecciated sediment were commonly found at the base of the layer, bordering underlying layer 4b.

It is important to note that the upper portion of the Phase II strata (layer 4a) was affected by an episode of erosion and mass loss of deposits, probably due to hydrological activity, and a subsequent period of re-infilling. The presence of remnant layers of flowstone on the rear wall of the rock-shelter suggests that the very top of layer 4a in this area was originally sealed or capped by a series of thick, flat-lying geogenic strata. This flowstone unit was then eroded away *en masse*, followed by a further erosional event that removed close to a meter or so of the upper part of layer 4a, at least in the shelter, leaving the top portion of the sequence preserved only in the embankment against the east wall, outcropping at the surface.

This erosional event and subsequent mass loss of deposits seems to have taken place around 20 ka during the LGM (Fig 14). This dating is inferred from two lines of evidence. First, a small stalagmite formed on the uppermost surface of the deep erosional cut in layer 4a, with the initial stages of speleothem growth taking place between ~20 ka and 16 ka [39]. Second, a ^14^C date of 21.1–20.6 kyr cal BP (Wk-46529) was obtained on a *T. perfecta* shell extracted from the remnant portion of uppermost layer 4a exposed on the shelter wall below the above-mentioned flowstone. After the erosional event around 20 ka there was a hiatus in deposition at the site. This is indicated by the formation of another stalagmite on the surface of the erosional cut left in the Late Pleistocene deposits, with the growth occurring between 13.7 ka and 10.3 ka [39]. Following the formation of this speleothem on the cave floor a series of flowstone units (layers 3a-f) accumulated in the cut. The dated stalagmite was exposed by excavations in the Cave Mouth Trench; however, it is clear that the layer 3 sequence is laterally continuous across the baulk between the two trenches (see Fig 5C and Fig 6).

Layer 4b is a moderate brown (5YR4/4) slightly sandy mud with rare calcrete nodules. Reddish soil spots that are probably flecks of processed ironstone haematite (ochreous pigment) are prevalent. The mean grain size of layer 4b in the Shelter Trench is poorly sorted fine-medium grained sand, compared to very poorly sorted medium silt in the Cave Mouth Trench. Layer 4b follows the same orientation as layer 4a, tilting towards the east. In the Shelter Trench it varies in thickness from 10 to 25 cm. Thin layers of mollusc shells are occasionally found, extending towards the east. The lower boundary of layer 4b is demarcated by breccia concentrations, particularly in squares -J2 and -J1, forming an interface with layer 4c/d below. Layer 4c/d tilts towards the east and is characterized by very poorly sorted medium-grained sandy soil in the Shelter Trench and a dominant greyish brown colour (10 YR 5/2). It has only been encountered in the southern part of the Shelter Trench, extending from square -G2 to square -J2, and disappearing on the east side of the trench (squares -I1/2 and -J1/2).

In the Shelter Trench, layers 4b-e contain numerous cemented ashy deposits that take the form of discrete, horizontally inclined lenses that are up to 10 cm thick. These distinctive features comprise dense concentrations of stone artefacts, charred bones, burnt whole and fragmented shells, and other detritus, all cemented into a mass of consolidated, grey, ash-like sediment. Also present within the cemented masses are small fragments of hard reddish sediment that seem intuitively to comprise baked or fired clay based on visual characteristics. Some have markings on the outer surface that are possibly negative impressions of small twigs and other vegetable matter. These features are particularly dense in squares -C2 and -D2, where they bank up against the edge of the massive roof-fall block and in the central floor of the shelter (-H2 and -G2). Our working hypothesis is that these ashy deposits are the remnants of hearths that were cemented *in situ* by calcium carbonate-enriched dripwater.

The basal layer (layer 4e) in the Phase II occupation in the Shelter Trench has only been encountered on the southern side of the mass of roof-fall blocks. It ranges from 15 to 40 cm in thickness and seems to have formed in an erosional cut in underlying layer 5 (see below). In the western section of the Shelter Trench, the bottom of layer 4e is delimited by large limestone boulders more than 1 m wide. Compared to the preceding layers, layer 4e is denser and moister. Dark brown and grey sediment mixed with brownish-yellow clayey soil, characteristic of layer 5, is present. In addition to apparent intermixing of distinct sedimentary layers, there is a blending of finds from layer 4e with those from underlying layer 5. Occasionally, heavily fossilized bones and teeth, characteristic of layers 5 and below, occur in layer 4e alongside typical finds of Phase II, such as technologically distinct chert artefacts [30, 36]. These out-of-context finds were probably reworked from layer 5 during the erosional episode and incorporated into layer 4e. Reworking might also have occurred due to early Phase II inhabitants disturbing the existing cave floor surface (that is, the top part of layer 5).

**DATING**

*Radiocarbon dating of micro-organics*

We attempted to extract microscopic pollen and organic residues from layers 4a and 4b (Cave Mouth Trench), using twice the normal amount of sediment (8 g) because of the inorganic nature of the sediment samples. The following preparation stages were undertaken at the Monash University palynology laboratory: 1) sediment breakdown with Na₄P₂O₇; 2) removal of carbonates with HCl 10%; 3) removal of humic acids with KOH (20 minutes at 80°C); 4) sieving at 210 and 7 microns to remove material outside the major range of palynomorphs; 5) heavy liquid separation (sodium polytungstate) x 2 to remove material with a higher specific gravity than palynomorphs. No microscopic organic materials were recovered from the sediment samples (Ursula Pietrzak-Aniszewska, pers. comm., 8 November 2013).

*U-series dating of faunal remains*

We conducted laser ablation U-series analysis on faunal elements recovered *in situ* from the 2013 and 2014 excavations in the Cave Mouth Trench. Two sets of samples were analysed. First, a set comprising seven teeth (Samples LBB3-19) and one bone (Sample LBB18) was run in 2014 by MA (Fig 16); subsequently, a second set of ten teeth was run in 2015 by RG (Samples 3608-3617) (Fig 17). All U-series isotope analyses were measured using the laser ablation MC-ICP-MS system at The Australian National University’s (ANU) Research School of Earth Sciences. The details for laser ablation U-series analysis of skeletal materials are summarised in [40].

U-series analysis provides insights into when uranium migrates into a bone or tooth. This may happen a short time after the burial of the skeletal element, but there also may be later U-overprints that are difficult to recognise. As such, apparent U-series results from faunal remains have generally to be regarded as minimum age estimates. It is very difficult or impossible to evaluate by how much the U-series results underestimate the correct age of the sample. Laser ablation U-series age calculations are compromised if the U-concentrations are below about 0.5 ppm. Many spot analyses yield significant amounts of detrital ^232^Th, which may either derive from sediment material in pores, or diffusion from the outside (see example in [40]). When the elemental U/Th ratios drop below 300, the resulting U-series result may be influenced by detrital ^230^Th. In the data tables for the LBB samples (S3-4 Tables), U-concentrations of less than 0.5 ppm and U/Th elemental ratios below 300 are marked in bold and no individual age calculations were carried out in these instances.

U-series ages were calculated using the Isoplot program [41]. With teeth it is usually not possible to establish diffusion profiles as U may migrate along several pathways; for example, from the outside of the root, the worn occlusal surface, or pulp cavity, into the dentine. Thus, the analyses along a transect do not necessarily follow the U-diffusion path. Consequently, it would be questionable to use the dataset of a transect and apply the DA [42] or DAD diffusion models [43]. This condition applied to all but one sample (LBB17). Instead, we calculated closed system ages for each spot analysis as well as diffusion ages which assume continuous diffusion into the skeletal tissue [43], and calculated averages. Those samples with larger standard deviations in the age result indicate some scatter, which seemed the result of micro U-redistribution within the skeletal tissue [44, 45]. In some cases, U may leach from the outside of the skeletal tissue. This situation can be recognized either by (^230^Th/^238^U » ^234^U/^238^U, all isotope ratios are given as activity ratios) or increasing U-series ages towards the outside of the skeletal tissue combined with decreasing U concentrations. Samples that indicate leaching were discarded.

Sequential laser spot analyses were undertaken on cross sections of a total of 18 faunal elements. The results of the first set (LBB3-19) are given in S3 Table and of the second set (3608-3617) in S4 Table. The averages of the tracks of the samples in stratigraphical order are shown in S5 Table. It is obvious that the U-series age results are not all in stratigraphical order. However, the samples show a distinctive grouping of ^234^U/^238^U ratios. Changes in this isotopic ratio indicate different sources for the uranium that migrated into the skeletal tissue. In the upper section of the stratigraphic column, layers 4a and 5, the ^234^U/^238^U ratios are all <1, which is highly unusual [46]. In layer 8, several teeth have much higher ^234^U/^238^U ratios, from 1.09 to 1.34 (LBB10, 19, 3611, 3615), and their ages are significantly older than in those in the overlying and underlying layers. Samples LBB19, 3611, and parts of LBB10, show clear signs of U-leaching. In layer 10, the ^234^U/^238^U ratios return to values close to 1, increasing slightly in layer 11. Sample LBB18 shows an unusually large scatter between subsequent spot analyses and should perhaps be discarded. The lower stratigraphic units (i.e., layers 6a-13) are characterised by low energy stream channels which may have reworked some faunal material from higher elevations to the south. This case seems particularly to apply to the sampled teeth from layer 8, and perhaps sample LBB7, which was found in layer 5. The remaining teeth seem to have accumulated their uranium while embedded at the site. Closed system and diffusion results for these samples agree well within error. Thus, the assumptions with respect to the U-uptake mechanism have no effect on the age calculation. The apparent U-series ages are throughout equal or younger than the corresponding OSL ages, confirming that the U-series results have to be regarded as minimum age estimates.

**OPTICAL DATING METHOD**

Optical dating determines the time since last sunlight exposure of sediments by estimating the amount of natural radiation to which it was exposed during burial time (equivalent dose) and dividing this number by the annual radiation at the specific sample location. Using infrared stimulated luminescence (IRSL) methods, we obtained four reliable ages for the time since deposition of the potassium (K)-rich feldspar fraction of sediment samples from the deep deposits in the Shelter Trench and Cave Mouth Trench (layers 5, 9b and 10c). Further details are provided below and a full methodological description can be found in [47]. We followed a single-aliquot regenerative (SAR) protocol [48, 49], adapted to K-rich feldspar by the introduction of a two-step infrared (IR) stimulation at different temperatures [50]. Such a protocol, termed post-IR IRSL (pIRIR), depletes unstable signal during a first low-temperature IR stimulation (here, 100°C) and measures the more stable signal during a subsequent higher-temperature IR stimulation for age estimation (here, 275°C). The resulting signal can often be used for dating without further corrections for signal loss over the burial time (i.e., fading). However, due to the fast crystallisation of volcanic K-rich feldspar, such as that found in LBB, even the more stable pIRIR signal can still require corrections.

We adopted a standardised growth curve (SGC; [51-52]) approach to estimate the equivalent dose, which enables measurement time to be reduced by 80% or more. The establishment of the SGC also allows for the recent L_n_T_n_ method to be applied [55-57], which circumvents the issue of truncation of D_e_ values caused by the saturating shape of the dose response curve in the higher dose range by modelling the central tendency of the signal distribution rather than the D_e_ distribution. Additionally, we used “micro-aliquots”, in which 5‒10 individual grains occupy each hole on a 100-hole disc, to increase the yield of luminescent grains. Due to the low percentage of grains emitting a pIRIR signal, this approach provides effectively single-grain resolution at LBB.

We found that, in contrast to the high fading rate observed in a previous study for single aliquots containing hundreds of grains using IR diodes for stimulation [54], the micro-aliquot results obtained using an IR laser show a low fading rate. The reduced fading rate of the pIRIR signal using the new procedure is due to the selection of low-fading bright grains from a population of grains with mixed fading rates. Additionally, the presence of outliers and insufficiently bleached grains was considered in the choice of age models used for the determination of the equivalent dose. Thus, the new age determined for sample LBB-II supersedes the one determined using an isochron method by [54], though we note that the age estimates are indistinguishable at 1σ.

We dated the upper two samples by modelling the SGC D_e_ populations, as these samples are in the lower dose range of the SGC and do not suffer significantly from the issues associated with truncation (S4 Fig). Both samples had broad D_e_ distributions with a more pronounced lower-dose component, so we assume these samples were not well-bleached prior to burial. Hence, we used a minimum age model (MAM; [48]) to obtain D_e_ estimates for the lower-dose component, which is assumed to have been well-bleached prior to burial. Outliers in the D_e_ distributions were rejected using the normalised median absolute deviation (nMAD) with 1.4826 as the correction factor for a normal distribution [58-59] and using a cutoff value of 1.5 (i.e., any data point with a nMAD value larger than the cutoff is considered an outlier).

For the lower two samples, we modelled the re-normalised L_n_/T_n_ ratios (L_n_T_n_ method) and projected only the one modelled value onto the SGC to obtain the D_e_ used for dating. After outlier rejection (nMAD cutoff = 2.0, accounting for the lower spread of L_n_/T_n_ distributions), the re-normalised L_n_/T_n_ distributions of these samples were relatively tightly distributed and, so, were dated using the central age model (CAM; [48]).

We corrected the final D_e_ estimates for a residual dose of 10.4 ± 0.7 Gy (based on residual dose tests) and divided the result by the corresponding total dose rate for all samples (S6 Table). We also corrected the resulting ages for fading according to [53] using a g-value of 1.46 ± 0.61% per decade obtained from a micro-aliquot fading test and the code of [60] to correct ages accordingly. Since this model is only appropriate for the linear part of the dose-response curve, we consider that the fading-corrected ages are only reliable for samples whose D_e_ values are smaller than the SGC’s characteristic saturation dose (D_0_) of 778 Gy.

Environmental dose rates were estimated from the sum of individual dose rate components. Beta dose rates were determined directly from dried and powdered subsamples measured on a Risø GM-25-5 multicounter system. Gamma dose rates were measured directly by *in situ* gamma spectrometry with a NaI(Tl) detector (2-inch in diameter). We were not able to determine the gamma dose rate *in situ* for sample LBB-II collected in 2013 by the excavation team. The gamma dose rate of this sample was estimated by determining radionuclide concentrations of bulk material collected separately at the sampling location (dried and powdered) using thick-source alpha-counting for concentrations of U and Th and beta-counting for K concentrations. Cosmic ray dose rates were estimated following [61]. Internal dose rates were calculated considering a K-content of 11.5 ± 1.7 wt% and an assumed internal rubidium (Rb) concentration of 400 ± 100 ppm [62]. The K-content was estimated using quantitative evaluation of minerals using energy dispersive spectroscopy (QEM-EDS) measurements of 85 grains (180-212 µm) from LBB that pass all criteria to be included for D_e_ estimation and using the calibration of [63]. Dose rate and sampling information is summarised in S7 Table.

**VERTEBRATE FAUNA (PHASE I)**

In the following section we provide a detailed description of the main faunal taxa represented within the excavated assemblage. The Number of Identified Specimens (NISP) and Minimum Number of Individuals (MNI) are quantified in S8 Table.

*Anoas*

Vertebrate remains in the Phase I deposits are dominated by bovids (Fig 19). The subfamily Bovinae includes the extant two species of the anoa (*B. depressicornis* and *B. quarlesi*). There are still many uncertainties about the separation and distribution of the two species. The lowland anoa is the largest of the two, with a skull length of 290-300 mm (females) and 298-322 (males), and with horn cores that are triangular in cross-section, with marked transverse ridges and an external keel (183-260 mm long in the females, 271-373 mm in the males) [64-65]. In the mountain anoa the skull length varies between 244-290 mm, and the horns (146-199 mm long) are short and cylindrical in cross-section. In order to compare isolated teeth and skeletal elements of the extant anoas, measurements were taken on recent skulls stored in Naturalis, Leiden.

Bovini attributed to the recent mountain anoa, *B. quarlesi*, are more frequent in layer 5, and remain present further down to the base of the sequence in layer 13. The larger lowland anoa, *B. depressicornis* has its first appearance possibly slightly lower in spit 22 (layer 5) of square C3. This specimen represents a very large epistropheus that is heavily encrusted, hence no comparative measurements for this element were available. Confirmation that this epistropheus does represent a lowland anoa needs to be verified at a later stage, because no other relatively large-sized bovid remains were recognized among the bovid material from layer 5. The next appearances of the large-bodied species, *B. depressicornis*, are in layer 6, layer 8 and layer 10, where it co-occurs with the smaller *B. quarlesi*. Layer 13 only yielded few poorly preserved anoa specimens on which no reliable measurements could be taken.

The bovine remains in layer 5 mostly comprise small skeletal bones of the extremities, such as carpals, phalanges and tarsals, plus isolated teeth and teeth fragments. The bovine elements from the fluvial layers 8, 10 and 13 on the contrary represent mostly larger long bones, are often heavily fossilized and water-rolled, and also include skull fragments, broken horn cores and mandible fragments. There are several specimens in which the horn cores are attached to a large portion of the frontal.

The largest individual is represented by a partial skull with both horn cores, but lacking the lower part of the skull and dentition (the specimen was recovered at 2.8 m depth below surface [2.9 m BD] in square -A1, layer 6). The horncores are triangular in cross-section, with weakly convex surfaces near the base. The transverse diameter increases slightly in distal direction and halfway along the horn core the cross-section forms a straight-sided triangle with flattened surfaces. The dorsal surface of the horn cores lies in the plane of the frontals. The angle between dorsal and lateral surfaces is 90° near the base, but merges to an acute angle halfway, forming a prominent lateral keel. Both horn cores are weakly divergent near their base, and curved slightly inward, so that they run parallel near the tips. This configuration and shape—with the triangular cross-section and internal keel—is very similar to *B. depressicornis* [65]. In the LBB specimen the length of the right horn core, which is completely preserved, measures 259 mm (along the inner or medial side) and 317 mm when measured along the external side. Groves and Grubb [65] give the horn core length of male *B. depressicornis* as varying between 271-373 mm, compared to 183-260 mm in females. Thus, the LBB skull can be attributed to a male lowland anoa. The transverse diameter of the same horn core measured near the base amounts to 64 mm. This specimen is not surpassed by any of the other horn cores recovered from LBB to date.

A smaller partial skull with both horn cores preserved originates from layer 10d (square B2). It has a large portion of a frontal with the lateral edges broken. The tips of both horn cores are also broken, but the left horn is almost complete. The horn cores are not keeled but have a rounded cross-section. The left orbit is preserved with minor edge damage, and the supraorbital foramen is preserved. The right orbit is broken but the base of the eye socket is partly preserved. The fronto-parietal has some fractures, but the two parts have not moved significantly relative to each other. Posteriorly, the skull is broken at the supra-occipital suture. This skull is very similar in dimensions and in morphology of the horn cores to a recent male skull in the collection of Naturalis (Leiden) (specimen #1402 of the mountain anoa, *B. quarlesi*).

None of the skull and horn core fragments excavated from the deep deposits at LBB thus far are above the size ranges for the lowland anoa, *B. depressicornis*, but could be attributed to either one of the two extant species of Sulawesi. With further study of the dentition, however, some fossils of lowland anoa from the LBB deep deposits may possibly be referred to the newly proposed species of extinct anoa, *B. grovesi* [66].

*Suids*

Suinae remains are relatively abundant in layers 5, but are rare in the lower fluvial layers and altogether absent from layers 10b, 10c, and 10e. Only two molar fragments were recovered from layer 13. It has not been possible yet to identify most of the Suinae fossils to genus level, but some diagnostic specimens could be determined to genus. For example, elements that could be easily assigned to a genus were the characteristic curved tusks of babirusa lacking enamel bands, babirusa incisors lacking enamel, and the *Sus* molars and premolars with the more delicate and “wrinkled” enamel pattern. It appears that the youngest occurrence of babirusa is in layer 5, as evidenced by a well-preserved left upper canine (find nr. 2014-18) (Fig 19G). Another specimen is represented by a lower incisor from layer 10 (spit 50, sieve fraction), which is rather fragmented and weathered, lacking outer enamel. A lower incisor of *Sus* also originates from layer 10 (spit 50, square -A1). A large-sized suid upper canine fragment from layer 10 (square -A1, spit 50) could possibly belong to *Celebochoerus*. However, the fragment is longitudinally split, exposing the pulp cavity, and is water-rolled and poorly preserved, hence the identification is insecure.

*Proboscideans*

S9 Table lists all the proboscidean fossil remains recovered from the various squares. The stratigraphically highest specimens are in layer 5. The first tooth fragment attributable to *Stegodon* does not occur until layer 8 (square -A2, spit 40). Rare additional *Stegodon* tooth fragments are heavily water-rolled, and occur in the layer 10 strata and possibly in layer 13 (Fig 20B). The latter comprise rounded enamel fragments with double-layered enamel, with the outer enamel layer rather thick, which suggests *Stegodon*.

The non-stegodont specimens from LBB are provisionally assigned to *Palaeoloxodon* cf. *namadicus*, the Asian straight-tusked elephant. The Tribe Elephantini includes three genera: *Elephas*, *Palaeoloxodon* and *Mammuthus*. *Palaeoloxodon* was formerly regarded as a subgenus of *Elephas*, but based on skeletal evidence it is now thought to deserve a genus of its own [67], although it is difficult to distinguish from *Elephas* based on fragmentary molar material only. The best-preserved specimen which we have tentatively assigned to *P.* cf. *namadicus* is from layer 8 and comprises an incomplete right mandibular ramus with a partial molar still in place (Fig 20C). A flaked cobble tool was found near the specimen (Fig 22), but there seem to be no cut marks or other obvious signs of butchery. The complete molar from the opposite side of the same individual was found isolated in the adjacent square (Fig 20D). Another mandibular ramus of the left side is from layer 10b in square A2, and belonged to a juvenile individual, with the (complete) lower dP3 in use (Fig 20E).

The most informative specimen is the isolated lower molar from layer 8 (Fig 20D). The molar is complete, with the anterior 7 molar lamellae (or plates) worn. The root is broken but the base of the enamel crown is preserved on both sides. The wear figures of slightly worn lamellae 6-7 are tripartite, whereas in the anterior lamellae single wear figure have formed. The tripartite wear figure is fully merged in ridge 5. The tooth has a total of x11x lamellae (“x” indicating incompletely formed lamellae at the front or back). The total maximum length amounts to 161.4 mm. The average lamellar frequency (LF), which is the number of lamellae in 10 cm of the crown, is 6.6 (see [68] for the method used to measure the LF). The maximum transverse width occurs at the base of the 10^th^ lamella and equals 48.8 mm, giving a length/width ratio of 3.28 in this molar. Since the molar is not tapering posteriorly, it is definitely not the last molar or M3. The lateral and medial borders of the lamellae converge weakly in apical direction. The height of unworn lamellae is 95.6 mm in the most anterior unworn plate, and decreases to 88.5 mm in the last fully developed plate. The Height/Width index of the unworn plates varies between 187 and 204.3, which means that individual plates are about twice as high as they are wide. The double-layered enamel exposed in the wear figures is delicately folded, with a frequency of between 5-6 folds along one centimetre in a transverse direction. The enamel thickness (ET) varies between 1.5 and 2 mm, with the outer layer occupying about one third of the total ET. The dimensions of the present molar are clearly too big for the first 3 molars (dP2, dP3 and dP4). The length in the dP4 of *E. maximus* is generally below 150 mm [69], and also the width of individual plates generally still increases towards the back of the teeth in the dP4, which is formed when the animal is still growing. This leaves two possibilities for the current specimen: it represents either an M1 or an M2.

S9 Table lists the range in size measurements of modern *Elephas* *maximus* lower molars and those of *E. hysudrindicus* ([69, 70-71]; own measurements). The length of the molar from layer 8 in LBB falls within the range of the M1 of *E. maximus*, *P. namadicus*, and *E. hysudricus*. No lower M1 of *E. hysundrindicus* is known, but this large-bodied endemic *Elephas* species, exclusively known from Java, presumably had a similar size range for the M1 as the other taxa listed in S9 Table. It is also below the range for the M2 for the above-mentioned species, suggesting that the layer 8 specimen indeed represents an M1, unless it would belong to a locally evolved dwarfed species. In that case it could represent a small M2. However, since all other postcranial elements recovered from LBB are large-sized (see below), the latter explanation is unlikely, and thus this specimen is identified as a lower left M1 of a large-sized member of Elephantini.

The number of lamellae in the layer 8 individual amounts to 11x, which is below the range known for the lower M1 of the Asian elephant, *E. maximus* (12-17 lamellae; [69]). This indicates that the layer 8 individual represents a more primitive evolutionary stage of the general trend observed in elephants, namely the increase of the number of lamellae over time, which culminated in *E. maximus* and *M. primigenius*. The number of lamellae in the layer 8 individual is above the range seen in the Early Pleistocene Asian mainland species *E. hysudricus*, which also has much lower hypsodonty indices [71]. Comparison with the M1 of *P. namadicus* provides a full match, although it must be noted that no comparison with the M1 of the Javanese endemic *E. hysudrindicus* could be made. It is thus here tentatively attributed to *P.* cf. *namadicus*.

The right mandible fragment with partial M1 of the same individual as the M1 described above is heavily weathered and damaged in front and rear. The horizontal ramus is broken as is the ascending ramus. A large portion of the coronoid process is preserved. The bone surface is cracked and exfoliation of cortical bone has occurred at many places. Of the molar 9.5 plates are emerged from the alveole, whereas the anterior enamel of the anterior ridge is broken. Inside the alveole, which is exposed in the broken ascending ramus, several fragments of enamel remain that formed part of the succeeding molar (M2) being formed inside the alveole. Measurements that could be taken on this mandible fragment are the maximum width of the ramus at the level of the onset of the coronoid process (measurement M18 of [68]), the height of the horizontal ramus at the level of the anterior onset of the coronoid process (M6 of [68]), and the height between the basal plane and the coronoid apophyses (M4). These measurements are 109.4 mm, 97 mm and 160 mm, respectively. Measurements of a recent *E. maximus* mandible housed in the collection of the Geology Museum in Bandung and in a similar age stage at death, are given in S11 Table, together with measurements taken on a fossil adult mandible from Cijerah, West Java, thought to be Late Pleistocene in age. The size of the LBB mandible is very similar to the *E. maximus* mandible of the same dental wear age stage (young adult), but considerably smaller than a full-grown *E. maximus* mandible with the last molar (M3) in use.

A juvenile left mandible fragment with molar originates from square A2, layer 10b (~550 cm depth below surface [564 cm BD]) (Fig 20E). Only a small portion of the mandible remains, but the molar is complete and unworn. The anterior 2 plates were found separately at a slightly deeper level, but there is clearly a fit along the crown base with the molar fragment still in the mandible. Two isolated plate fragments that were not yet fully formed were also found in close association with the mandible fragment, and probably originate from the alveole. The tooth has 8x plates (or x7x, if the anterior plate is considered as an incompletely formed plate), a total length of 62.3 mm and a maximum width of 26.3 mm. The maximum height of 40.7 mm occurs at plate 6, giving a Height/Width index of 162. The LF amounts to 12.9. The molar has a strongly developed medial cleft, which indicates this specimen represents a dP3. In elephants the dP3 is already erupted at birth, and since the molar is unworn, the individual must have died shortly after birth. The length is near the upper ranges of the dP3 of *E. maximus* and *P. namadicus* and 3 mm smaller than the single dP3 known of *E. hysudrindicus* (S10 Table). The number of plates corresponds with the dP3 of *E. maximus*, but is one plate less than in *E. hysidrindicus* and *P. namadicus*. However, it should be noted that the values and range of the dP3 of the various species listed in S10 Table are based on limited material for the two fossil species. Moreover, the anterior ridge of the LBB specimen is not fully developed, and if the plate formula is considered at x7x it would match the amount of ridges in the fossil mainland species. With disregard to its closest taxonomic affinity, it is clear that the juvenile specimen from LBB is rather large-sized, similar to the young adult mandible originating from layer 8. It is here tentatively attributed to *P.* cf. *namadicus*.

Until more complete skulls or additional dental material of the LBB species are discovered for comparison, no definite answer can be given as to whether it represents *P.* cf. *namadicus* or should be considered as a distinct endemic species. *P. namadicus* is characterized by its distinct skull morphology, with a prominent overhanging frontal crest. Hooijer [72] attributed a molar fragment said to originate from Samarinda, Borneo, to *Palaeoloxodon* [= *Elephas*] cf. *namadicus*, and it makes sense to assume that the ancestral LBB elephant would have crossed the Makassar Strait to Sulawesi.

Dental remains from LBB that are attributable to *Stegodon* are all rather fragmented and heavily water-rolled. They are restricted to the fluvial layers 8, 10 and 13 (S9 Table) and it is therefore possible that these remains were reworked from older strata. Nevertheless, this example is the first dated record of Sulawesi stegodonts outside the fossil record of the Walanae Basin [73]. The largest fragment consists of an isolated ridge (square -A2, spit 40, sieve fraction). The ridge has the characteristic “roof”-shaped morphology that gave the genus its name, and it is as high (30 mm) as it is wide (31 mm). It could be from an upper or lower molar. The unworn ridge has 6 conules, with one of the central conules out of line, suggesting that this ridge represents the anterior one of a molar. Therefore, its width does not necessarily reflect the maximum width of the molar, which may have been larger. If the ridge represents a large-sized species such as *S. trigonocephalus*, it could be an anterior fragment of an upper or lower third or fourth molar (dP4 or M1). However, it could also represent a dP3 of a dwarfed species. The lateral or medial portion of a ridge originating from square -A1 (layer, spit 49, sieve fraction) shows the double-layered enamel in cross-section, which has a total thickness of 3.76 mm. Equal thickness of the outer and inner enamel layers is indicative of *Stegodon*.

**LITHIC TECHNOLOGY AT LEANG BURUNG 2**

There are key similarities between LBB and the lithic technology from the deep deposits (>~50 ka) at Leang Burung 2. The latter technology from Leang Burung 2 has been described briefly in the published literature [74]. Here we present new observations drawn from previously unpublished data [75]. As was the case at LBB [30], the stoneworkers responsible for the Lower Industry at Leang Burung 2 also overwhelmingly chose locally-available cobbles for flaking. Some 95% of the artefacts at the latter site are limestone, with volcanics and chert represented in small numbers (4% and 1% respectively). The limestone assemblage reflects a mixture of flake blank production from cobbles, and edge production on cobbles and flake blanks. A range of large- to medium-sized limestone blocks were chosen for reduction at Leang Burung 2. Limestone cores—cobbles with negative flake scars (*n* = 35)—measured up to 163 mm in maximum dimension (avg 91 ± 29 mm) and weighed up to 3.5 kg (avg 482 ± 614 g). Scar sizes are highly variable, reflecting a mixture of flake and edge production. Reduction was usually through the short axis of the limestone cobble—several flat cobbles were retouched in a similar manner to flakes (*n* = 6)—although a cobble’s long axis was also targeted on occasion. Flaking on limestone cores was often distributed in overlapping “sets” of two to four scars, often delivered unifacially, although isolated scars were also common. When sets or isolated scars were struck from different parts of a cobble, “multiplatform cores” were created (*n* = 6). More intensive bifacial flaking along one platform edge created “radial cores” (*n* = 9). Although morphologically dissimilar, these types reflect a common technological approach to reducing limestone cobbles.

Limestone flakes chosen for retouch at Leang Burung 2 averaged 59 ± 12 mm in maximum dimension. This finding is within the size range of scars on cores in the assemblage and the blanks for these were probably selected from among the larger flakes struck on-site. Retouched cobbles—cobble cores reduced unifacially by non-invasive flaking—were larger than retouched flakes, averaging 85 ± 21 mm in maximum dimension. Limestone flake retouching occurred on-site, as indicated by the recovery of 10 uniface retouching flakes.

Chert artefacts are present in small numbers in the Lower Industry at Leang Burung 2. Two chert cores were recovered. One is a single-platform core. Although small (5.6 g) and broken by weathering, clear evidence is present of attempts to strike flakes down the long axis of the stone. A small multiplatform core (25.3 g) was recovered that was reduced from two separate platform surfaces. The scars on this core are small, averaging 7 mm in largest dimension, and it appears to have been made on a large flake blank. The chert artefacts are considerably more reduced than the limestone artefacts. Volcanic artefacts (*n* = 13) are more numerous than chert and include core reduction flakes, a large retouched flake (52 mm long), and one uniface retouching flake. The largest dimension of volcanic flakes is, on average, 32 ± 11 mm, suggesting the reduction of relatively large cores, although none were recovered at Leang Burung 2 [75].

**REFERENCES**

1. Wentworth CK. A scale of grade and class terms for clastic sediments. J Geol 1922;30: 377-392.

2. Blott SJ, Pye K. Gradistat: A grain size distribution and statistics package for the analysis of unconsolidated sediments. Earth Surf Process Landf 2001;26: 1237-1248.

3. McDonald RC. Limestone morphology in South Sulawesi, Indonesia. Z Geomorphol. 1976; 26(suppl.): 79-91.

4. Bartstra GJ. Short history of the archaeological exploration of the Maros caves in South Sulawesi. Mod Quat Res SE Asia. 1998;15: 193-201.

5. Bulbeck D, Pasqua M, Di Lello A. Culture history of the Toalean of South Sulawesi, Indonesia. Asian Perspect. 2000;39: 71-108.

6. Bulbeck D, Sumantri I, Hiscock P. Leang Sakapao 1, a second dated Pleistocene site from South Sulawesi, Indonesia. Mod. Quat. Res. SE Asia. 2004; 18: 111–128.

7. Clason AT. Late Pleistocene/Holocene hunter-gatherers of Sulawesi. Palaeohistoria 1989;29: 67-76.

8. Eriawati Y. Lukisan di gua-gua karst Maros–Pangkep, Sulawesi Selatan: Gambaran penghuni dan matapencahariannya. Jakarta: Indonesian Ministry of Cultural Media Development; 2003.

9. Franssen CJH. Bijdrage tot de kennis van het Toaliaan op Zuid-Celebes. Tijdschr Bat Gen. 1949;83: 331-339.

10. Glover IC. Ulu Leang cave, Maros: A preliminary sequence of post-Pleistocene cultural development in South Sulawesi. Archipel. 1976;11: 113-154.

11. Glover IC. Survey and excavation in the Maros district, South Sulawesi, Indonesia: the 1975 field season. Bull Indo Pac Pre Hi. 1978;1: 60-103.

12. Glover IC. The effects of sink action on archaeological deposits in caves: An Indonesian example. World Archaeol. 1979;10: 302–317.

13. Glover IC. Leang Burung 2: An Upper Palaeolithic rock shelter in south Sulawesi, Indonesia. Mod Quat Res SE Asia. 1981;6: 1-38.

14. Hakim B, Nur M, Rustam. The sites of Gua Pasaung (Rammang-Rammang) and Mallawa: Indicators of cultural contact between the Toalian and Neolithic complexes in South Sulawesi. IPPA Bulletin 2009;29: 45-52.

15. Heyning N. Praehistorische vindplaatsen bij Maros in Z. Celebes. Tijdschr Aardr Gen. 1951;68: 21-30.

16. Leclerc P. Observations archéologiques dans quelques cavités des karsts de Maros et de Malawa (Sulawesi). In: Expédition Thaï-Maros 86. Toulouse: Association Pyreneenne de Speleologie; 1987. pp. 147-153.

17. Mulvaney DJ, Soejono RP. Archaeology in Sulawesi, Indonesia. Antiquity 1970;45: 26-33.

18. Mulvaney DJ, Soejono RP. The Australian-Indonesian archaeological expedition to Sulawesi. Asian Perspect. 1970;8: 163-177.

19. Sarasin P, Sarasin F. Reisen in Celebes II. C.W. Kreidel’s Verlag: Wiesbaden; 1905.

20. Simons A, Bulbeck D. Late Quaternary faunal successions in south Sulawesi. Mod Quat Res SE Asia 2004;18: 167-190.

21. van Heekeren HR. Rock-paintings and other prehistoric discoveries near Maros (South West Celebes). Laporan Tahunan Dinas Purbakala. 1952;1950: 22-35.

22. van Heekeren HR. The Stone Age of Indonesia. ’S-Gravenhage: Martinus Nijhoff; 1957.

23. van Heekeren HR. The Stone Age of Indonesia. The Hague: Martinus Nijhoff; 1972.

24. Russell JM, Vogel H, Konecky BL, Bijaksana S, Huang Y, Melles M, et al. Glacial forcing of central Indonesian hydroclimate since 60,000 y B.P. Proc Natl Acad Sci. 2014;111(14): 5100-5105.

25. Scroxton N, Gagan MK, Dunbar GB, Ayliffe LK, Hantoro WS, Shen C-C, et al. Natural attrition and growth frequency variations of stalagmites in southwest Sulawesi over the past 530,000 years. Palaeogeogr Palaeocl. 2016;441(4): 823-833.

26. Renema W, Troelstra SR. Larger foraminifera distribution on a mesotrophic carbonate

shelf in SW Sulawesi (Indonesia). Palaeogeogr Palaeocl 2001;175: 125-146.

27. Aubert M, Brumm A, Ramli M, Sutikna T, Saptomo EW, Hakim B, et al. Pleistocene cave art from Sulawesi, Indonesia. Nature 2014;514: 223-227.

28. Perston YL, Burhan B, Newman K, Hakim B, Oktaviana AA, Brumm A. Technology, subsistence strategies and cultural diversity in South Sulawesi, Indonesia, during the Toalean mid-Holocene period: Recent advances in research. J Indo-Pac Archaeol. 2021;45: 1-24.

29. Newman K, Hakim B, Oktaviana AA, Burhan B, McGahan DP, Brumm A. The missing deposits of South Sulawesi: New sources of evidence for the Pleistocene/Holocene archaeological transition. Archaeol Res Asia 2022;32: 100408.

30. Perston YL, Moore MW, Suryatman, Burhan B, Hakim B, Oktaviana AA, Lebe R, et al. Stone-flaking technology at Leang Bulu Bettue, South Sulawesi, Indonesia. Archaeol. Oceania 2022;57: 249-272.

31. Behrensmeyer AK. Time resolution in fluvial vertebrate assemblages. Paleobiology 1982;l8: 69-77.

32. Bello SM, Parfitt SA, Stringer C. Quantitative micromorphological analyses of cut marks produced by ancient and modern handaxes. J Archaeol Sci 2009; l36: 1869-1880.

33. Fisher JW. Bone surface modifications in zooarchaeology. J Archaeol Method Theory 1995;2: 7-68.

34. Pickering TR, Egeland CP. Experimental patterns of hammerstone percussion damage on bones: implications for inferences of carcass processing by humans. J Archaeol Sci 2006;33: 459-469.

35. Shipman P, Rose J. Early hominid hunting, butchering, and carcass-processing behaviors: Approaches to the fossil record. J Anthropol Archaeol. 1983;2: 57-98.

36. Perston YL. Transitions in stone-flaking technology in South Sulawesi, Indonesia. Ph.D. thesis, Griffith University. 2022.

37. Moore MW, Sutikna T, Jatmiko, Morwood MJ Brumm A. Continuities in stone flaking technology at Liang Bua, Flores, Indonesia. J Hum Evol. 2009;57(5): 503-526.

38. von Rintelen T, Stelbrink B, Marwoto RM, Glaubrecht M. A snail perspective on the biogeography of Sulawesi, Indonesia: origin and intra-island dispersal of the viviparous freshwater gastropod Tylomelania. PLoS ONE 2014;9(6): e98917.

39. Brumm A, Bulbeck D, Hakim B, Burhan B, Oktaviana AA, Sumantri I, et al. Skeletal remains of a Pleistocene modern human (*Homo sapiens*) from Sulawesi. PLoS ONE 2021;16(9): e0257273.

40. Grün R, Eggins S, Kinsley L, Moseley H, Sambridge M. Laser ablation U-series analysis of fossil bones and teeth. Palaeogeogr Palaeocl. 2014;416: 150-167.

41. Ludwig KR. User’s Manual for Isoplot 3.00, Manual. Berkeley: Berkeley Geochronology Center; 2003.

42. Pike AWG, Hedges REM, Van Calsteren P. U-series dating of bone using the diffusion-adsorption model. Geochim Cosmochim Acta 2002;66: 4273-4286.

43. Sambridge M, Grün R, Eggins S. U-series dating of bone in an open system: the diffusion-adsorption-decay model. Quat Geochronol. 2012;9: 42-53.

44. Grün R, Aubert M, Hellstrom J, Duval M. The challenge of direct dating old human fossils. Quatern Int. 2010;223-224: 87-93.

45. Duval M, Aubert M, Hellstrom J, Grün R. High resolution, LA-ICP-MS mapping of U and Th isotopes in an Early Pleistocene equid tooth from Fuente Nueva-3 (Orce, Andalusia, Spain). Quat Geochronol 2011;6: 458-467.

46. Cherdyntsev VV. Uranium-234. Jerusalem: Israel Program for Scientific Translations; 1971.

47. Sontag-González M, Li B, O’Gorman K, Burhan B, Hakim B, Brumm A. Survival of the brightest: pIRIR dating of volcanic sediments in Sulawesi, Indonesia, using micro-aliquots of K-rich feldspar. Quat Geochronol 2024;85: 101638.

48. Galbraith RF, Roberts RG, Laslett GM, Yoshida H, Olley JM. Optical dating of single and multiple grains of quartz from Jinmium rock shelter, northern Australia: Part I, experimental design and statistical models. Archaeometry 1999;41: 339-364.

49. Murray AS, Wintle AG. Luminescence dating of quartz using an improved single-aliquot regenerative-dose protocol. Radiat Meas 2000;32: 57-73.

50. Thomsen KJ, Murray AS, Jain M, Bøtter-Jensen L. Laboratory fading rates of various luminescence signals from feldspar-rich sediment extracts. Radiat Meas 2008;43: 1474-1486.

51. Li B, Jacobs Z, Roberts RG, Li S-H. Single-grain dating of potassium-rich feldspar grains: Towards a global standardised growth curve for the post-IR IRSL signal. Quat Geochronol 2018;45: 23-36.

52. Li B, Roberts RG, Jacobs Z, Li S-H. Potential of establishing a ‘global standardised growth curve’ (gSGC) for optical dating of quartz from sediments. Quat Geochronol 2015;27: 94-104.

53. Huntley DJ, Lamothe M. Ubiquity of anomalous fading in K-feldspars and the measurement and correction for it in optical dating. Can J Earth Sci 2001l38: 1093-1106.

54. Li B, Roberts RG, Brumm A, Guo YG, Hakim B, Ramli M, et al. IRSL dating of fast-fading sanidine feldspars from Sulawesi, Indonesia. Ancient TL. 2016;34(2): 1-13.

55. Li B, Jacobs Z, Roberts RG. Validation of the LnTn method for De determination in optical dating of K-feldspar and quartz. Quat Geochronol 2020; l58: 101066.

56. Li B, Jacobs Z, Roberts RG, Galbraith R, Peng J. Variability in quartz OSL signals caused by measurement uncertainties: Problems and solutions. Quat Geochronol 2017;41: 11-25.

57. Jacobs Z, Li B, Shunkov MV, Kozlikin MB, Bolikhovskaya NS, Agadjanian AK, Uliyanov VA, et al. Timing of archaic hominin occupation of Denisova Cave in southern Siberia. Nature 2019;565: 594-599.

58. Powell R, Hergt J, Woodhead J. Improving isochron calculations with robust statistics and the bootstrap. Chem Geol 2002;185: 191-204.

59. Rousseeuw PJ, Debruyne M, Engelen S, Hubert M. Robustness and outlier detection in chemometrics. Crit Rev Anal Chem 2006;36: 221-242.

60. Kreutzer S. calc_FadingCorr(): Apply a fading correction according to Huntley & Lamothe (2001) for a given g-value and a given tc. Function version 0.4.2. In: Kreutzer S, Burow C, Dietze M, Fuchs MC, Schmidt C, Fischer M, Friedrich J, editors. Luminescence: Comprehensive Luminescence Dating Data Analysis. R package version 0.9.7. (2020). <https://CRAN.R-project.org/package=Luminescence>.

61. Prescott JR, Hutton JT. Cosmic ray contributions to dose rates for luminescence and ESR dating: Large depths and long-term time variations. Radiat Meas 1994;23, 497-500.

62. Huntley DJ, Hancock RGV. The Rb contents of the K-feldspar grains being measured in optical dating. Ancient TL 2001;19: 43-46.

63. O’Gorman K, Brink F, Tanner D, Li B, Jacobs Z. Calibration of a QEM-EDS system for rapid determination of potassium concentrations of feldspar grains used in optical dating. Quat Geochronol 2021;61: 101123.

64. Groves C. Systematics of the Anoa (Mammalia, Bovidae). Beaufortia 1969;17: 1-12.

65. Groves C, Grubb P. Ungulate taxonomy. Baltimore: The John Hopkins University Press; 2011.

66. Rozzi R. A new extinct dwarfed buffalo from Sulawesi and the evolution of the subgenus Anoa: An interdisciplinary perspective. Quat Sci Rev. 2017;157: 188-205.

67. Larramendi A, Zhang H, Palombo MR, Ferretti MP. The evolution of *Palaeoloxodon* skull structure: Disentangling phylogenetic, sexually dimorphic, ontogenetic, and allometric morphological signals. Quat Sci Rev. 2020;229: 106090.

68. van den Bergh GD. The Late Neogene elephantoid-bearing faunas of Indonesia and their palaeozoogeographic implications: a study of the terrestrial faunal succession of Sulawesi, Flores and Java, including evidence for early hominid dispersal east of Wallace’s Line. Scripta Geologica 1999;117: 1-419.

69. Roth VL, Shoshani J. Dental identification and age determination in *Elephas maximus*. J Zool. 1988;214: 567-588.

70. Hooijer DA. Fossil proboscidea from the Malay Archipelago and India. Zool Verh. 1955;28: 1-146.

71. Maglio VJ. Origin and evolution of the Elephantidae. Trans Am Phil Soc., new series 1973;63(3): 1-149.

72. Hooijer DA. *Palaeoloxodon* cf. *namadicus* (Falconer et Cautley) from Borneo. Proc Kon Ned Akad Wet B. 1952;55: 395-398.

73. van den Bergh GD, Li B, Brumm A, Grün R, Yurnaldi D, Moore MW, et al. Earliest hominin occupation of Sulawesi, Indonesia. Nature 2016; 529 (7585): 208-211.

74. Brumm A, Hakim B, Ramli M, Aubert M, van den Bergh GD, Li Bo, et al. A reassessment of the early archaeological record at Leang Burung 2, a Late Pleistocene rock-shelter site on the Indonesian island of Sulawesi. PLOS ONE. 2018;13(4): e0193025.

75. Moore M, Perston YL, Fauzi R. Preliminary report on the stone artefact sequence at Leang Bulu Bettue, 2013 field season, Squares A1 and A2. Unpublished report 2014.
