# Supplementary material for: A near-continuous archaeological record of Pleistocene human occupation at Leang Bulu Bettue, Sulawesi, Indonesia
Source: PLoS One. 2025 Dec 23;20(12):e0337993. doi: 10.1371/journal.pone.0337993 (PMC12725638; doi:10.1371/journal.pone.0337993)
Supplement: S1 Table — (PDF) [file pone.0337993.s001.pdf]

S1 Table. X-Ray Difraction (XRD) results for Leang Bulu Bettue sediment samples.

Mineral/Mineral Group	Depth 62-66 cm	Depth 110-114 cm	Depth 182-186 cm
Goethite	< 1	6	6
Clay Mineral	7	17	4
Kaolinite	3	11	4
Serpentine	< 1	2	0
Chlorite	< 1	1	0
Annite – biotite - phlogopite	1	1	0
Illite	5	13	7
K-feldspar	19	26	6
Quartz	2	9	3
Calcite	60	2	69
Boehmite	1	7	1
Nordstrandite	0	2	0
Gibbsite	0	1	0
Gypsum	0	< 1	0
Hematite and/or pyrite	< 1	1	0
