# Supplementary material for: A near-continuous archaeological record of Pleistocene human occupation at Leang Bulu Bettue, Sulawesi, Indonesia
Source: PLoS One. 2025 Dec 23;20(12):e0337993. doi: 10.1371/journal.pone.0337993 (PMC12725638; doi:10.1371/journal.pone.0337993)
Supplement: S2 Table — (PDF) [file pone.0337993.s002.pdf]

S2 Table. Isotope geochemistry analysis results for Leang Bulu Bettue sediment samples.

**Squ.**	**Depth (cm)**	**$^{87}Sr/^{86}Sr$**	**$^{143}Nd/^{144}Nd$**	**$^{207}Pb/^{206}Pb$**	**$^{208}Pb/^{206}Pb$**	**$^{206}Pb/^{204}Pb$**	**$^{207}Pb/^{204}Pb$**
-C2	10-12	.708220	.512642	.81183	2.0037	19.3088	15.6756
-C2	100-102	.708183	.512645	.82246	2.0348	19.0130	15.6374
-C2	200-202	.708464	.512651	.82644	2.0454	18.9348	15.6484
