# Supplementary material for: A near-continuous archaeological record of Pleistocene human occupation at Leang Bulu Bettue, Sulawesi, Indonesia
Source: PLoS One. 2025 Dec 23;20(12):e0337993. doi: 10.1371/journal.pone.0337993 (PMC12725638; doi:10.1371/journal.pone.0337993)
Supplement: S3 Table — No age calculations were carried out for U concentrations of ≤0.5 ppm or U/Th ≤ 300, but not negative (indicated in red). Negative U/Th are due to the average background being higher than the specific measurement. All errors are 2σ. (PDF) [file pone.0337993.s003.pdf]

S3 Table. U-series results on bones and teeth from Leang Bulu Bettue. No age calculations were carried out for U concentrations of ≤0.5 ppm or U/Th ≤300, but not negative (indicated in red). Negative U/Th are due to the average background being higher than the specific measurement. All errors are 2σ.

**LBB-3**	U (ppm)	Th (ppb)	U/Th	$^{230}Th/^{238}U$	$^{230}Th/^{238}U$ error	$^{234}U/^{238}U$	$^{234}U/^{238}U$ error	CS Age (ka)	CS Age error (ka)	Diff Age (ka)	Diff Age error (ka)
1A	70.0	78	900	0.1989	0.0029	1.0912	0.0022	21.9	0.4	21.9	0.4
2A	70.0	55	1273	0.2084	0.0021	1.0958	0.0024	22.9	0.3	23	0.3
3A	68.9	31	2193	0.2064	0.0032	1.0901	0.0022	22.8	0.4	22.9	0.4
4A	67.7	21	3247	0.2056	0.0030	1.0892	0.0034	22.8	0.4	22.8	0.4
5A	67.9	13	5188	0.2026	0.0039	1.0903	0.0139	22.3	0.3	22.4	0.4
6A	65.4	6	10383	0.1857	0.0070	1.0892	0.0086	20.3	0.4	20.4	0.4
7A	63.0	4	14551	0.1696	0.0027	1.0915	0.1058	18.4	0.3	18.4	0.3

8A	70.1	7	9591	0.1881	0.0034	1.0912	11.0326	20.6	0.3	20.6	0.3
9A	73.0	10	7438	0.1928	0.0039	1.0929	0.0026	21.1	0.5	21.2	0.5
10A	74.9	9	8714	0.1878	0.0035	1.0876	0.0019	20.6	0.4	20.7	0.4
11A	74.9	11	6894	0.183	0.0029	1.0861	0.0019	20.1	0.3	20.1	0.3
12A	75.7	12	6384	0.1995	0.0038	1.0885	0.0019	22	0.4	22.1	0.4
13A	79.4	10	7951	0.2089	0.003	1.088	0.0023	23.2	0.4	23.2	0.4
14A	71.8	9	7845	0.2027	0.0037	1.0861	0.0018	22.5	0.5	22.5	0.5
15A	37.3	18	2048	0.1467	0.0032	1.0862	0.0029	15.8	0.4	15.8	0.4
16A	0.72	7	**102**	0.1391	0.083	1.0246	0.0423	n/a			
17A	0.66	8	**82**	0.1827	0.0982	1.0270	0.0375	n/a			
18A	**0.35**	6	**59**	0.3018	0.6786	1.0663	0.1268	n/a			
19A	**0.39**	7	**59**	0.2543	2.5759	1.0837	0.4438	n/a			

20A	**0.21**	5	**44**	0.1816	1.7890	1.0370	0.4128	n/a			
1B	82.8	77	1079	0.2705	0.0034	1.0888	0.002	31	0.5	31.2	0.5
2B	74.4	27	2740	0.2163	0.0024	1.0908	0.0017	24	0.3	24.1	0.3
3B	70.2	88	798	0.2206	0.0026	1.0898	0.0022	24.6	0.3	24.7	0.3
4B	65.8	25	2604	0.2098	0.0024	1.088	0.0018	23.3	0.3	23.4	0.3
5B	61.5	18	3349	0.1839	0.0024	1.0901	0.0025	20.1	0.3	20.2	0.3
6B	62.8	10	6074	0.1786	0.0032	1.0887	0.0022	19.5	0.4	19.5	0.4
7B	66.9	9	7087	0.1891	0.0033	1.0903	0.0023	20.7	0.4	20.8	0.4
8B	65.9	10	6422	0.184	0.0031	1.0884	0.0017	20.1	0.4	20.2	0.4
9B	63.3	13	4974	0.1822	0.0031	1.0888	0.0023	19.9	0.4	20	0.4
10B	68.0	11	6160	0.1926	0.0035	1.0884	0.0026	21.2	0.4	21.2	0.4
11B	72.6	8	9011	0.1986	0.0045	1.0868	0.0031	21.9	0.6	22	0.6

12B	71.5	12	5784	0.204	0.0051	1.0903	0.0042	22.5	0.6	22.6	0.6
13B	67.6	10	7087	0.1994	0.0039	1.0819	0.0065	22.2	0.5	22.2	0.5
14B	63.7	10	6135	0.1868	0.0026	1.0845	0.0031	20.6	0.3	20.6	0.3
15B	17.5	7	2485	0.1624	0.0047	1.0845	0.0038	17.7	0.6	17.7	0.6
16B	**0.18**	1	**209**	0.1388	0.2095	1.0549	0.0971	n/a			
17B	**0.20**	1	**167**	0.1763	2.6753	1.1219	0.4196	n/a			
18B	**0.18**	1	**171**	0.2244	15.637	1.1374	2.5134	n/a			
19B	0.53	8	**66**	0.2131	2.7845	1.0878	0.5631	n/a			
20B	**0.48**	7	**65**	0.2393	33.5781	1.0315	3.5424	n/a			

**LBB-7**	U (ppm)	Th (ppb)	U/Th	$^{230}Th/^{238}U$	$^{230}Th/^{238}U$ error	$^{234}U/^{238}U$	$^{234}U/^{238}U$ error	CS Age (ka)	CS Age error (ka)	Diff Age (ka)	Diff Age

									error (ka)
1A	1.83	4795	**0**	1.4173	0.0843	1.1750	0.0179	n/a	
2A	16.7	7300	**2**	1.5077	0.0191	1.3299	0.0048	n/a	
3A	77.5	72	1082	1.6112	0.0191	1.3528	0.0046	leaching	
4A	55.2	37	1495	1.3743	0.0089	1.3267	0.0024	leaching	
5A	29.8	33	910	1.6503	0.0170	1.2764	0.0027	leaching	
6A	12.9	27	481	1.4577	0.0185	1.2089	0.0050	leaching	
7A	22.8	36	638	1.5688	0.0205	1.2160	0.0039	leaching	
1B	2.22	4765	**0**	1.5285	0.0417	1.1686	0.0115	n/a	
2B	14.1	5689	**2**	1.4827	0.0277	1.3338	0.0053	n/a	
3B	49.6	60	830	1.5441	0.0243	1.3567	0.0039	leaching	

4B	67.7	48	1414	1.6877	0.0324	1.3247	0.0027	leaching
5B	19.7	35	558	1.4132	0.0157	1.2754	0.0049	leaching
6B	16.8	20	825	1.4726	0.0253	1.2154	0.0042	leaching
7B	26.0	33	786	1.5743	0.0164	1.2177	0.0041	leaching
1C	64.6	220	**293**	1.4423	0.0108	1.3535	0.0024	n/a
2C	67.2	48	1415	1.729	0.046	1.3442	0.0029	leaching
3C	61.6	29	2144	1.6858	0.0283	1.3229	0.0022	leaching
4C	11.2	16	710	1.4461	0.0269	1.2261	0.0042	leaching
5C	18.7	20	958	1.5666	0.0207	1.215	0.0038	leaching
6C	25.7	31	825	1.6815	0.0145	1.2242	0.0034	leaching

**LBB-10**	U (ppm)	Th (ppb)	U/Th	$^{230}Th/^{238}U$	$^{230}Th/^{238}U$ error	$^{234}U/^{238}U$	$^{234}U/^{238}U$ error	CS Age (ka)	CS Age error (ka)	Diff Age (ka)	Diff Age

									error (ka)
1A	122.2	8	16231	2.6980	0.0616	1.4947	0.0021	leaching	
2A	112.3	7	16973	2.3287	0.0345	1.4941	0.0024	leaching	
3A	102.2	8	12602	2.0643	0.0171	1.4965	0.0023	leaching	
4A	112.2	5	24808	2.3159	0.0413	1.4955	0.0027	leaching	
5A	107.1	4	25065	2.2156	0.0232	1.4996	0.0027	leaching	
6A	111.3	6	18822	2.3114	0.0300	1.5049	0.0025	leaching	
7A	110.4	8	13026	2.3307	0.0353	1.5033	0.0023	leaching	
8A	95.7	4	22342	2.1824	0.0247	1.5084	0.0026	leaching	
9A	93.3	3	28669	2.2462	0.0172	1.5209	0.0033	leaching	
10A	73.6	7	10075	2.1898	0.0224	1.5309	0.0031	leaching	

11A	13.2	6	2108	0.1932	0.0058	1.2755	0.0057	17.8	0.6	17.9	0.6
12A	21.1	4	5755	0.2776	0.0061	1.2942	0.0040	26.1	0.7	26.3	0.7
13A	21.7	5	4772	1.2110	0.0141	1.4363	0.0042	171.6	4.6	202	7.3
1B	112.3	5	21792	2.7911	0.0656	1.4996	0.0019	leaching			
2B	90.9	6	15978	2.1890	0.0259	1.5018	0.0023	leaching			
3B	96.6	5	18204	2.2298	0.0175	1.5003	0.0027	leaching			
4B	105.8	4	24447	2.2660	0.0234	1.4963	0.0031	leaching			
5B	101.2	6	17690	2.1100	0.0177	1.4921	0.0024	leaching			
6B	105.4	6	18418	2.2693	0.0215	1.4972	0.0025	leaching			
7B	103.8	3	34387	2.2374	0.0191	1.5003	0.0024	leaching			
8B	98.2	4	23885	2.2010	0.0189	1.5023	0.0030	leaching			
9B	86.1	8	10550	2.2285	0.0306	1.5109	0.0024	leaching			

10B	87.9	13	7009	2.3341	0.0216	1.5153	0.0026	leaching				
11B	8.9	5	1866	0.2590	0.0099	1.2903	0.0065	24.2	1	24.4	1.1
12B	21.0	3	8057	0.2503	0.0065	1.2925	0.0056	23.3	0.7	23.5	0.7
13B	26.7	3	8082	1.3069	0.0105	1.4289	0.0038	208.5	6.6	268.3	14.7
1C	69.3	5	14762	2.4250	0.0259	1.5046	0.0027	leaching			
2C	28.8	1	8659	2.6858	0.0397	1.5068	0.0027	leaching			
3C	151.4	5	15123	2.6299	0.0358	1.5087	0.0023	leaching			
4C	101.5	5	21475	2.5182	0.0311	1.5055	0.0029	leaching			
5C	106.6	5	20207	2.4671	0.0283	1.5009	0.0021	leaching			
6C	93.2	8	11361	2.2062	0.0233	1.4944	0.0026	leaching			
7C	89.9	5	17461	2.1700	0.0222	1.4955	0.0021	leaching			
8C	90.3	4	21540	2.1696	0.0252	1.5004	0.0026	leaching			

9C	85.7	5	17234	2.0913	0.0179	1.4953	0.0024	leaching				
10C	73.0	7	10000	2.2075	0.0108	1.4921	0.0039	leaching				
11C	9.7	4	2275	0.6289	0.0297	1.3372	0.0079	67.2	4.4	69.3	4.7	
12C	18.5	5	3984	0.3008	0.0070	1.2976	0.0051	28.5	0.8	28.8	0.8	
13C	24.9	6	4397	1.0520	0.0142	1.4018	0.0044	136.9	3.6	151.4	4.6	

**LBB-13**	U (ppm)	Th (ppb)	U/Th	$^{230}Th/^{238}U$	$^{230}Th/^{238}U$ error	$^{234}U/^{238}U$	$^{234}U/^{238}U$ error	CS Age (ka)	CS Age error (ka)	Diff Age (ka)	Diff Age error (ka)
1A	3.42	60	**57**	0.4003	0.0147	1.2514	0.0120	n/a			
2A	28.2	298	**95**	0.3143	0.0069	1.2370	0.0030	n/a			
3A	106.2	2	42717	0.2536	0.0048	1.2379	0.0018	24.8	0.5	25.0	0.5
4A	99.4	3	34823	0.2637	0.0041	1.2413	0.0017	25.8	0.5	26.0	0.5
5A	88.5	1	67065	0.2835	0.0036	1.2416	0.0020	28.0	0.4	28.3	0.4
6A	82.6	2	54043	0.2945	0.0037	1.2407	0.0021	29.3	0.4	29.5	0.4
7A	3.67	2	2206	0.2589	0.0141	1.2557	0.0088	25.0	1.5	25.2	1.6
8A	4.51	2	1842	0.1328	0.0088	1.2567	0.0074	12.1	0.9	12.2	0.9
9A	7.79	5	1564	0.2395	0.0065	1.2736	0.0057	22.5	0.7	22.7	0.7

1B	3.11	120	**26**	0.6295	0.0216	1.2461	0.0090	n/a				
2B	71.4	289	**247**	0.2658	0.0046	1.2373	0.0019	n/a				
3B	105.8	6	18440	0.2626	0.0039	1.2389	0.0016	25.8	0.4	26.0	0.4
4B	93.6	2	42193	0.3205	0.0051	1.2398	0.0020	32.3	0.6	32.6	0.6
5B	84.7	1	66063	0.3033	0.0045	1.2426	0.0021	30.2	0.5	30.5	0.5
6B	78.0	1	52340	0.3074	0.0041	1.2429	0.0018	30.7	0.5	31.0	0.5
7B	0.88	9	**103**	0.2989	0.0286	1.2918	0.0168	n/a				
8B	4.44	2	1809	0.1237	0.0079	1.2444	0.0072	11.4	0.8	11.4	0.8
9B	8.63	5	1791	0.2281	0.0074	1.2744	0.0053	21.4	0.8	21.5	0.8
1C	70.3	231	304	0.3070	0.0052	1.2406	0.0021	30.7	0.6	31.0	0.6
2C	92.3	9	9918	0.2933	0.0036	1.2414	0.0022	29.1	0.4	29.4	0.4
3C	99.3	2	43810	0.2997	0.0031	1.2398	0.0022	29.9	0.4	30.2	0.4

4C	84.7	19	4501	0.3217	0.0047	1.2419	0.0020	32.4	0.6	32.7	0.6
5C	77.5	2	44068	0.3121	0.0044	1.2427	0.0025	31.2	0.5	31.5	0.5
6C	13.6	1	9256	0.3264	0.0092	1.2409	0.0053	32.9	1.1	33.3	1.1
7C	1.38	2	864	0.2703	0.0157	1.2480	0.0112	26.4	1.8	26.6	1.8
8C	5.89	3	2111	0.1224	0.0062	1.2579	0.0070	11.1	0.6	11.2	0.6
9C	10.0	5	1877	0.2570	0.0068	1.2763	0.0045	24.3	0.7	24.5	0.7

**LBB-15**	U (ppm)	Th (ppb)	U/Th	$^{230}Th/^{238}U$	$^{230}Th/^{238}U$ error	$^{234}U/^{238}U$	$^{234}U/^{238}U$ error	CS Age (ka)	CS Age error (ka)	Diff Age (ka)	Diff Age error (ka)
1A	80.0	3	30471	0.1234	0.0031	1.1626	0.0018	12.2	0.3	12.2	0.3
2A	79.3	3	29924	0.1207	0.0030	1.1572	0.0021	12.0	0.3	12.0	0.3
3A	70.8	1	62331	0.1137	0.0034	1.1584	0.0022	11.2	0.4	11.3	0.4
4A	70.9	1	67144	0.1064	0.0041	1.1586	0.0020	10.5	0.4	10.5	0.4
5A	70.7	1	60733	0.1008	0.0031	1.1567	0.0024	9.9	0.3	9.9	0.3
6A	67.8	1	66014	0.0990	0.0035	1.1543	0.0020	9.8	0.4	9.8	0.4
7A	66.1	3	19720	0.1052	0.0033	1.1572	0.0024	10.4	0.3	10.4	0.3
8A	70.7	2	33017	0.1022	0.0021	1.1565	0.0022	10.1	0.2	10.1	0.2
9A	23.2	4	5441	0.0856	0.0046	1.1640	0.0030	8.3	0.5	8.3	0.5

10A	0.54	5	**112**	0.3343	0.0417	1.1943	0.0238	n/a				
11A	0.87	4	**243**	0.1208	0.0278	1.1719	0.0185	n/a				
12A	1.26	3	391	0.1203	0.0178	1.1863	0.0183	11.6	1.8	11.7	1.8
1B	67.6	10	6940	0.1239	0.0025	1.1668	0.0020	12.2	0.3	12.2	0.3
2B	80.2	2	33192	0.1249	0.0028	1.1679	0.0019	12.3	0.3	12.3	0.3
3B	81.3	1	115999	0.1226	0.0029	1.1714	0.0022	12.0	0.3	12.1	0.3
4B	77.7	1	98581	0.1192	0.0033	1.1688	0.0021	11.7	0.3	11.7	0.3
5B	75.7	0	153497	0.1115	0.0025	1.1688	0.0022	10.9	0.3	10.9	0.3
6B	73.3	2	30057	0.1088	0.0027	1.1679	0.0023	10.6	0.3	10.7	0.3
7B	76.0	1	103553	0.1086	0.0034	1.1667	0.0020	10.6	0.4	10.7	0.4
8B	69.2	2	40266	0.1034	0.0027	1.1648	0.0022	10.1	0.3	10.1	0.3
9B	65.3	1	48180	0.1121	0.0021	1.1612	0.0016	11.0	0.2	11.1	0.2

10B	0.61	1	570	0.1588	0.0397	1.1802	0.0301	15.7	4.3	15.8	4.3
11B	1	2	627	0.1391	0.0230	1.1691	0.0207	13.8	2.5	13.8	2.5
12B	1.55	4	411	0.1379	0.0197	1.1817	0.0149	13.5	2.1	13.5	2.1
1C	67.6	1	63713	0.1107	0.0025	1.1693	0.0021	10.8	0.3	10.8	0.3
2C	91.1	1	62089	0.1391	0.0039	1.1667	0.0024	13.8	0.4	13.8	0.4
3C	83.5	1	65784	0.1098	0.0023	1.1651	0.0017	10.8	0.2	10.8	0.2
4C	73.8	1	72742	0.1143	0.0034	1.1648	0.0022	11.2	0.3	11.3	0.4
5C	71.7	2	34818	0.1040	0.0027	1.1632	0.0020	10.2	0.3	10.2	0.3
6C	74.5	1	52617	0.1086	0.0028	1.1659	0.0023	10.6	0.3	10.7	0.3
7C	71.1	1	62865	0.1090	0.0024	1.1693	0.0019	10.7	0.2	10.7	0.2
8C	75.3	1	53250	0.0914	0.0025	1.1639	0.0025	8.9	0.3	8.9	0.3
9C	1.24	2	661	0.1362	0.0155	1.1748	0.0164	13.4	1.6	13.4	1.7

10C	0.83	1	882	0.1267	0.0270	1.1975	0.0265	12.2	2.8	12.2	2.8
11C	0.86	4	**197**	0.1273	0.0221	1.1790	0.0225	n/a			
12C	67.6	1	63713	0.1107	0.0025	1.1693	0.0021	10.8	0.3	10.8	0.3

**LBB-17**	U (ppm)	Th (ppb)	U/Th	$^{230}Th/^{238}U$	$^{230}Th/^{238}U$ error	$^{234}U/^{238}U$	$^{234}U/^{238}U$ error	CS Age (ka)	CS Age error (ka)	Diff Age (ka)	Diff Age error (ka)
1A	1.08	254	**4**	2.2166	0.0905	1.3114	0.0206	n/a			
2A	12.7	762	**17**	1.3485	0.0226	1.2822	0.0067	n/a			
3A	85.2	6	14564	1.1220	0.0216	1.3229	0.0021	179	8	206	12
4A	95.4	5	17704	1.0129	0.0174	1.3175	0.0025	145	5	160	6
5A	103.8	3	31975	1.0860	0.0173	1.3154	0.0017	169	6	191	9
6A	97.9	2	55269	1.0070	0.0081	1.3138	0.0025	145	2	159	3
7A	104.1	3	31452	1.0102	0.0100	1.3070	0.0018	147	3	162	4
8A	110.8	3	42690	1.0573	0.0094	1.3085	0.0016	161	3	180	4
9A	100.2	7	14889	0.9479	0.0113	1.3094	0.0018	130	3	140	4

10A	91.9	7	12864	0.9578	0.0110	1.3086	0.0020	133	3	144	4
11A	24.5	4	5930	0.7946	0.0140	1.3251	0.0045	95.1	2.6	99.8	3.0
12A	19.5	7	2878	0.7284	0.0184	1.3302	0.0055	83.0	3.1	86.5	3.4
13A	20.0	4	5358	0.7988	0.0126	1.3365	0.0049	94.5	2.4	99.3	2.7
14A	15.5	195	**79**	1.0470	0.0172	1.3475	0.0054	n/a			
1B	7.47	430	**17**	1.4035	0.0365	1.2548	0.0092	n/a			
2B	74.1	115	643	1.0826	0.0075	1.3168	0.0024	167	3	188	4
3B	99.2	2	40910	1.1193	0.0175	1.3219	0.0022	178	7	205	10
4B	97.2	2	48566	0.9752	0.0054	1.3216	0.0021	134	2	146	2
5B	95.6	2	51797	0.9424	0.0039	1.3074	0.0020	129	1	139	1
6B	101.4	2	54464	0.9425	0.0043	1.3085	0.0018	129	1	139	1
7B	96.3	4	25071	0.8997	0.0037	1.3113	0.0019	118	1	126	1

8B	95.3	4	24978	0.8964	0.0070	1.3081	0.0020	118	2	126	2
9B	59.2	4	16197	1.0124	0.0088	1.3100	0.0026	147	3	162	3
10B	18.8	6	3207	0.6719	0.0108	1.339	0.0052	73.3	1.7	76.0	1.8
11B	19.0	11	1761	0.7943	0.0170	1.3373	0.0040	93.6	3.1	98.3	3.5
12B	13.2	125	**105**	1.0363	0.0206	1.3442	0.0079	n/a			
1C	2.34	777	**3**	0.7623	0.0279	1.2898	0.0161	n/a			
2C	59.0	411	**143**	1.2342	0.0124	1.3023	0.0022	n/a			
3C	95.5	6	17085	1.094	0.0070	1.3161	0.0022	171	3	194	4
4C	102.6	2	42016	1.1005	0.0082	1.3189	0.0020	172	3	196	4
5C	100.4	7	13853	1.0688	0.0058	1.3124	0.0021	164	2	184	3
6C	100.0	3	29640	0.9589	0.0048	1.3132	0.0017	132	1	143	2
7C	103.2	2	47470	0.9838	0.0071	1.3099	0.0017	139	2	152	2

8C	99.6	2	61101	0.9927	0.0088	1.3083	0.0019	142	3	155	3
9C	45.7	1	64181	0.9108	0.0093	1.3182	0.0031	120	2	128	3
10C	19.7	3	7128	0.6683	0.0093	1.3405	0.0044	72.7	1.4	75.3	1.6
11C	19.5	2	8181	0.7554	0.0093	1.3435	0.0052	86.2	1.7	90.1	1.8
12C	13.9	46	300	0.9755	0.0166	1.3415	0.0051	130	4	142	5

**LBB-18**	U (ppm)	Th (ppb)	U/Th	$^{230}Th/^{238}U$	$^{230}Th/^{238}U$ error	$^{234}U/^{238}U$	$^{234}U/^{238}U$ error	CS Age (ka)	CS Age error (ka)	Diff Age (ka)	Diff Age error (ka)
1A	4.33	4	1148	0.7891	0.0202	1.2234	0.0064	108	5	113	5
2A	3.26	3	1113	0.4984	0.0193	1.2159	0.0093	56.5	2.9	57.5	3.0
3A	1.35	2	862	0.4170	0.0293	1.2157	0.0107	45.2	4.0	45.8	4.1
4A	1.20	2	559	0.6452	0.0307	1.1683	0.0156	85.5	6.5	87.6	6.9
5A	7.95	1	8421	0.4357	0.0123	1.1281	0.0062	52.7	1.9	53.2	2.0
6A	11.8	1	12825	0.4760	0.0087	1.1519	0.0051	57.3	1.4	58.1	1.4
7A	12.8	1	14030	0.6964	0.0128	1.1865	0.0052	93.5	2.8	96.4	3.0
8A	13.9	2	8220	0.7602	0.0121	1.1999	0.0048	105	3	110	3
9A	13.8	1	10074	0.8531	0.0160	1.2198	0.0048	124	4	131	5

10A	12.4	2	8031	0.6698	0.0105	1.1982	0.0050	86.7	2.1	89.3	2.3
11A	13.3	1	9827	0.6091	0.0090	1.1896	0.0043	76.4	1.7	78.2	1.8
12A	12.4	2	7324	0.4683	0.0099	1.1707	0.0044	54.9	1.5	55.7	1.6
13A	5.33	3	2072	0.5418	0.0156	1.2065	0.0059	63.7	2.5	65.0	2.6
14A	12.6	1	9248	0.2735	0.0065	1.2358	0.0059	27.1	0.7	27.3	0.8
15A	23.4	2	12849	0.4404	0.0080	1.2768	0.0038	45.4	1.0	46.1	1.1
16A	25.1	2	12724	0.4436	0.0061	1.2713	0.0033	46.0	0.8	46.8	0.8
1B	1.43	6	**234**	1.2628	0.0496	1.2162	0.0123	n/a			
2B	0.51	4	**130**	1.4195	0.0831	1.2322	0.0271	n/a			
3B	0.81	3	**259**	1.2410	0.0663	1.1657	0.0246	n/a			
4B	10.4	3	3141	0.4602	0.0106	1.1176	0.0042	57.3	1.8	57.9	1.8
5B	12.4	3	3933	0.4372	0.0090	1.1171	0.0042	53.6	1.4	54.2	1.5

6B	14.3	2	5840	0.5719	0.0111	1.1453	0.0048	74.1	2.1	75.4	2.2
7B	14.9	2	8110	0.7186	0.0102	1.1799	0.0048	99.2	2.4	102.4	2.5
8B	15.8	2	8913	0.8931	0.0126	1.2109	0.0055	137	4	146	5
9B	15.8	2	7819	0.6726	0.0113	1.1785	0.0048	89.7	2.4	92.2	2.5
10B	14.3	2	5966	0.5832	0.0121	1.1776	0.0040	73.0	2.2	74.5	2.3
11B	14.8	2	8655	0.5044	0.0116	1.1551	0.0043	61.6	1.9	62.6	2.0
12B	10.8	3	4279	0.4539	0.0099	1.1624	0.0048	53.3	1.5	54.0	1.6
13B	2.41	2	1171	0.3184	0.0217	1.1842	0.0106	33.9	2.7	34.2	2.8
14B	25.5	3	8908	0.3183	0.0061	1.2437	0.0034	31.9	0.7	32.2	0.7
15B	32.9	2	15202	0.4254	0.0085	1.2594	0.0035	44.3	1.1	44.9	1.1
16B	26.7	3	8951	0.4427	0.0083	1.2559	0.0033	46.6	1.1	47.4	1.1

**LBB-18**	U (ppm)	Th (ppb)	U/Th	$^{230}Th/^{238}U$	$^{230}Th/^{238}U$ error	$^{234}U/^{238}U$	$^{234}U/^{238}U$ error	CS Age (ka)	CS Age error (ka)	Diff Age (ka)	Diff Age error (ka)
1C	6.05	3	2199	0.4945	0.0166	1.2303	0.0067	55.0	2.4	56.0	2.5
2C	3.48	2	1462	0.3064	0.0140	1.2171	0.0098	31.3	1.7	31.6	1.7
3C	0.16	1	**294**	1.5502	0.2943	1.3742	0.0690	n/a			
4C	11.8	2	6056	1.1372	0.0157	1.2718	0.0044	207	8	245	14
5C	21.1	3	7858	1.0744	0.0116	1.2778	0.0029	177	5	200	7
6C	25.7	2	13434	1.8709	0.0162	1.3392	0.0043	0.0	0.0	0.0	0.0
7C	15.1	4	4207	1.3246	0.0195	1.2815	0.0040	354	36	0	0
8C	12.5	5	2284	0.7328	0.0143	1.1932	0.0048	100	3	104	4
9C	12.9	3	5124	0.7692	0.0132	1.2032	0.0049	107	3	111	4

10C	14.4	5	2723	1.0485	0.0183	1.2577	0.0047	175	7	196	10
11C	30.2	2	14947	1.3998	0.0157	1.3541	0.0038	330	21	leaching	
12C	1.74	1	1266	1.0588	0.0363	1.2391	0.0150	187	18	210	26
13C	25.3	2	13153	0.7392	0.0118	1.2775	0.0042	90.5	2.2	94.2	2.5
14C	46.9	4	12202	0.8554	0.0131	1.2940	0.0028	111	3	118	3
15C	42.5	10	4231	0.8069	0.0128	1.2990	0.0033	101	3	106	3
1E	18.3	1	25684	0.5083	0.0063	1.0200	0.0029	75.0	1.4	75.2	1.4
2E	17.7	1	29377	0.3710	0.0040	0.9557	0.0026	53.8	0.8	53.6	0.8
3E	17.6	3	6251	0.3689	0.0041	0.9376	0.0036	54.9	0.9	54.5	0.8
4E	17.1	0	79727	0.3380	0.0072	0.9407	0.0050	48.8	1.4	48.6	1.4
5E	18.1	0	71686	0.4753	0.0113	0.9788	0.0069	72.7	2.6	72.5	2.6
6E	35.4	1	37493	0.9621	0.0083	1.1023	0.0074	209	7	225	9

7E	33.2	0	86384	0.9166	0.0118	1.0500	0.0161	216	16	225	16
1F	7.88	0	67161	0.3860	0.0075	0.9493	0.0098	57.2	1.7	56.9	1.7
2F	3.49	0	-730141	0.4839	0.0099	0.9832	0.0098	74.1	2.5	73.9	2.4
3F	0.61	0	2942	0.4968	0.0421	1.0074	0.0258	74.0	9.9	74.1	9.8
4F	0.73	0	1895	0.8450	0.0379	0.9884	0.0188	212	39	211	37
5F	0.57	0	1367	0.8186	0.0429	0.9744	0.0176	204	40	200	38
6F	1.53	0	-6474	0.4487	0.0149	0.9407	0.0110	71.3	3.7	70.7	3.6
7F	2.95	0	111587	0.4208	0.0322	0.9242	0.0083	66.9	7.4	66.2	7.3

**LBB-19**	U (ppm)	Th (ppb)	U/Th	$^{230}Th/^{238}U$	$^{230}Th/^{238}U$ error	$^{234}U/^{238}U$	$^{234}U/^{238}U$ error	CS Age (ka)	CS Age error (ka)	Diff Age (ka)	Diff Age error (ka)

1A	2.80	3971	**1**	1.3767	0.0715	1.3866	0.0182	n/a	
2A	1.28	3566	**0**	1.4030	0.0943	1.3278	0.0242	n/a	
3A	7.32	2553	**3**	1.0191	0.0347	1.3873	0.0090	n/a	
4A	2.69	785	**3**	0.9180	0.0405	1.2855	0.0121	n/a	
5A	4.04	12	344	1.6213	0.0400	1.4449	0.0122	574	280
6A	67.6	161	421	2.2054	0.0132	1.6051	0.0024	leaching	0
7A	69.0	8	9205	2.4673	0.0250	1.6029	0.0023	leaching	0
8A	72.6	7	10545	2.5643	0.0166	1.5952	0.0027	leaching	0
9A	67.7	9	7805	2.6223	0.0173	1.5959	0.0031	leaching	0
10A	68.3	12	5868	2.6373	0.0334	1.5825	0.0034	leaching	0
11A	65.7	9	7220	2.5720	0.0277	1.5877	0.0024	leaching	0
12A	64.4	10	6205	2.7176	0.0338	1.5700	0.0025	leaching	0

13A	62.3	14	4525	2.6900	0.0402	1.5621	0.0026	leaching	0
14A	61.4	11	5340	2.6055	0.0423	1.5545	0.0025	leaching	0
15A	63.6	15	4108	2.8777	0.0302	1.5497	0.0025	leaching	0
16A	51.5	24	2139	3.0229	0.0314	1.5371	0.0031	leaching	0
17A	47.4	13	3701	4.0276	0.0550	1.6118	0.0029	leaching	0
18A	33.7	73	461	4.4983	0.0587	1.7524	0.0046	leaching	0
1B	2.99	8253	**0**	1.9935	0.0613	1.3515	0.0185	n/a	
2B	1.40	3969	**0**	2.0389	0.0892	1.3774	0.0165	n/a	
3B	5.17	2130	**2**	1.0123	0.0194	1.4199	0.0092	n/a	
4B	1.68	1943	**1**	1.0188	0.0661	1.3124	0.0214	n/a	
5B	1.98	1569	**1**	1.1727	0.0605	1.2881	0.0255	n/a	
6B	63.2	425	149	2.2930	0.0173	1.6079	0.0025	leaching	0

7B	60.5	7	9139	2.4702	0.0227	1.6044	0.0026	leaching	0
8B	64.9	8	8150	2.3725	0.0156	1.5979	0.0022	leaching	0
9B	66.0	7	9341	2.5172	0.0166	1.5898	0.0026	leaching	0
10B	65.1	4	14649	2.7443	0.0282	1.5931	0.0027	leaching	0
11B	65.2	9	7403	2.7630	0.0358	1.5896	0.0030	leaching	0
12B	64.2	20	3230	2.8130	0.0347	1.5779	0.0027	leaching	0
13B	63.6	18	3464	2.8928	0.0417	1.5635	0.0025	leaching	0
14B	60.9	11	5380	2.9292	0.0334	1.5528	0.0033	leaching	0
15B	59.0	17	3492	3.0892	0.0339	1.5533	0.0026	leaching	0
16B	52.8	25	2129	3.3958	0.0359	1.5440	0.0028	leaching	0
17B	45.9	18	2601	4.7201	0.0491	1.6198	0.0030	leaching	0
18B	26.2	503	52	3.9470	0.0452	1.7250	0.0094	leaching	0
