# Supplementary material for: A near-continuous archaeological record of Pleistocene human occupation at Leang Bulu Bettue, Sulawesi, Indonesia
Source: PLoS One. 2025 Dec 23;20(12):e0337993. doi: 10.1371/journal.pone.0337993 (PMC12725638; doi:10.1371/journal.pone.0337993)
Supplement: S4 Table — No age calculations were carried out for U concentrations of ≤0.5 ppm or U/Th ≤ 300, but not negative (indicated in red). Negative U/Th are due to the average background being higher than the specific measurement. All errors are 2σ. (PDF) [file pone.0337993.s004.pdf]

S4 Table. U-series results on bones and teeth from Leang Bulu Bettue. No age calculations were carried out for U concentrations that were of ≤0.5 ppm or U/Th ≤300 but not negative (indicated in red). Negative U/Th are due to the average background being higher than the specific measurement. All errors are 2σ.

**3608**	U (ppm)	Th (ppb)	U/Th	$^{230}Th/^{238}U$	$^{230}Th/^{238}U$ error	$^{234}U/^{238}U$	$^{234}U/^{238}U$ error	CS Age (ka)	CS Age error (ka)	Diff Age (ka)	Diff Age error (ka)
1	11.2	28	395	0.2585	0.0108	1.0324	0.0059	31.4	1.5	31.5	1.5
2	14.6	13	1151	0.2583	0.0089	1.0470	0.0060	30.9	1.3	30.9	1.3
3	19.0	7	2578	0.1228	0.0039	1.0255	0.0051	13.9	0.5	13.9	0.5
4	15.8	31	513	0.0962	0.0032	1.0082	0.0119	11.0	0.4	11.0	0.4
5	**0.20**	2	**119**	0.1688	0.0612	1.0154	0.0373	n/a			
6	1.07	17	**63**	0.6163	0.0181	1.1182	0.0143	n/a			
7	5.56	18	313	0.5312	0.0136	1.1301	0.0094	68.3	2.6	69.3	2.6

8	11.7	47	**246**	0.2723	0.0059	1.0355	0.0042	n/a	n/a		
9	12.9	42	308	0.3273	0.5643	1.0428	0.2769	41.0	0.8	41.1	0.8
10	16.6	8	2162	0.2176	0.0061	1.0311	0.0040	25.8	0.8	25.9	0.8
11	14.5	15	989	0.2684	0.0083	1.0339	0.0076	32.8	1.2	32.8	1.2
12	12.7	18	686	0.6009	0.0201	1.0895	0.0111	86.3	4.7	87.6	4.8
13	10.5	32	324	1.4655	0.0943	1.1844	0.0207	leaching			
14	13.9	34	408	0.3993	0.0079	1.0528	0.0050	51.8	1.4	52.1	1.4
**Mean Values**											
1-4 3608A Dentine	15.1 ±1.6			0.1736	0.0032	1.0275	0.0051	20.2	0.4	20.2	0.4
9-12	14.7			0.3408	0.0048	1.0474	0.0032	42.8	0.8	43.0	0.8

3608B	±0.8

Dentine

**3609**	U (ppm)	Th (ppb)	U/Th	$^{230}Th/^{238}U$	$^{230}Th/^{238}U$ error	$^{234}U/^{238}U$	$^{234}U/^{238}U$ error	CS Age (ka)	CS Age error (ka)	Diff Age (ka)	Diff Age error (ka)
1	0.90	1	618	0.1782	0.0177	0.9187	0.0198	23.6	2.7	23.5	2.7
2	0.71	0	1945	0.1651	0.0162	0.9190	0.0217	21.7	2.5	21.6	2.4
3	107.1	1	126696	0.2988	0.0029	0.9342	0.0025	42.3	0.5	42.1	0.5
4	100.7	0	333500	0.2884	0.0039	0.9362	0.0018	40.4	0.7	40.2	0.7
5	104.7	0	223567	0.2908	0.0056	0.9356	0.0025	40.8	1.0	40.6	1.0
6	98.5	1	96092	0.3261	0.0035	0.9417	0.0060	46.6	0.7	46.4	0.7
7	77.1	8	9337	0.3565	0.0016	0.9274	0.0024	53.4	0.4	53.0	0.4
8	62.5	1	59237	0.3014	0.0040	0.9176	0.0056	43.8	0.8	43.5	0.8
9	105.2	1	102984	0.3266	0.0023	0.9310	0.0035	47.5	0.5	47.2	0.5

10	89.8	1	153154	0.2802	0.0059	0.9388	0.0036	38.9	1.0	38.7	1.0
11	97.9	1	156490	0.2930	0.0033	0.9353	0.0031	41.2	0.6	41.0	0.6
12	3.82	0	8254	0.2150	0.0079	0.9437	0.0109	28.3	1.3	28.2	1.2
13	79.5	1	144512	0.3302	0.0073	0.9340	0.0078	47.9	1.4	47.6	1.4
14	86.4	1	94451	0.3433	0.0077	0.9284	0.0037	50.8	1.5	50.4	1.5
15	0.90	1	131395	0.3461	0.0095	0.9194	0.0146	52.0	2.2	51.6	2.1
16	0.71	1	31905	0.3619	0.0051	0.9336	0.0035	53.9	1.0	53.6	1.0
17	107.1	1	175817	0.3440	0.0022	0.9310	0.0033	50.7	0.5	50.4	0.5
18	100.7	1	133253	0.3592	0.0018	0.9289	0.0027	53.8	0.4	53.4	0.4

Mean Values

1-2 Enamel	0.80 ±0.09			0.1724	0.0229	0.9188	0.0281	22.8	3.5	22.7	3.4

3-11<br>Dentine	93.7<br>±14.9	0.3077	0.0038	0.9349	0.0036	43.8	0.7	43.6	0.7
13-15<br>Cement	85.2<br>±3.0	0.3402	0.0084	0.9270	0.0043	50.3	1.6	49.9	1.7
17-18<br>Dentine	99.9<br>±4.2	0.3513	0.0026	0.9300	0.0038	52.2	0.6	51.8	0.6

**3610**	U (ppm)	Th (ppb)	U/Th	$^{230}Th/^{238}U$	$^{230}Th/^{238}U$ error	$^{234}U/^{238}U$	$^{234}U/^{238}U$ error	CS Age (ka)	CS Age error (ka)	Diff Age (ka)	Diff Age error (ka)
1	13.9	5	2769	0.1427	0.0027	0.8852	0.0053	19.3	0.4	19.2	0.4
2	12.5	2	5024	0.1490	0.0033	0.8734	0.0041	20.5	0.5	20.4	0.5
3	12.7	1	8750	0.1564	0.0032	0.8779	0.0058	21.5	0.5	21.4	0.5
4	11.2	0	24997	0.1520	0.0036	0.8778	0.0039	20.9	0.6	20.8	0.6
5	11.2	1	19830	0.1474	0.0035	0.8607	0.0105	20.6	0.6	20.5	0.6
6	11.7	1	22261	0.1515	0.0032	0.8678	0.0050	21.1	0.5	20.9	0.5
7	12.1	0	48684	0.1306	0.0026	0.8302	0.0047	18.8	0.4	18.7	0.4
8	11.0	0	26680	0.1011	0.0031	0.8166	0.0057	14.5	0.5	14.4	0.5
9	37.1	0	88246	0.1622	0.0037	0.8514	0.0045	23.2	0.6	23.1	0.6

10	61.5	1	74090	0.1876	0.0026	0.8621	0.0048	27.0	0.5	26.8	0.5
11	67.7	1	61864	0.1956	0.0019	0.8571	0.0026	28.5	0.3	28.3	0.3
12	66.0	1	51770	0.1974	0.0023	0.8552	0.0055	28.9	0.5	28.7	0.4
13	70.4	1	50687	0.2075	0.0029	0.8525	0.0079	30.7	0.6	30.5	0.6
14	71.8	1	57495	0.2017	0.0038	0.8590	0.0095	29.5	0.8	29.2	0.7
15	78.8	2	42922	0.2004	0.0020	0.8538	0.0021	29.4	0.3	29.2	0.3
16	78.9	1	73761	0.2109	0.0019	0.8511	0.0032	31.4	0.4	31.1	0.3
17	78.7	1	78305	0.2096	0.0031	0.8650	0.0093	30.5	0.7	30.3	0.6
18	70.3	1	80409	0.2152	0.0024	0.8546	0.0025	32.0	0.4	31.7	0.4
19	66.6	1	86659	0.1909	0.0032	0.8577	0.0031	27.7	0.5	27.5	0.5
20	56.8	1	74255	0.1996	0.0027	0.8574	0.0046	29.2	0.5	28.9	0.5
21	54.0	0	116717	0.1886	0.0029	0.8563	0.0022	27.4	0.5	27.2	0.5

22	57.4	1	94753	0.2025	0.0020	0.8583	0.0015	29.6	0.4	29.4	0.3
23	54.0	0	124797	0.2072	0.0016	0.8570	0.0029	30.5	0.3	30.2	0.3
24	49.2	1	86066	0.1969	0.0033	0.8532	0.0027	28.9	0.6	28.7	0.6
25	53.7	1	73398	0.1933	0.0029	0.8544	0.0057	28.2	0.5	28.0	0.5
26	50.3	1	61952	0.1981	0.0025	0.8599	0.0018	28.8	0.4	28.6	0.4
27	48.6	1	37536	0.1972	0.0053	0.8655	0.0190	28.4	1.2	28.2	1.1
28	46.2	2	23661	0.1976	0.0036	0.8647	0.0124	28.5	0.8	28.3	0.8
					**Mean Values**						
1-8 Enamel	12.0 ±0.3			0.1417	0.0018	0.8621	0.0027	19.7	0.3	19.6	0.3
9-28 Dentine	60.9 ±2.7			0.1993	0.0007	0.8572	0.0007	29.1	0.1	28.9	0.1

**3611**	U (ppm)	Th (ppb)	U/Th	$^{230}Th/^{238}U$	$^{230}Th/^{238}U$ error	$^{234}U/^{238}U$	$^{234}U/^{238}U$ error	CS Age (ka)	CS Age error (ka)	Diff Age (ka)	Diff Age error (ka)
1	8.48	4564	**2**	1.4772	0.0284	1.1585	0.0048	n/a			
2	11.3	29	390	0.7025	0.0106	1.1410	0.0042	101.6	2.6	104.4	2.8
3	14.4	15	940	0.4529	0.0138	1.0959	0.0061	57.7	2.4	58.2	2.4
4	21.0	10	2051	0.1936	0.0039	1.0377	0.0046	22.5	0.5	22.6	0.5
5	25.3	7	3575	0.1703	0.0026	1.0448	0.0059	19.4	0.3	19.4	0.3
6	23.9	5	5217	0.1267	0.0022	1.0357	0.0058	14.2	0.3	14.2	0.3
7	12.4	3	3654	0.1054	0.0031	1.0337	0.0051	11.7	0.4	11.7	0.4
8	67.9	3	22446	1.6579	0.0318	1.2413	0.0170	leaching			
9	103.9	4	29234	1.5493	0.0147	1.2326	0.0122	leaching			

10	99.2	4	25013	1.4888	0.0146	1.2207	0.0070	leaching				
11	104.6	3	32180	1.5158	0.0238	1.2097	0.0035	leaching				
12	99.7	6	16836	1.6930	0.0119	1.2016	0.0081	leaching				
13	86.3	15	5692	1.7736	0.0165	1.2095	0.0016	leaching				
14	93.1	8	12080	1.5434	0.0140	1.2273	0.0072	leaching				
15	20.4	6	3206	1.3871	0.0201	1.2262	0.0093	leaching				
16	1.32	9	**145**	0.7142	0.0235	1.1981	0.0147	n/a				
17	79.1	6	12378	1.4550	0.0275	1.1889	0.0071	leaching				
18	40.8	7	5544	1.5282	0.0266	1.1816	0.0058	leaching				
19	4.03	7	580	0.4081	0.0083	1.0703	0.0067	52.1	1.4	52.4	1.4	
20	2.20	6	372	0.4632	0.0095	1.1382	0.0108	56.4	1.7	57.1	1.7	
21	1.72	7	**255**	0.6336	0.0190	1.2238	0.0231	n/a				

22	90.3	5	16838	1.3504	0.0156	1.2488	0.0018	635.6	637.2	0.0	0.0
23	109.9	7	16241	1.2673	0.0195	1.2432	0.0101	341.3	40.2	0.0	0.0
24	110.9	11	10374	1.3403	0.0085	1.2265	0.0045	leaching			
25	119.0	14	8350	1.4509	0.0062	1.2255	0.0023	leaching			
**Mean Values**											
2-7 Enamel	18.1 ±2.5			0.2510	0.0018	1.0570	0.0034	29.5	0.3	29.6	0.3
19-20 Enamel	2.65 ±0.70			0.4722	0.0096	1.1223	0.0077	58.9	1.7	59.6	1.8
22-25 Dentine	108 ±6			1.3544	0.0062	1.2351	0.0018	leaching			

**3612**	U (ppm)	Th (ppb)	U/Th	$^{230}Th/^{238}U$	$^{230}Th/^{238}U$ error	$^{234}U/^{238}U$	$^{234}U/^{238}U$ error	CS Age (ka)	CS Age error (ka)	Diff Age (ka)	Diff Age error (ka)
1	0.86	24	**36**	0.2939	0.0186	0.7744	0.0125	n/a			
2	1.24	0	3487	0.1513	0.0088	0.7243	0.0101	26.0	1.8	25.6	1.7
3	4.43	0	11518	0.1972	0.0067	0.7222	0.0052	35.6	1.5	34.8	1.4
4	5.37	0	13950	0.1926	0.0049	0.7087	0.0074	35.5	1.2	34.6	1.1
5	83.4	3	28608	0.2171	0.0024	0.7046	0.0019	41.4	0.6	40.2	0.6
6	141.7	1	149739	0.2171	0.0028	0.7072	0.0025	41.2	0.7	40.1	0.6
7	143.9	1	189472	0.1830	0.0041	0.7071	0.0052	33.4	1.0	32.7	0.9
8	147.0	1	281016	0.2051	0.0036	0.7071	0.0028	38.4	0.9	37.4	0.8
9	144.4	1	157353	0.1856	0.0021	0.7079	0.0037	34.0	0.5	33.2	0.5

10	144.4	1	215749	0.2163	0.0027	0.7070	0.0036	41.0	0.7	39.9	0.7
11	149.5	1	122570	0.2122	0.0023	0.7090	0.0013	39.9	0.5	38.8	0.5
12	147.2	1	142751	0.2250	0.0018	0.7113	0.0025	42.8	0.5	41.5	0.4
13	131.5	3	45216	0.2097	0.0028	0.7080	0.0013	39.4	0.7	38.4	0.6
14	105.1	6	18313	0.2222	0.0021	0.7032	0.0018	42.8	0.5	41.5	0.5
15	90.5	7	12829	0.2215	0.0014	0.7052	0.0015	42.4	0.4	41.2	0.3
16	129.2	13	9636	0.2229	0.0014	0.7075	0.0011	42.6	0.4	41.3	0.3
17	96.7	10	9652	0.2261	0.0026	0.7086	0.0027	43.3	0.7	42.0	0.6
18	103.2	13	8146	0.2242	0.0027	0.7112	0.0014	42.6	0.7	41.3	0.6
19	146.5	12	11989	0.2133	0.0037	0.7089	0.0044	40.2	0.9	39.1	0.9
20	128.6	12	10765	0.2236	0.0039	0.7064	0.0038	42.8	1.0	41.5	0.9
21	128.2	11	11783	0.2251	0.0020	0.7146	0.0037	42.5	0.6	41.3	0.5

22	160.8	11	15224	0.2230	0.0022	0.7146	0.0072	42.0	0.8	40.9	0.7
				**Mean Values**							
2-4 Enamel	3.68 ±1.25			0.1899	0.0057	0.7159	0.0060	34.4	1.3	33.7	1.3
6-22 Dentine	130 ±6			0.2164	0.0007	0.7089	0.0006	40.9	0.2	39.8	0.2

**3613**	U (ppm)	Th (ppb)	U/Th	$^{230}Th/^{238}U$	$^{230}Th/^{238}U$ error	$^{234}U/^{238}U$	$^{234}U/^{238}U$ error	CS Age (ka)	CS Age error (ka)	Diff Age (ka)	Diff Age error (ka)
1	13.9	5	2769	0.1427	0.0027	0.8852	0.0053	19.3	0.4	19.2	0.4
2	12.5	2	5024	0.1490	0.0033	0.8734	0.0041	20.5	0.5	20.4	0.5
3	12.7	1	8750	0.1564	0.0032	0.8779	0.0058	21.5	0.5	21.4	0.5
4	11.2	0	24997	0.1520	0.0036	0.8778	0.0039	20.9	0.6	20.8	0.6
5	11.2	1	19830	0.1474	0.0035	0.8607	0.0105	20.6	0.6	20.5	0.6
6	11.7	1	22261	0.1515	0.0032	0.8678	0.0050	21.1	0.5	20.9	0.5
7	12.1	0	48684	0.1306	0.0026	0.8302	0.0047	18.8	0.4	18.7	0.4
8	11.0	0	26680	0.1011	0.0031	0.8166	0.0057	14.5	0.5	14.4	0.5
9	37.1	0	88246	0.1622	0.0037	0.8514	0.0045	23.2	0.6	23.1	0.6

10	61.5	1	74090	0.1876	0.0026	0.8621	0.0048	27.0	0.5	26.8	0.5
11	67.7	1	61864	0.1956	0.0019	0.8571	0.0026	28.5	0.3	28.3	0.3
12	66.0	1	51770	0.1974	0.0023	0.8552	0.0055	28.9	0.5	28.7	0.4
13	70.4	1	50687	0.2075	0.0029	0.8525	0.0079	30.7	0.6	30.5	0.6
14	71.8	1	57495	0.2017	0.0038	0.8590	0.0095	29.5	0.8	29.2	0.7
15	78.8	2	42922	0.2004	0.0020	0.8538	0.0021	29.4	0.3	29.2	0.3
16	78.9	1	73761	0.2109	0.0019	0.8511	0.0032	31.4	0.4	31.1	0.3
17	78.7	1	78305	0.2096	0.0031	0.8650	0.0093	30.5	0.7	30.3	0.6
18	70.3	1	80409	0.2152	0.0024	0.8546	0.0025	32.0	0.4	31.7	0.4
19	66.6	1	86659	0.1909	0.0032	0.8577	0.0031	27.7	0.5	27.5	0.5
20	56.8	1	74255	0.1996	0.0027	0.8574	0.0046	29.2	0.5	28.9	0.5
21	54.0	0	116717	0.1886	0.0029	0.8563	0.0022	27.4	0.5	27.2	0.5

22	57.4	1	94753	0.2025	0.0020	0.8583	0.0015	29.6	0.4	29.4	0.3
23	54.0	0	124797	0.2072	0.0016	0.8570	0.0029	30.5	0.3	30.2	0.3
24	49.2	1	86066	0.1969	0.0033	0.8532	0.0027	28.9	0.6	28.7	0.6
25	53.7	1	73398	0.1933	0.0029	0.8544	0.0057	28.2	0.5	28.0	0.5
26	50.3	1	61952	0.1981	0.0025	0.8599	0.0018	28.8	0.4	28.6	0.4
27	48.6	1	37536	0.1972	0.0053	0.8655	0.0190	28.4	1.2	28.2	1.1
28	46.2	2	23661	0.1976	0.0036	0.8647	0.0124	28.5	0.8	28.3	0.8
**Mean Values**											
1-8 Enamel	12.0 ±0.3			0.1417	0.0018	0.8621	0.0027	19.7	0.3	19.6	0.3
9-28 Dentine	60.9 ±2.7			0.1993	0.0007	0.8572	0.0007	29.1	0.1	28.9	0.1

**3614**	U (ppm)	Th (ppb)	U/Th	$^{230}Th/^{238}U$	$^{230}Th/^{238}U$ error	$^{234}U/^{238}U$	$^{234}U/^{238}U$ error	CS Age (ka)	CS Age error (ka)	Diff Age (ka)	Diff Age error (ka)
1	**0.21**	2	**95**	0.4067	0.1095	0.9358	0.0730	n/a			
2	0.90	0	1911	0.1341	0.0092	0.9604	0.0162	16.4	1.3	16.4	1.3
3	0.91	1	1580	0.1594	0.0143	0.9752	0.0177	19.5	2.0	19.5	2.0
4	11.4	0	34412	0.1299	0.0047	0.9430	0.0088	16.2	0.7	16.2	0.7
5	96.4	0	249225	0.1404	0.0017	0.9482	0.0042	17.5	0.2	17.5	0.2
6	97.0	0	481908	0.1459	0.0015	0.9527	0.0036	18.2	0.2	18.1	0.2
7	98.6	1	194294	0.1529	0.0025	0.9575	0.0059	19.0	0.4	19.0	0.4
8	106.7	0	651051	0.1546	0.0013	0.9520	0.0033	19.4	0.2	19.3	0.2
9	83.5	4	19359	0.1439	0.0023	0.9518	0.0046	17.9	0.3	17.9	0.3

10	44.8	40	1127	0.1615	0.0021	0.9630	0.0026	20.1	0.3	20.1	0.3
11	31.5	1	33445	0.1543	0.0024	0.9572	0.0036	19.2	0.3	19.2	0.3
12	1.36	0	3082	0.1323	0.0130	0.9525	0.0159	16.3	1.8	16.3	1.8
13	65.0	1	48764	0.1794	0.0020	0.9583	0.0032	22.7	0.3	22.6	0.3
14	56.2	10	5672	0.1723	0.0037	0.9570	0.0066	21.7	0.5	21.7	0.5
15	66.6	1	56169	0.1582	0.0030	0.9516	0.0031	19.9	0.4	19.8	0.4
16	2.84	0	90364	0.1289	0.0062	0.9571	0.0111	15.8	0.9	15.8	0.8
17	1.90	1	3020	0.1193	0.0064	0.9499	0.0116	14.7	0.9	14.6	0.9
18	1.44	0	-16191	0.1406	0.0085	0.9529	0.0145	17.5	1.2	17.4	1.2
19	35.6	0	80474	0.1525	0.0029	0.9599	0.0191	18.9	0.6	18.9	0.6
20	107.5	1	205434	0.1614	0.0020	0.9422	0.0111	20.6	0.4	20.5	0.4
21	119.2	1	83326	0.1607	0.0010	0.9483	0.0021	20.3	0.1	20.3	0.1

22	113.3	1	83458	0.1565	0.0021	0.9479	0.0037	19.7	0.3	19.7	0.3
23	99.2	13	7484	0.1585	0.0025	0.9472	0.0079	20.0	0.4	20.0	0.4
24	64.1	51	1249	0.1653	0.0029	0.9345	0.0117	21.3	0.5	21.3	0.5
25	104.9	1	93920	0.1508	0.0025	0.9505	0.0038	18.9	0.3	18.9	0.3
26	107.2	1	122541	0.1436	0.0023	0.9458	0.0052	18.0	0.3	18.0	0.3
27	116.1	0	393449	0.1582	0.0022	0.9477	0.0071	20.0	0.3	19.9	0.3
28	5.98	0	13111	0.1318	0.0050	0.9261	0.0131	16.8	0.7	16.8	0.7
28	1.82	0	4456	0.1157	0.0110	0.9240	0.0132	14.6	1.5	14.6	1.5
30	**0.47**	1	916	0.1450	0.0388	0.9361	0.0329	n/a			
					**Mean Values**						
2-3<br>Enamel	0.90<br>±0.01			0.1468	0.0131	0.9678	0.0230	18.0	1.8	18.0	1.8

5-11 Dentine	79.8 ±11.1	0.1493	0.0010	0.9536	0.0020	18.6	0.1	18.6	0.1
13-15 Cement	62.6 ±3.2	0.1698	0.0023	0.9555	0.0036	21.4	0.3	21.4	0.3
16-18 Enamel	2.06 ±0.41	0.1287	0.0072	0.9539	0.0128	15.8	1.0	15.8	1.0
20-27 Dentine	104 ±6	0.1565	0.0007	0.9461	0.0015	19.8	0.1	19.7	0.1
28-30 Enamel	2.76 ±1.66	0.1290	0.0058	0.9262	0.0151	16.4	0.8	16.4	0.8

**3615**	U (ppm)	Th (ppb)	U/Th	$^{230}Th/^{238}U$	$^{230}Th/^{238}U$ error	$^{234}U/^{238}U$	$^{234}U/^{238}U$ error	CS Age (ka)	CS Age error (ka)	Diff Age (ka)	Diff Age error (ka)
1	29.9	6	4609	0.5593	0.0062	1.1360	0.0026	72.8	1.2	74.0	1.2
2	31.5	2	15551	0.2323	0.0032	1.0432	0.0063	27.5	0.5	27.5	0.5
3	29.0	2	16032	0.1465	0.0031	1.0176	0.0074	17.0	0.4	17.0	0.4
4	25.4	1	19989	0.1069	0.0022	1.0157	0.0028	12.1	0.3	12.1	0.3
5	23.9	1	19609	0.0820	0.0020	0.9999	0.0081	9.3	0.2	9.3	0.2
6	20.7	2	11907	0.0749	0.0025	1.0114	0.0113	8.4	0.3	8.4	0.3
7	108.5	1	81496	0.8229	0.0090	1.0913	0.0027	148.2	3.5	153.2	3.8
8	106.7	0	237835	0.7285	0.0090	1.0915	0.0018	117.5	2.6	120.2	2.8
9	119.8	0	302879	0.7737	0.0084	1.0816	0.0017	133.8	2.9	137.2	3.0

10	126.1	1	183959	0.8255	0.0081	1.0748	0.0020	154.9	3.4	159.5	3.7
11	118.5	1	155131	0.8297	0.0070	1.0919	0.0017	150.5	2.8	155.8	3.0
12	118.9	1	99494	0.8602	0.0071	1.0955	0.0019	161.3	3.1	167.9	3.4
13	108.9	1	101020	0.9487	0.0050	1.0944	0.0041	206.5	4.2	220.2	4.7
14	107.9	1	108289	0.7584	0.0082	1.0952	0.0033	125.4	2.6	128.8	2.8
15	112.7	1	133119	0.7380	0.0097	1.0961	0.0024	119.2	2.9	122.2	3.1
16	104.9	1	88392	0.6899	0.0084	1.0906	0.0022	107.2	2.2	109.4	2.4
17	99.2	1	75723	0.6628	0.0088	1.0897	0.0026	100.5	2.2	102.3	2.3
18	107.7	1	82097	0.6618	0.0076	1.0833	0.0039	101.4	2.0	103.1	2.1
19	105.9	1	88488	0.6460	0.0083	1.0905	0.0046	96.4	2.1	98.0	2.2
20	103.9	1	112484	0.6485	0.0071	1.0871	0.0033	97.5	1.8	99.1	1.8
21	90.1	1	73921	0.6814	0.0051	1.0866	0.0059	105.7	1.7	107.7	1.7

22	69.0	1	71515	0.7263	0.0057	1.0789	0.0020	119.7	1.7	122.1	1.8
23	2.41	0	9386	0.5341	0.0134	1.0629	0.0105	75.6	3.0	76.2	3.0
24	2.75	0	8631	0.6828	0.0172	1.0504	0.0101	113.2	5.5	114.7	5.6
25	1.88	0	5458	0.7608	0.0164	1.0274	0.0084	145.8	7.4	147.3	7.4
26	1.63	0	8683	0.7486	0.0183	1.0172	0.0113	144.5	8.6	145.4	8.5
27	3.45	1	6195	0.6084	0.0116	1.0700	0.0099	90.7	3.1	91.8	3.1
28	3.34	1	6322	0.5570	0.0148	1.0735	0.0098	79.0	3.3	79.9	3.4
					**Mean Values**						
1-6 Enamel	26.7 ±1.7			0.2151	0.0016	1.0409	0.0021	25.2	0.2	25.3	0.2
7-22 Dentine	107 ±3			0.7546	0.0025	1.0888	0.0008	125.8	0.8	129.0	1.0

23-28 Enamel	2.58 ±0.30	0.6323	0.0095	1.0554	0.0068	98.7	2.6	99.8	2.7

**3616**	U (ppm)	Th (ppb)	U/Th	$^{230}Th/^{238}U$	$^{230}Th/^{238}U$ error	$^{234}U/^{238}U$	$^{234}U/^{238}U$ error	CS Age (ka)	CS Age error (ka)	Diff Age (ka)	Diff Age error (ka)
1	9.52	2569	**4**	0.7316	0.0118	1.1020	0.0075				
2	12.8	13	998	0.4756	0.0703	1.0851	0.0106	62.4	13.1	63.0	13.4
3	17.2	3	5345	0.4305	0.0230	1.0801	0.0075	55.1	3.9	55.5	4.0
4	19.7	3	7575	0.3865	0.0676	1.0717	0.0083	48.6	11.2	48.8	11.4
5	20.3	2	11562	0.2857	0.0041	1.0653	0.0075	34.0	0.6	34.1	0.6
6	19.9	2	10392	0.2164	0.0183	1.0546	0.0111	25.0	2.4	25.1	2.4
7	16.8	2	8607	0.1506	0.0465	1.0363	0.0095	17.1	5.9	17.2	5.9
8	17.2	3	6114	0.1632	0.0553	1.0399	0.0073	18.6	7.1	18.6	7.1
9	3.98	2	2409	0.1356	0.0384	1.0269	0.0084	15.5	4.8	15.5	4.8

10	45.7	2	27936	0.3528	0.0041	1.0503	0.0084	44.5	0.8	44.7	0.8
11	78.0	2	35006	0.2872	0.0026	1.0239	0.0069	35.9	0.5	35.9	0.5
12	74.6	2	37116	0.2568	0.0112	1.0186	0.0109	31.7	1.7	31.7	1.7
13	76.4	2	42644	0.2888	0.0111	1.0247	0.0053	36.1	1.7	36.2	1.7
14	82.7	2	44707	0.3413	0.0344	1.0325	0.0062	43.7	5.5	43.8	5.6
15	83.3	2	46389	0.4253	0.0449	1.0425	0.0228	57.0	8.4	57.2	8.4
16	84.1	2	43736	0.5152	0.0052	1.0439	0.0052	73.9	1.2	74.3	1.2
17	84.7	3	27181	0.4860	0.0507	1.0501	0.0346	67.4	10.7	67.9	10.8
18	71.5	7	10326	0.6191	0.0172	1.0578	0.0052	95.1	4.3	96.1	4.4
19	27.5	429	**64**	0.7148	0.0111	1.0544	0.0093	n/a			
20	62.6	261	**240**	0.6568	0.0074	1.0524	0.0070	n/a			
21	83.5	7	12378	0.5781	0.0037	1.0583	0.0043	85.5	1.0	86.3	1.0

22	83.6	3	26646	0.4818	0.0072	1.0554	0.0078	66.1	1.6	66.6	1.5
23	80.1	2	33154	0.4509	0.0356	1.0545	0.0269	60.6	6.9	60.9	7.0
24	70.8	2	31004	0.3984	0.0046	1.0457	0.0046	52.2	0.8	52.4	0.8
25	81.0	1	60315	0.3798	0.0084	1.0510	0.0056	48.8	1.4	49.0	1.4
26	80.0	2	44253	0.4134	0.0049	1.0455	0.0037	54.7	0.9	55.0	0.9
27	80.7	1	65851	0.4425	0.0034	1.0487	0.0036	59.6	0.7	59.9	0.7
**Mean Values**											
1-9 Enamel	16.7 ±1.3	1.33		0.3261	0.0029	1.0641	0.0052	39.8	0.5	40.0	0.5
10-18 Dentine	75.7 ±4.1	4.05		0.3997	0.0018	1.0378	0.0035	52.9	0.4	53.1	0.4
21-27	80.0	1.63		0.4510	0.0026	1.0515	0.0027	60.8	0.5	61.2	0.5

Dentine	±1.6

**3617-1**	U (ppm)	Th (ppb)	U/Th	$^{230}Th/^{238}U$	$^{230}Th/^{238}U$ error	$^{234}U/^{238}U$	$^{234}U/^{238}U$ error	CS Age (ka)	CS Age error (ka)	Diff Age (ka)	Diff Age error (ka)
1	0.50	197	**3**	0.3034	0.0278	0.7951	0.0226	n/a			
2	**0.26**	0	-2281	0.1792	0.0366	0.7897	0.0602	n/a			
3	**0.22**	0	-423525	0.1700	0.0431	0.7078	0.0400	n/a			
4	0.92	0	-3713	0.1201	0.0137	0.7557	0.0123	19.1	2.5	18.9	2.4
5	0.53	0	-1662	0.1301	0.0256	0.7825	0.0251	20.0	4.5	19.9	4.4
6	1.12	1	1892	0.0583	0.0094	0.7274	0.0126	9.2	1.6	9.1	1.5
7	2.75	0	8868	0.0462	0.0038	0.6945	0.0081	7.6	0.7	7.5	0.6
8	3.13	0	14872	0.0623	0.0051	0.6873	0.0097	10.5	0.9	10.4	0.9
9	2.56	0	8042	0.0619	0.0048	0.7026	0.0076	10.1	0.8	10.1	0.8

10	3.36	1	4822	0.0601	0.0046	0.7045	0.0079	9.8	0.8	9.7	0.8
11	3.80	1	7446	0.0566	0.0032	0.7039	0.0094	9.2	0.6	9.1	0.6
**Mean Values**											
1-11 Enamel	1.86 ±0.44			0.0657	0.0020	0.7071	0.0048	10.7	0.4	10.7	0.4

3617-2	U (ppm)	Th (ppb)	U/Th	$^{230}Th/^{238}U$	$^{230}Th/^{238}U$ error	$^{234}U/^{238}U$	$^{234}U/^{238}U$ error	CS Age (ka)	CS Age error (ka)	Diff Age (ka)	Diff Age error (ka)
1	13.6	1	19179	0.0881	0.0035	0.7467	0.0049	13.8	0.6	13.7	0.6
2	39.6	3	14294	0.0978	0.0029	0.7427	0.0048	15.5	0.5	15.4	0.5
3	105.8	4	25841	0.1143	0.0018	0.7616	0.0035	17.9	0.3	17.8	0.3
4	139.2	12	11291	0.1235	0.0007	0.7688	0.0034	19.3	0.2	19.1	0.2
5	116.2	3	35860	0.1182	0.0027	0.7791	0.0118	18.1	0.6	18.0	0.5
6	103.0	0	213304	0.1163	0.0014	0.7750	0.0026	17.9	0.2	17.8	0.2
7	116.1	2	51866	0.1177	0.0019	0.7730	0.0021	18.2	0.3	18.0	0.3
8	123.4	4	28357	0.1142	0.0012	0.7694	0.0051	17.7	0.2	17.5	0.2
9	121.3	3	45459	0.1020	0.0013	0.7725	0.0027	15.6	0.2	15.5	0.2

10	121.0	2	64788	0.1064	0.0017	0.7644	0.0020	16.5	0.3	16.4	0.3
11	127.3	2	84039	0.1121	0.0012	0.7691	0.0014	17.3	0.2	17.2	0.2
12	128.4	2	67456	0.1122	0.0013	0.7689	0.0019	17.4	0.2	17.2	0.2
13	128.5	2	77160	0.1121	0.0014	0.7712	0.0015	17.3	0.2	17.2	0.2
14	134.5	2	54909	0.1126	0.0011	0.7660	0.0016	17.5	0.2	17.4	0.2
15	110.1	1	128096	0.1025	0.0023	0.7646	0.0055	15.8	0.4	15.7	0.4
16	129.3	1	120386	0.1090	0.0009	0.7683	0.0025	16.8	0.2	16.7	0.2
17	123.0	1	121371	0.1074	0.0011	0.7744	0.0045	16.4	0.2	16.3	0.2
18	119.2	1	137492	0.1111	0.0013	0.7699	0.0017	17.1	0.2	17.0	0.2
19	116.8	1	130893	0.1091	0.0015	0.7699	0.0039	16.8	0.3	16.7	0.3
20	92.1	1	109923	0.1091	0.0019	0.7667	0.0051	16.9	0.3	16.8	0.3
21	112.6	1	87665	0.1074	0.0011	0.7721	0.0014	16.5	0.2	16.4	0.2

22	101.3	2	52747	0.1064	0.0014	0.7703	0.0037	16.4	0.3	16.2	0.2
23	121.5	3	44653	0.1080	0.0023	0.7751	0.0099	16.5	0.5	16.4	0.4
24	123.0	3	42957	0.1052	0.0016	0.7673	0.0050	16.2	0.3	16.1	0.3
25	135.1	3	47456	0.1103	0.0012	0.7749	0.0031	16.9	0.2	16.8	0.2
26	123.5	1	100582	0.1108	0.0011	0.7699	0.0018	17.1	0.2	17.0	0.2
27	111.2	2	52176	0.1094	0.0014	0.7695	0.0018	16.9	0.2	16.7	0.2
28	116.4	3	43961	0.1115	0.0014	0.7744	0.0031	17.1	0.3	17.0	0.2
28	118.1	7	16056	0.1103	0.0009	0.7705	0.0020	17.0	0.2	16.9	0.2
30	130.6	4	33818	0.1105	0.0014	0.7857	0.0068	16.7	0.3	16.5	0.3
**Mean Values**											
1-30 Dentine	113 ±5			0.1105	0.0003	0.7704	0.0005	17.0	0.1	16.9	0.1
