# Supplementary material for: A near-continuous archaeological record of Pleistocene human occupation at Leang Bulu Bettue, Sulawesi, Indonesia
Source: PLoS One. 2025 Dec 23;20(12):e0337993. doi: 10.1371/journal.pone.0337993 (PMC12725638; doi:10.1371/journal.pone.0337993)
Supplement: S5 Table — (PDF) [file pone.0337993.s005.pdf]

S5 Table. Summary of average laser ablation U-series ages for faunal remains from Leang Bulu Bettue.

**Sample**	**Layer**	**Depth (cm)**	**U (ppm)**	**^{230}Th/^{238}U**	**^{234}U/^{238}U**	**CS Age (ka)**	**Diff Age (ka)**
LBB3 (1A-14A)	**4a**	**156**	70.0±4.4	0.1229±0.0022	0.9284±0.0021	15.5±0.3	15.5±0.3
LBB3 (1B-14B)	**4a**	**156**	68.4±5.7	0.1256±0.0036	0.9272±0.0023	15.9±0.5	15.9±0.5
3617-2 (1-30)	**4b**	**170**	113±5	0.1105±0.0003	0.7704±0.0005	17.0±0.1	16.9±0.1
LBB15 (1A-8A)	**4b/5**	**174.5**	72.0±5.0	0.0754±0.0021	0.98865±0.0020	8.65±0.26	8.65±0.26
LBB15 (1B-9B)	**4b/5**	**174.5**	74.0±5.6	0.0783±0.0017	0.9903±0.0019	8.99±0.20	8.99±0.20
LBB15 (1C-8C)	**4b/5**	**174.5**	76.1±7.6	0.0792±0.0026	0.9896±0.0019	9.09±0.31	9.09±0.31
3612 (6-22)	**4c**	**191**		0.2164±0.0007	0.7089±0.0006	40.9±0.2	39.8±0.2

3610 (9-29)	**4b**	**193**	60.9±2.7	0.1993±0.0007	0.8572±0.0007	29.1±0.1	28.9±0.1
3614 (5-11)	**5**	**202.5**	79.8±11.1	0.1493±0.0010	0.9536±0.0020	18.6±0.1	18.6±0.1
3614 (13-15)	**5**	**202.5**	62.6±3.2	0.1698±0.0023	0.9555±0.0036	21.4±0.3	21.4±0.3
3614 (20-27)	**5**	**202.5**	104±6	0.1565±0.0007	0.9461±0.0015	19.8±0.1	19.7±0.1
LBB7 (3A-7A)	**5**	**210**	36.6±26.3	0.7224±0.0243	1.0951±0.0250	117±8	119±9
LBB7 (3B-7B)	**5**	**210**	36.0±21.9	0.7251±0.0233	1.0967±0.0245	116±7	119±9
LBB7 (2C-6C)	**5**	**210**	36.9±25.7	0.7631±0.0260	1.0869±0.0240	130±7	133±7
3613 (9-28)	**5**	**224**	60.9±2.7	0.1993±0.0007	0.8572±0.0007	29.1±0.1	29.9±0.1

3609 (3-11)	**5**	**232**	93.7±14.9	0.3077±0.0038	0.9349±0.0036	43.8±0.7	43.6±0.7
3609 (13-15)	**5**	**232**	85.2±3.0	0.3402±0.0084	0.9270±0.0043	50.3±1.6	49.9±1.7
3609 (17-18)	**5**	**232**	100±4	0.3513±0.0026	0.9300±0.0038	52.2±0.6	51.8±0.6
3611 (22-25)	**8**	**392**	108±6	1.3544±0.0062	1.2351±0.0018	leaching	
LBB19 (6A-18A)	**8**	**407**	61.2±10.8	1.4569±0.0906	1.3442±0.0128	leaching	leaching
LBB19 (6B-18B)	**8**	**407**	58.2±11.2	1.5119±0.0953	1.3449±0.0111	leaching	leaching
3615 (7-22)	**8**	**407**	107±3	0.7546±0.0025	1.0888±0.0008	125±1	129±1

3608 (1-4)	**8**	**409.5**	15.1±1.6	0.1736±0.0032	1.0275±0.0051	20.2±0.4	20.2±0.4
3608 (9-12)	**8**	**409.5**	14.7±0.8	0.3408±0.0048	1.0474±0.0032	42.8±0.8	43.0±0.8
LBB10 (2A-10A)	**8**	**410-420**	102±13	1.1102±0.0195	1.2967±0.0043	185±8	214±13
LBB10 (2B-10B)	**8**	**410-420**	97.3±7.5	1.1039±0.0145	1.2931±0.0031	183±6	211±8
LBB10 (1C-10C)	**8**	**410-420**	89.0±31.0	1.1656±0.0350	1.2919±0.0028	221±20	266±86
LBB13 (3A-6A)	**10**	**500-510**	94.2±10.6	0.1507±0.0046	1.0595±0.0018	16.7±0.6	16.8±0.5
LBB13 (3B-6B)	**10**	**500-510**	90.5±12.0	0.1620±0.0060	1.0601±0.0018	18.1±0.7	18.1±0.7
LBB13 (1C-5C)	**10**	**500-510**	84.8±11.5	0.1658±0.0030	1.0603±0.0019	18.5±0.4	18.6±0.4
3616 (10-18)	**10**	**507-510**	75.7±4.1	0.3997±0.0018	1.0378±0.0035	52.9±0.4	53.1±0.4

3616 (21-27)	**10**	**507-510**	80.0±1.6	0.4510±0.0026	1.0515±0.0027	60.8±0.5	61.2±0.5
LBB18 (6A-12A)	**10d**	**540-550**	12.9±0.8	0.3225±0.0270	1.0082±0.0086	42.2±4.0	42.2±4.0
LBB18 (5B-11B)	**10d**	**540-550**	14.6±1.2	0.3061±0.0261	0.9942±0.0105	40.3±3.8	40.3±3.8
LBB18 (5C-11C)	**10d**	**540-550**	18.8±7.0	0.5520±0.0673	1.0850±0.0201	79.4±13.1	81.1±14.0
LBB17 (3A-10A)	**11**	**613**	98.7±8.0	0.5377±0.0125	1.1190±0.0024	70.5±2.2	71.5±2.3
LBB17 (2B-8B)	**11**	**613**	94.2±9.1	0.5150±0.0169	1.1196±0.0027	66.5±2.9	67.4±3.0
LBB17 (3C-8C)	**11**	**613**	100±3	0.5416±0.0131	1.1192±0.0021	71.2±2.3	72.2±2.4
