# Supplementary material for: A near-continuous archaeological record of Pleistocene human occupation at Leang Bulu Bettue, Sulawesi, Indonesia
Source: PLoS One. 2025 Dec 23;20(12):e0337993. doi: 10.1371/journal.pone.0337993 (PMC12725638; doi:10.1371/journal.pone.0337993)
Supplement: S6 Table — Equivalent dose (De) estimation model, cutoff value used for outlier rejection, number of micro-aliquots accepted during data analysis (after outlier rejection) out of the total measured, overdispersion values of the Ln/Tn or De distribution (depending on age model used) before (ODtotal) and after outlier rejection (ODnMAD), expected scatter of a well-bleached De distribution (σb), De and ages are provided for each sample. (PDF) [file pone.0337993.s006.pdf]

S6 Table. D_e estimation details for age determination. Equivalent dose (D_e) estimation model, cutoff value used for outlier rejection, number of micro-aliquots accepted during data analysis (after outlier rejection) out of the total measured, overdispersion values of the L_n/T_n or D_e distribution (depending on age model used) before (OD_{total}) and after outlier rejection (OD_{nMAD}), expected scatter of a well-bleached D_e distribution (σ_b), D_e and ages are provided for each sample.

**Sample code**	**Layer**	**Model**	**nMAD cutoff**	**Accepted micro-aliquots**	**OD_{total}** (%)	**OD_{nMAD}** (%)	**σ_b**	**D_e** (Gy)	**Age[a] (ka)**	
									**Fading uncorrected**	**Fading corrected**
LBB-II	5	nMAD MAM D_e	1.5	34 / 2600	120 ± 13	54 ± 9	0.35	159.1 ± 32.6	29.4 ± 6.6	**33.5 ± 9.1**
LBB15-OSL3	5	nMAD MAM D_e	1.5	92 / 3800	90 ± 7	56 ± 6	0.35	185.3 ± 24.1	35.0 ± 5.0	**40.1 ± 6.2**
LBB15-OSL2	9b	nMAD CAM L_n-/T_n	2.0	77 / 1800	27 ± 3	20 ± 2	-	661.2 ± 54.7	163.1 ± 14.9	**188.5 ± 19.9**
LBB15-OSL0	10c	nMAD CAM L_n-/T_n	2.0	79 / 500	73 ± 6	30 ± 3	-	585.6 ± 63.3	134.4 ± 15.7	**155.1 ± 22.8**

[a] Ages are obtained by subtracting a residual dose (10.4 ± 0.7 Gy) from the D_e and dividing by the corresponding dose rate.
