# Supplementary material for: A near-continuous archaeological record of Pleistocene human occupation at Leang Bulu Bettue, Sulawesi, Indonesia
Source: PLoS One. 2025 Dec 23;20(12):e0337993. doi: 10.1371/journal.pone.0337993 (PMC12725638; doi:10.1371/journal.pone.0337993)
Supplement: S7 Table — Burial depths and stratigraphic layers, grain sizes, proportion of water per dry sample mass used for age calculation and field water contents in parentheses as well as dosimetry components are provided for each sample. Bulk radionuclide concentrations were determined through thick-source alpha counting coupled with beta-counting. The total dose rate (Ḋtotal) is the sum of gamma (Ḋγ), external beta (Ḋβ external), internal beta (Ḋβ internal) and cosmic (Ḋcosmic) dose rates. (PDF) [file pone.0337993.s007.pdf]

S7 Table. Sampling and D_e estimation details. Burial depths and stratigraphic layers, grain sizes, proportion of water per dry sample mass used for age calculation and field water contents in parentheses as well as dosimetry components are provided for each sample. Bulk radionuclide concentrations were determined through thick-source alpha counting coupled with beta-counting. The total dose rate ($\dot{D}_{total}$) is the sum of gamma ($\dot{D}_{\gamma}$), external beta ($\dot{D}_{\beta\ external}$), internal beta ($\dot{D}_{\beta\ internal}$) and cosmic ($\dot{D}_{cosmic}$) dose rates.

**Sample code**	**Layer**	**Depth** (cm)	**Grain size** (µm)	**Water** (%)	**Dose rate ($\dot{D}$)** (Gy/ka)				
					$\dot{D}_{\gamma}$	$\dot{D}_{\beta\ external}$	$\dot{D}_{\beta\ internal}$	$\dot{D}_{cosmic}$	$\dot{D}_{total}$
LBB-II	5	197	90–180	28 (30)	2.11 ± 0.14 [b]	2.24 ± 0.21	0.55 ± 0.07	0.13 ± 0.01	5.04 ± 0.26
LBB15-OSL3	5	210	90–125	28 (25)	2.06 ± 0.12 [a]	2.41 ± 0.13	0.45 ± 0.06	0.13 ± 0.01	5.04 ± 0.19
LBB15-OSL2	9b	460	90–125	39 (39)	1.68 ± 0.09 [a]	1.81 ± 0.09	0.45 ± 0.06	0.10 ± 0.01	4.03 ± 0.14
LBB15-OSL0	10c	540	90–125	25 (25)	1.35 ± 0.08 [a]	2.42 ± 0.14	0.45 ± 0.06	0.10 ± 0.01	4.31 ± 0.17

[a] Gamma dose rate determined by *in situ* gamma spectrometry

[b] Gamma dose rate determined from bulk radionuclide concentrations (1.4 ± 0.2% K; 12.7 ± 0.4 ppm U; 21.9 ± 2.4 ppm Th)
