# Supplementary material for: A near-continuous archaeological record of Pleistocene human occupation at Leang Bulu Bettue, Sulawesi, Indonesia
Source: PLoS One. 2025 Dec 23;20(12):e0337993. doi: 10.1371/journal.pone.0337993 (PMC12725638; doi:10.1371/journal.pone.0337993)
Supplement: S8 Table — Number of Identified Specimens (NISP) and Minimum Number of Individuals (MNI). (PDF) [file pone.0337993.s008.pdf]

S8 Table. Number of Identified Specimens (NISP) and Minimum Number of Individuals (MNI).

	**ALL SQUARES: NISP/MNI**				
**Taxon**	**Phase IV layer 1**	**Phase III layers 2-3**	**Phase II layer 4 sequence**	**Phase I layer 5**	**Phase I layers 6-13**
Crocodilidae	0	3 / 1	1 / 1	0	0
Anoa (*Bubalus* sp.)	0	4 / 2	6 / 1	33 / 4	58 / 7
Bos	2 / 1	0	0	0	0
Cervidae	6 / 2	0	0	0	0
Proboscidea	0	0	0	4 / 1	17 / 2
Palaeoloxodon	0	0	0	3 / 1	12 / 2
Stegodon	0	0	0	0	5 / 2
Suidae	2 / 1	0	135 / 7	3 / 1	12 / 1
Celebochoerus	0	0	0	0	2 / 1
Babyrousa	0	0	1 / 1	2 / 1	0
Sus	2 / 1	0	134 / 6	1 / 1	10 / 1
Ailurops ursinus	0	0	48 / 5	10 / 1	4 / 1
Strigokuskus celebensis	0	0	10 / 2	2 / 1	0
Macaca sp.	0	1 / 1	3 / 2	1 / 1	4 / 1
Tarsius sp.	0	0	3 / 1	0	0
Macrochiroptera	0	0	1 / 1	2 / 1	0
Muridae	0	0	344 / n.a.	32 / n.a.	0
Sciuridae	0	0	1	0	0
Soricidae	0	0	1	0	0
