# Supplementary material for: A near-continuous archaeological record of Pleistocene human occupation at Leang Bulu Bettue, Sulawesi, Indonesia
Source: PLoS One. 2025 Dec 23;20(12):e0337993. doi: 10.1371/journal.pone.0337993 (PMC12725638; doi:10.1371/journal.pone.0337993)
Supplement: S9 Table — List of proboscidean fossils from Leang Bulu Bettue. (PDF) [file pone.0337993.s009.pdf]

S9 Table. List of proboscidean fossils from Leang Bulu Bettue.

Square/Layer/spit	Year-Coll. nr. / sieve	Taxon	Element	Remarks
B1: Layer 4b-5, spit 20	2014-523	Proboscidea	thoracic? vertebra fragment	
B2: Layer 5, spit 18	2014-386	Proboscidea	tarsal, heavily damaged	
A2: Layer 5, spit 19	2013-649	Proboscidea	left metacarpal-IV fragment	
-A1: Layer 5, spit 24	2014 sieve	Proboscidea	tooth enamel fragment	
A2: Layer 8, spit 39	2014 sieve	*Stegodon* spec	molar plate fragment	heavily water-rolled
-A2: Layer 8, spit 40	2014 sieve	Proboscidea	4 small enamel fragments	
-A2: Layer 8, spit 40	2014 sieve	*Stegodon* spec.	1 molar ridge	encrusted
B2: Layer 8, spit 40	2014 sieve	Proboscidea	6 tiny enamel fragments	
A2: Layer 8, spit 41	2013 sieve	Proboscidea	1 enamel fragment	rolled
-A1: Layer 8, spit 41	2014-1408	Proboscidea	small molar plate fragment	weathered
-A1: Layer 8, spit 41	2014-1415	Proboscidea	molar plate fragment	glossy polish on dentine
A2: Layer 8, spit 41	2014 sieve	Proboscidea	small enamel fragment	
B2: Layer 8, spit 42	2014-610	*Palaeoloxodon* cf. *namadicus*	left lower M1	same individual as below
B1: Layer 8, spit 42	2014-630	*Palaeoloxodon* cf. *namadicus*	right mandible with M1 fragment	same individual as above

-A1: Layer 10, spit 49	2015 sieve	*Stegodon* spec.	molar ridge fragment	heavily water-rolled
A1: Layer 10, spit 51	2013-sieve?	*Stegodon* spec.	molar ridge fragment	heavily water-rolled
-A1: Layer 10, spit 48-51	2015 baulk	Proboscidea	enamel fragment	
A2: Layer 10b, spit 54-55	2013-nr?	*Palaeoloxodon* cf. *namadicus*	left mandible fragment with dp3	+ 2 isolated ridges from alveole
A2: Layer 10b, spit 58	2013 sieve	Proboscidea	enamel fragment	heavily water-rolled
-A1: Layer 10e, spit 59	2015 sieve	Proboscidea	enamel fragment	heavily water-rolled
-A2: Layer 13, spit 78	2015-178	Proboscidea	small tusk cementum fragment	
-A2: Layer 13, spit 78	2015 sieve	Proboscidea	enamel fragment	heavily water-rolled
-A2: Layer 13, spit 78	2015 sieve	Proboscidea	enamel fragment	heavily water-rolled
-A2: Layer 13, spit 79	2015 sieve	*Stegodon*? spec	enamel fragments	heavily water-rolled
-C1: Layer 10	2019 plotted	Proboscidea	Mandible fragment	
-H1/1: Layer 8, spit 47, No. 4569	2023 plotted	Proboscidea	Tusk fragment	Heavily water-rolled
-G1: Layer 10b, spit 35. No. 5419	2023 plotted	Proboscidea	Rib fragment	
I1/1: Layer 6b2. Spit 41, No. 4470	2023 plotted	Proboscidea	Enamel fragment	
I1/1: Layer 6, spit 31, No. 4383	2023 plotted	Proboscidea	Enamel fragment	

-G1: Layer 10, spit 33, No. 5462	2023 plotted	Proboscidea	Enamel fragment
-G1: Layer 7, spit 31, No. 5374	2023 plotted	Proboscidea	Enamel fragment
-H2: Layer 6b, spit 28, No. 442	2023 plotted	Proboscidea	Enamel fragment
-H2: Layer 6b, spit 28, No. 444	2023 plotted	Proboscidea	Enamel fragment
-I1: Layer 6b3/7, spit 36, No. 4266	2023 plotted	Proboscidea	Enamel fragment
-H1: Layer 12, spit 69, No. 4948	2023 plotted	Proboscidea	Enamel fragment
