# Supplementary material for: A near-continuous archaeological record of Pleistocene human occupation at Leang Bulu Bettue, Sulawesi, Indonesia
Source: PLoS One. 2025 Dec 23;20(12):e0337993. doi: 10.1371/journal.pone.0337993 (PMC12725638; doi:10.1371/journal.pone.0337993)
Supplement: S10 Table — (PDF) [file pone.0337993.s010.pdf]

S10 Table. Size ranges of lower molars of *Elephas maximus* and other Elephantini species.

	Tooth	number of lamellae	length (mm)	width (mm)	Height	H/W index	LF	ET (mm)
E. maximus	dP3	6-9	32-62	26-39				
	dP4	11-14	97-155	32-58			8-10	1.3
	M1	12-17	150-200	45-69	81	170	7-8	1.2-1.8
	M2	16-21	180-265	58-73	115-140	182-207	5-8	
	M3	21-29	240-390	70-85	115-178	153-217	5-7	2.7-3.2
E. hysudrindicus	dP3	7	65.5	30.9	34	111	12.1	1.2
	dP4							
	M1							
	M2			65-69	145		5-6	
	M3	18-21	297-403	70-87	115-150	154-201	4.6-7	3.1-4.5
P. namadicus	dP3	5-7	55-73	18-26			9.4-11	1.0-1.1
	dP4	9-11	120-129	33-40	75	187.5	7.8-9.4	1.0-1.3
	M1	9-11	131-179	31-57	115	230	6.5-8.1	1.5-2.0
	M2	9-14	185-292	53-99	106-173	160-234	4.3-6.8	1.6-3.0

	**M3**	13-18	233-339	50-88	123.5-166	145-302	4.4-6.8	1.8-3.4
E. hysudricus	**dP3**	6-8	67-85.6	37-43	35.6-45	115.7	7.9-11.9	1.0-1.1
	**dP4**	9	112.3-138.7	47.6-59.2	58.2-75	116.7-126.8	6.9-8.6	1.3-2.3
	**M1**	9-10	121-180	52-78	81.8-111.2	129.6-142.7	5.1-8.2	1.0-2.7
	**M2**	10	190-211	71.8-76	93.1-97.3	128.1-135.4	4.9-5.3	2.7-3.1
	**M3**	12-17	254-354	74-107	104.1-159	124.3-166.7	4.1-6.5	1.8-3.5
