# Supplementary material for: A near-continuous archaeological record of Pleistocene human occupation at Leang Bulu Bettue, Sulawesi, Indonesia
Source: PLoS One. 2025 Dec 23;20(12):e0337993. doi: 10.1371/journal.pone.0337993 (PMC12725638; doi:10.1371/journal.pone.0337993)
Supplement: S11 Table — (PDF) [file pone.0337993.s011.pdf]

S11 Table. Measurements of the Leang Bulu Bettue mandible fragments and *Elephas maximus.*

**Specimen**	**M18 (mm)**	**M6 (mm)**	**M4 (mm)**	**Remarks**
LBB 2014-613	109.4	97e (estimated)	160	M1 in use (7 plates worn)
LBB 2013-?	±42.3	±53	-	neonate, layer 10b, quadrant A2
MGB; Sumatra?	110	113	-	*E. maximus*; recent; M1 in use (5 plates worn)
Cijerah, West Java	167	147	240	*E. maximus*; Late Pleistocene; M3 in use
