# Supplementary figures and images for: A near-continuous archaeological record of Pleistocene human occupation at Leang Bulu Bettue, Sulawesi, Indonesia
Source: PLoS One. 2025 Dec 23;20(12):e0337993. doi: 10.1371/journal.pone.0337993 (PMC12725638; doi:10.1371/journal.pone.0337993)

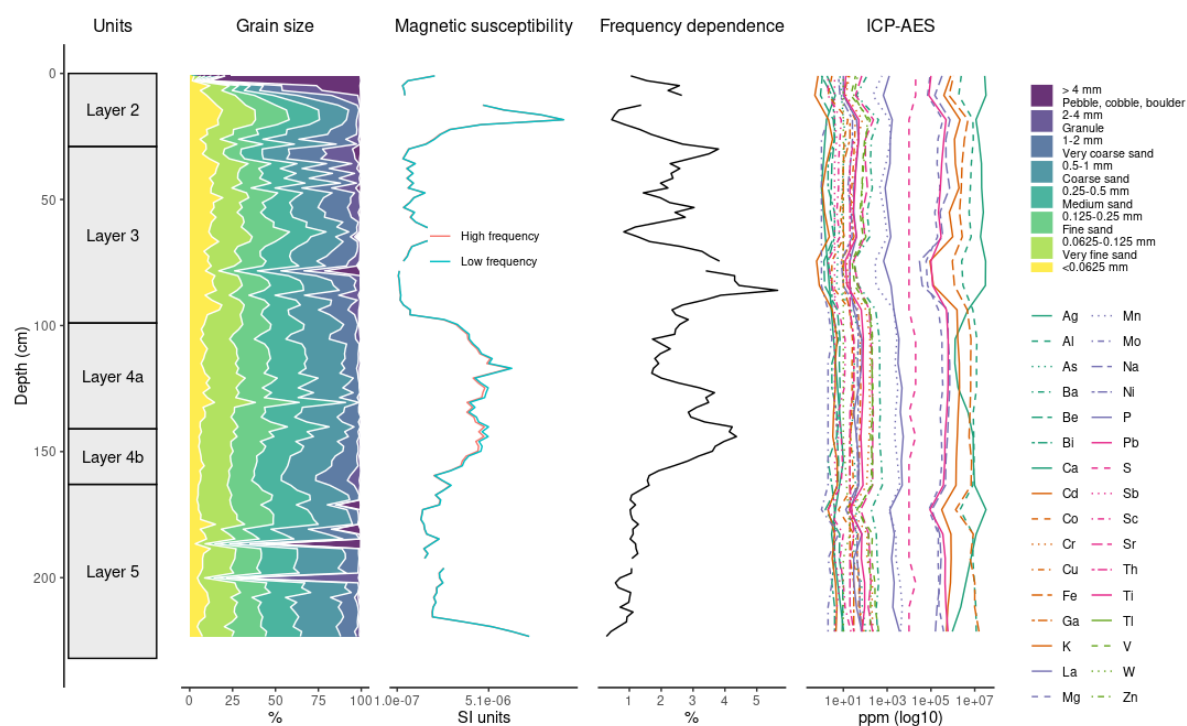

**S1 Fig.** Grainsize and ICP-AES results from section -C2.

Supplement: S1 Fig — (PDF) [file pone.0337993.s012.pdf]

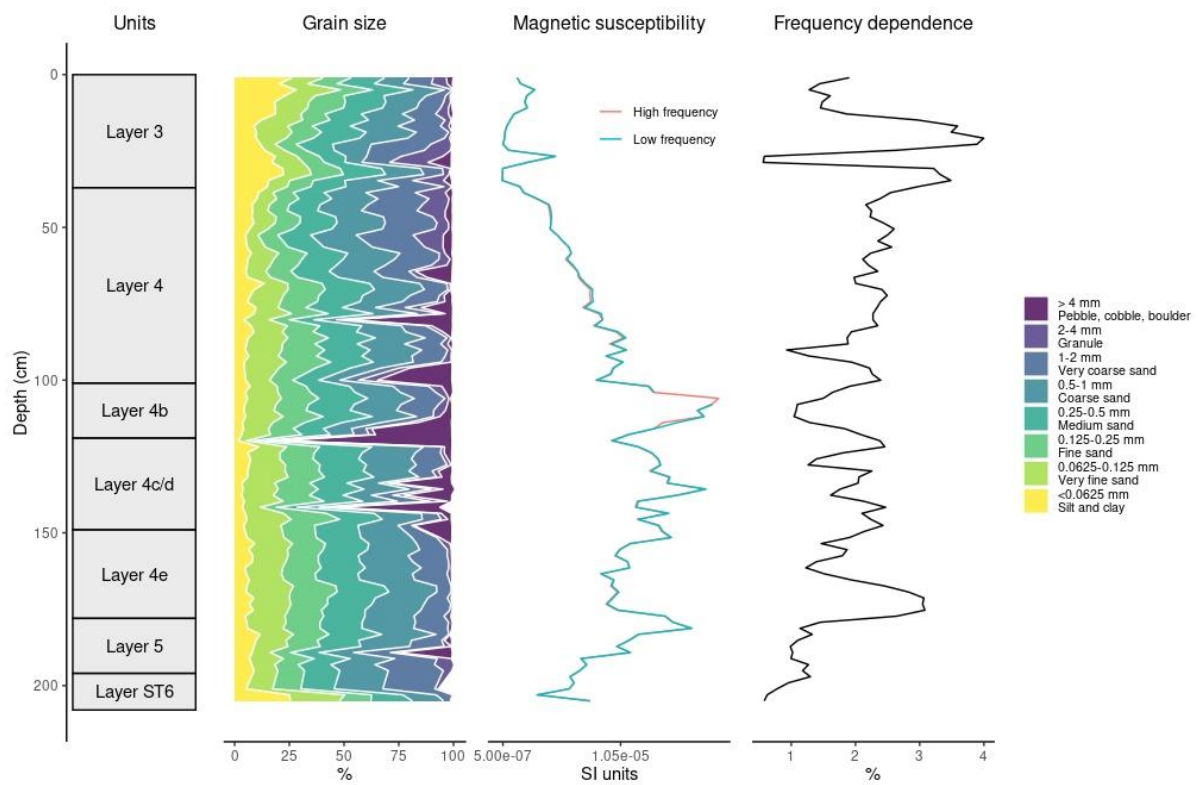

**S2 Fig.** Grainsize results from section -H2.

Supplement: S2 Fig — (PDF) [file pone.0337993.s013.pdf]

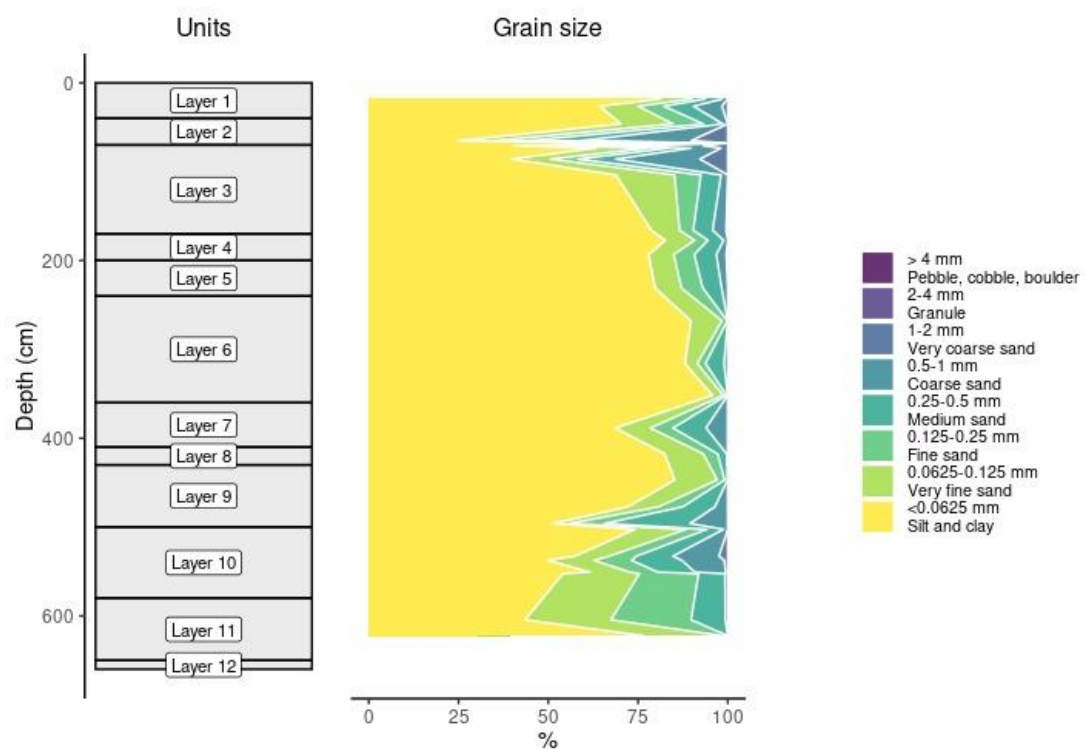

**S3 Fig.** Grainsize results from section A1.

Supplement: S3 Fig — (PDF) [file pone.0337993.s014.pdf]
