# Supplementary material for: A near-continuous archaeological record of Pleistocene human occupation at Leang Bulu Bettue, Sulawesi, Indonesia
Source: PLoS One. 2025 Dec 23;20(12):e0337993. doi: 10.1371/journal.pone.0337993 (PMC12725638; doi:10.1371/journal.pone.0337993)
Supplement: S4 Fig — Two age models were used: (A) a central age model (CAM), and (B) a minimum age model (MAM) using σb = 0.35. Outliers were removed prior to either age models using nMAD cutoff values of (A) 1.5 or (B) 2.0. The radial plots are centred on the nMAD CAM and the grey bands are centred on the modelled values (nMAD CAM or nMAD MAM). OD refers to the overdispersion value. (PDF) [file pone.0337993.s015.pdf]

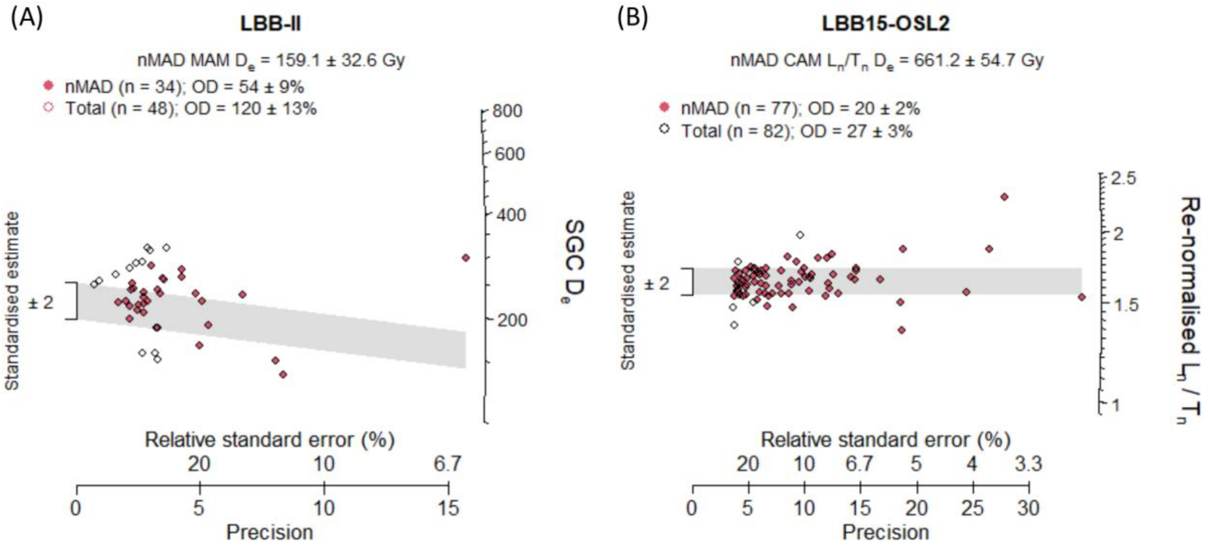

S4 Fig. Examples of distributions of micro-aliquot (A) SGC D_e or (B) re-normalised L_n/T_n with details of the age models used for dating. Two age models were used: (A) a central age model (CAM), and (B) a minimum age model (MAM) using $\sigma_b = 0.35$. Outliers were removed prior to either age models using nMAD cutoff values of (A) 1.5 or (B) 2.0. The radial plots are centred on the nMAD CAM and the grey bands are centred on the modelled values (nMAD CAM or nMAD MAM). OD refers to the overdispersion value.
