# Supplementary material for: A near-continuous archaeological record of Pleistocene human occupation at Leang Bulu Bettue, Sulawesi, Indonesia
Source: PLoS One. 2025 Dec 23;20(12):e0337993. doi: 10.1371/journal.pone.0337993 (PMC12725638; doi:10.1371/journal.pone.0337993)
Supplement: Attachment — Submitted filename: Burhan et al. Response to reviewers.docx [file pone.0337993.s016.docx]

**PONE-D-24-41184**

**Pleistocene human occupation at Leang Bulu Bettue in Sulawesi, Indonesia**

**PLOS ONE**

**Response to Reviewers**

**Editorial comments**

This is clearly a very important site with significant implications. However, Plos One is not the place to publish a site report.

**REPLY:** Respectfully, this is inconsistent with our prior experience publishing in the journal. The authors have already published at least six articles with PLOS One that are essentially “site reports” (e.g., <https://journals.plos.org/plosone/article?id=10.1371/journal.pone.0226628> and <https://journals.plos.org/plosone/article?id=10.1371/journal.pone.0193025>). These two papers alone have >11,000 views combined, showing that such reports are read by more than a small specialist audience. We believe that such papers are valuable contributions as they provide all of the primary research data required to understand a site without the reader having to piece together the story from multiple papers. Notably, with our prior *PLOS One* “site report” papers, all of this data is found in a single open access article that is freely available online (rather than in an expensive academic monograph), so it can be more widely disseminated and thus of greater scientific value. While the present article is longer and more detailed than the others we have published previously, it reports the results of a much larger and more comprehensive program of field research making it a powerful keystone reference for all future Pleistocene archaeological work in the region. We also note that none of the reviewers suggested that the content was inappropriate for PLOS One.

I suggest that you split the ms, and create a Supplementary File to place all the detailed descriptions that result from the excavation.  In the main text, please focus on the important implications of the site, within the broad context of the prehistory of Sulawesi and Wallacea.

**REPLY:** In the revised manuscript we have now moved large sections of descriptive detail to the Supplementary Information section. In total, we've cut about 9000 words from the main text, including removing 8 pages worth of text from the lengthy Discussion/Conclusion, so the paper is now much more tightly focused on the implications of the site in its regional context. We hope you’ll agree that the paper has been substantially revised and reorganised and in the process the overall word count of the main text has been reduced significantly.

Two of the reviewers have provided detailed comments on particular aspects of the analyses; one on bone taphonomy, and the other on the lithics analysis and the dating.  You will need to complete the full revision of these, before resubmission.

**REPLY:** We have addressed these concerns in the revised manuscript; please see below.

In sum, this submission has the potential to make a very significant contribution but it needs to be substantially revised and reorganised.  Some of the detailed description of the site needs to be placed into a Supplementary file and the key findings summarised in the main ms, while several of the analyses need to be developed and more detailed information provided.

**REPLY:** We have followed these suggestions, thank you.

This reorganisation will ensure that the key points are highlighted and accessible to a wider audience rather than as it is now where the level of detail in some places, is likely to be of particular interest only to a a small specialist audience, something that is not appropriate for PLOS One.

**REVIEWER COMMENTS**

**Reviewer #1:**

I am not familiar with archaeology in Indonesia; however, I consider that the authors provide a sufficiently detailed theoretical framework to understand the results presented for the site of Leang Bulu Bettue in Sulawesi. Nonetheless, I recommend incorporating a more detailed parallel discussion of the Eurasian context within the chronological framework of the study. This addition would enable readers to situate the findings within a broader and more meaningful comparative context, particularly for scientists who may not be familiar with archaeology and human evolution in Indonesia.

**REPLY:** A discussion of the Middle to Late Pleistocene Eurasian record is not relevant to understanding the implications of Leang Bulu Bettue in its regional context in Southeast Asia and the wider Australasian region, including Australia and New Guinea. We disagree with the suggestion that findings from SE Asia-Australasia are not “meaningful” unless they are situated in relation to findings in Eurasia. Sulawesi is a Wallacean island permanently cut off from mainland Asia, and it’s greatest implications are for understanding the eastward migration of multiple species of hominins, meaning that the context of Australasia is more relevant. The context of Australia and Southeast Asia is discussed in this paper.

I find the methods section to be overly general, primarily focusing on how the excavations and documentation of the findings were conducted. However, there is a lack of specific, reference-based information regarding the analysis of the recovered remains. In the case of the faunal remains, it is only stated that the bones were examined for cut marks or other indications of butchery, rounding caused by water transport, and polish as potential indicators of use-wear. This aspect necessitates further elaboration in the supplementary materials. Various types of cut marks and different degrees of polish are crucial for making not only biostratinomic but also fossil-diagenetic interpretations. Additionally, there is no mention of quantification indices such as MNI, MNE, NISP, etc.

**REPLY:** This paper is an overview report of a large and complex site. As is common practice in the field, detailed descriptions of analytical methods are/will be available in existing and upcoming specialist reports. Including such detail here would make an already lengthy MS unrealistically long, as noted above. Nevertheless, given the importance of the cut marks we have addressed this comment by adding more details on theiranalysis and including a table of NISP/MNI data in Supplementary Information as recommended.

A critical issue that must be addressed is that the manuscript contains assertions lacking referential support. For example, the statement: “Transverse microstriations run oblique and perpendicular across one cut mark; these relate to a secondary event, possibly trampling or other taphonomic activities” (lines 277-278) should be substantiated by references, as the varying orientations of the marks do not necessarily correlate with secondary or post-depositional processes. Such statements should be carefully reviewed and corrected throughout the manuscript.

**REPLY:** We have revised the manuscript to address these issues; thank you.

Lastly, while the authors provide a comprehensive discussion of the data reported by layers and present their hypotheses, there is insufficient comparison with other sites in a structured and detailed manner. The article is predominantly descriptive and consequently excessively lengthy. I understand that the journal does not impose word limits; however, the text does not conform to the format of a scientific article, as it resembles an extensive report on a site contextualized within the archaeological landscape of the region. I recommend that the authors make an effort to synthesize the content or transfer more general sections (not essential to the article’s objectives) to the supplementary materials. This concern is also evident in the abstract. According to the journal’s guidelines, this section must not exceed 300 words; therefore, the authors should rectify this as well.

**REPLY:** As noted above, we have followed this suggestion to move much of the descriptive detail to supplementary information and have organised the main body to closer resemble a scientific article as requested. We have also reduced the Abstract to about 300 words.

**Reviewer #2:**

Sulawesi is one of the most interesting islands in Wallacea on account of its high degree of faunal endism, its floral diversity, and its deep past. In recent years this has included the discovery of some of the world’s oldest rock art dated at ca. 50 ka-ago, and an archaeological record extending back beyond 100 ka. Australian evidence indicates that our species arrived there perhaps as early as 65 ka-ago and certainly by 50-55 ka. It is also indicated on Borneo ca. 48 ka. and on Sumatra at ca, 60-70 ka. Our species therefore likely arrived on Sulawesi between 60 and 50 ka and replaced the earlier inhabitants.

Leang Bulu Bettue (LBB) is the only site on Sulawesi so far discovered that contains a sequence that extends from the late Middle Pleistocene to after 40 ka. Unfortunately the sequence is not continuous, but it does nonetheless expand our knowledge of the lithic assemblages produced by the earlier, pre-sapiens inhabitants as well as those that made the cave art ca. 50 ka. It is thus an important addition to our current knowledge of the colonisation of Wallacea.

The excavation report is extremely detailed and thorough, and covers all aspects of the excavation. This was clearly conducted at a very high standard. I cannot fault the authors for their level of detail, and it is to the credit of PLoS if it is prepared to publish such a full account. The discussion also places LBB within the current context of debate over the initial, pre-sapiens settlement of Wallacea and its later colonisation by H. sapiens. There are clearly a set of complex changes between 50 and 40 ka that can only be resolved by future exploration. What LBB does is to indicate the next set of priorities, in particular, those that occurred in Marine Isotope Stage 4 (80-60 ka) and in MIS 3 between 50 and 40 ka.

I have no criticisms and recommend publishing the paper in its present form. My only recommendation is that the paragraph on permits, permissions and access (lines 298-309) should be placed at the end of the text before the acknowledgements.

**REPLY:** These comments are much appreciated. There are no issues for us to address.

**Reviewer #3:**

This paper is well written with sufficient archaeological data of the excavation and analysis mainly on lithic and faunal materials as well as various dating. The archaeological site reported and discussed in this article should be very important site in Sulawesi as well as Wallacea or Island Southeast Asian region, thus this paper contains many of significant new data and discussion and should be published but with some minor revisions.

**REPLY:** We appreciate these comments; thank you.

For some minor changes, (1) I recommend to add more references related to the lithic and use-wear analysis data in Wallacea region. For example, the authors describe about this topic in page 30 (Line680-687), but they only referred the past studies in southern Sulawesi, Flores and Timor, but ignored some significant studies on these topics in northern Sulawesi and the Philippines islands. Also some recent studies in Timor and southern Wallacea are not cited. I show some related papers in below for references, and the authors may add some more related and important studies and data for this topic as well as in their discussion.

**REPLY:** We appreciate this, but the use-wear of the LBB assemblage is only mentioned very briefly in our paper (as it is a work in progress) and thus it would not be appropriate for us to cite these other specialised studies.

Another part the authors need to do some minor changes in the discussion part. In page 63, the authors refers another old site in Sulawesi, Goa Topogaro 2 for their discussion, and write as "The Late Pleistocene lithic technology and other cultural evidence from the earliest levels at Goa Topogaro 2 has not yet been described in detail in the published literature". However, they only refer a single paper about this site, and neglect other papers of this site. There are some published papers to report and discuss some details of lithic and faunal materials from this site, while most of materials discussed are younger than 30 ka mainly. Yet, I recommend to check some related papers to add some discussion here. I also list some related papers in below.

**REPLY:** Thank you; we have now cited the relevant studies. We have deleted the sentence: "The Late Pleistocene lithic technology and other cultural evidence from the earliest levels at Goa Topogaro 2 has not yet been described in detail in the published literature".

In general view of this paper and its discussion, the paper tend to focus mainly in southern Sulawesi part. Since the site locates in southern Sulawesi, it should be OK but in same time, the authors may be not so much keen to other regions in Wallacea, and I recommend to add some more related data and discussion in other regions at least in Wallacea (including the Philippines) to make stronger the discussion of this paper. Then, this paper should be more attractive to much wider readers.

**REPLY:** Thank you; we have now cited some of these studies, although not all; the others are superfluous to our (more focused) paper, a point we made above.

The Fuentes et al., 2021 paper does not seem to exist in JASR or the published literature.

(Some related references)

Fuentes, R., R. Ono, H.O. Sofian, N. Aziz, T. Miranda, and A. Pawlik

2021 Detecting the arrival of anatomically modern humans through use-wear analysis: results from Topogaro, Central Sulawesi from 30 thousand years ago. Journal of Archaeological Science : Report.

Fuentes, R., R. Ono, J. Carlos, C. Kerfant, Sriwigati, T. Miranda, N. Aziz, H. O. Sofian, and A. Pawlik.

2020 Stuck within notches: direct evidence of plant processing during the Last Glacial Maximum in North Sulawesi　Journal of Archaeological Science : Report 30, 102207

Fuentes, R., R. Ono, N. Nakajima, H. Nishizawa, J. Siswanto, N. Aziz, Sriwigati, H.O. Sofian, T. Miranda, A.F. Pawlik.

2019 Technological and behavioural complexity in expedient industries: the importance of use-wear analysis for understanding flake assemblages.　Journal of Archaeological Science 112.105031.

Ono, R, Fuentes, R., Noel, A., Sofian, H.O., Sriwigati, Aziz, N., and Pawlik, A

2021a. Development of bone and lithic technologies by anatomically modern humans during the late Pleistocene to Holocene in Sulawesi and Wallacea. Quaternary International 596: 124-143.

Ono, R., Fuentes, R., Pawlik, A.F., Sofian, H.O., Sriwigati, Aziz, N., Alamsyah, N., and Yoneda, M.

2020a. Island migration and foraging behaviour by anatomical modern humans during the late Pleistocene to Holocene in Wallacea: New evidence from Central Sulawesi, Indonesia. Quaternary International 554:90-106.

Pawlik, A. Technology, adaptation, and mobility in maritime environments in the Philippines from the Late Pleistocene to Early/Mid-Holocene. Quaternary International. 2021; 596: 109-123.

Xhauflair, H., N. Revel, T. J. Vitales, J. R. Callado, D. Tandang, C. Gaillard, H. Forestier, E. Dizon, and A. Pawlik

2017 What Plants might Potentially have been Used in the Forests of Prehistoric Southeast Asia? An Insight from the Resources Used Nowadays by Local Communities in the Forested Highlands of Palawan Island. Quaternary International 448: 169–189.

**Reviewer #4:**

This is a well-written, detailed, and encompassing research paper and I congratulate the authors to this multidisciplinary study of an important archaeological site for the region.

**REPLY:** Thank you for this comment, which is most gratifying.

I see only two major issues, one concerning the limited and somewhat elusive presentation of the lithic material, and the other concerning the chronology and the U-series and luminescence dating. Here, I suggest that the authors revise and enhance the respective parts and make them more comprehensible. The following are the specific comments and suggestions:

Line 118: The date and its archaeological context of Madjedbebe in northern Australia has been challenged by several authors, including members of the first excavation. It also hasn’t been verified by similarly old sites along the potential migration routes, yet. While U-series and OSL seem to deliver older dates compared to AMS, the currently oldest secure timeline for Wallacea, including this site, is after 50ka. This would warrant a brief, critical discussion on chronology. And since specialists in radiometric dating are among the authors, perhaps combine this with a discussion why the AMS dates across Island Southeast Asia are in general considerably younger than the U-Series or OSL dates? Also see below.

**REPLY:** The situation presented by the reviewer of younger AMS radiocarbon-based ages relative to U-series and optical ages across Island Southeast Asia is not true for LBB. In layer 4a, the optical ages of two samples are slightly younger than the AMS 14C ages. In layers 4b-e, the optical ages of three samples fall nicely between overlying U-series (24.6–26.0 ka) and underlying AMS 14C ages (covering a range between 25.5 and 27.9 cal ka BP). The other layers lack associated radiocarbon age controls for comparison. In general, the secure age of presence of modern humans in Wallacea is getting older as new investigations are published. The apparent disparity between older ages at Madjedbebe than in Wallacea is, therefore, likely not an issue of chronology, but rather of site preservation or of access to the research infrastructure needed to find, excavate and date archaeological sites. We also highlight that obtaining radiocarbon ages older than ~50 ka is technically challenging (e.g., Higham et al., 2015) and that even using the most refined methods, any minute contamination from modern carbon will significantly skew the resulting age towards younger dates.

Regarding concerns surrounding the Madjedbebe site we have added the following caveat: “…but *** for critical discussions rejecting this date”. A more extensive scrutiny of early human occupation dates seems beyond the aims of the present report as it would not significantly affect the results of this paper.

Higham, T. (2011) ‘European Middle and Upper Palaeolithic radiocarbon dates are often older than they look: problems with previous dates and some remedies’, Antiquity 85, pp. 235–249. <https://doi.org/10.1017/S0003598X00067570>.

144: "reducing dramatically in body size within": Is there really a sufficient fossil base for such an assumption? How was it “dramatic”?

**REPLY:** We have deleted “dramatically”.

146: “based on the most plausible dispersal mechanism — accidental rafting on tsunami debris“. How is this plausible and not wild speculation? What is the scientific basis and archaeological/historical evidence for this, such as historic records of tsunami survivors washed up alive on the shore of another island? This also implies that the authors assume that intentional sea crossing was not within the behavioral capacity of these hominins (whereas successfully hunting large game was), or less likely than surviving a tsunami and migrating to the next island on a piece of driftwood. I would suggest simply writing that it is yet unknown how they reached the islands and that various possibilities have been proposed, with a few references.

**REPLY:** We have done as suggested. Thank you.

165: Could Talepu also be possibly younger, considering the large difference between the ESR and OSL dates? I am just wondering whether 4.9m sediment deposits might not take that much time for “high-energy fluvial gravel deposits” to build up. Is there a more secure timeline for the extinction of Stegodon and Celebochoerus that may support the chronology? I remember that Gert van den Bergh proposed a possible extinction after 200ka.

**REPLY:** The Talepu dating reported in the 2016 *Nature* paper is sound. Both the U-series age and the optical age of ~200 ka for T4 are minimum ages, so, based on currently available data, the true age could be even older, not younger. For the optical age, half the sub-samples had signals at saturation (see Extended Data Figure 9 in van den Bergh et al., 2016b), meaning they are beyond the dating limit of the technique. Note that the optical age and U-series ages for layer T4 are in agreement; T2 only has optical ages. On the second point, as yet there is no secure timeline for the extinction of *Stegodon* and *Celebochoerus* on Sulawesi. This early hominin presence is further backed up by the recent publication of artefacts securely dated to 1 million years old on Sulawesi, at the Calio site.

171: Genomic research: For comparison and regional context, it might be useful to look into the publications by Larena et al. 2021 for the Philippines as neighboring region.

**REPLY:** We have now cited this paper.

590: “clay mineral”, can this be more specific?

**REPLY:** We have now clarified this; thank you.

652 ff: Rephrase. I don’t think that lithic technology was focused on chert reduction, rather tool manufacture. Chert reduction is just a method. Considering the extensive size of this paper, the reader certainly won’t mind learning more about the Phase II assemblage than a reference to the dissertation of one of the authors. Particularly, as it is repetitively described as “technologically complex chert-based lithic toolkit”. The illustrations provided in the figures don’t really highlight such complexity, nor does the generic functional description, so it is difficult to understand where the complexity lies. Also, how does the Phase II lithic assemblage compare with similarly old artefacts from Topogaro 2 in Central Sulawesi, technologically and in terms of identified wear traces and activities? There are several recent papers by Fuentes et al. 2021 and Ono et al. 2021, 2023 available that describe the lithic toolkit in form and function (as well as the bone tools).

**REPLY**: We agree, and the descriptions describing the lithic technology has been edited by lithic specialists MM and YP (coauthors). We also refer the reader to prior publications that describe the lithic artefact technologies more critically in greater detail.

A systematic specialist usewear study has not yet been conducted at LBB so this aspect cannot accurately be compared to other sites.

656f: I don’t really see any sign of an “exceptionally well-controlled anvil-supported bipolar percussion” in Figure 7C-E. The irregular shape of the flakes and negatives suggest otherwise. I am not really convinced that the artefacts are a result of bipolar reduction, and I would like it if the authors could elaborate. The scarring could also be use-related damage and those artefacts could have as well been employed as chisels which would probably result in similar edge wear patterns (see the pièces esquillées debate). Same is the case for the pebble Fig 7f identified as anvil. From a cursory examination of its appearance, it seems rather small and light for an anvil (why not just using speleothem on the cave floor as anvils?), suggesting instead a use as hammer and/or chisel. Its visible wear traces are more consistent with impact scars on pebble and cobbles used as hammers (e.g. from the Mindoro sites, see Pawlik & Piper 2019). An experimental functional/traceological study and discussion not just of the cores but also the resulting flakes would be necessary to be certain. Identifying these would require refitting studies, so the whole process can become quite complex. In general, considering how often the concept of bipolar reduction is now being used for lithic assemblages in ISEA, there is a remarkable paucity of positively identified anvils.

**REPLY:** We do not agree with this reviewer that the Leang Bulu Bettue technology does not contain exceptionally well-controlled bipolar reduction, but perhaps this is a matter of differing theoretical backgrounds influencing what we see in the lithic record. The bipolar cores versus wedges debate is a complicated one with a long history in archaeology. In French Bordesian systematics in the 1960s and earlier, bipolar cores were almost automatically assumed to be wedges. Then an American (Hayden) suggested that some of these could in fact be cores (bipolar cores). Australian Peter White agreed, arguing that’s exactly what they are ethnographically, in Papua New Guinea particularly. The French and other European-trained lithic analysts tended to ignore these New World-Antipodean arguments, and the ethnographic literature more generally, and continued with their traditional interpretation that these objects were almost always wedges. This is still largely the prevailing view in Europe. In actual fact, both things are probably going on; but given the ethnographic literature (and the almost complete lack of bipolar cores as wedges in this literature), we think it’s more parsimonious to call them bipolar until such time as they are proved otherwise.

The criteria used to define the artefacts as bipolar are in line with those used throughout the region (including neighbouring Australia). We note that the anvils are only presented as ‘possible anvils’ – however, they would be perfectly large enough for such a task so we see no reason to discredit this possibility. Please see Perston et al. 2022 for a more detailed discussion, including a discussion of the multiple potential anvils recovered at the site.

659f: The processing of ochre and silica-rich plants is an interesting observation. It would be good if the authors can elaborate on this analysis, despite it being currently unpublished.

**REPLY:** A detailed study of the ochre residues is underway; we are not ready to publish yet.

690: Do the authors have an explanation why no charcoal was retrieved, despite the intensive use of the cave?

**REPLY:** The taphonomic conditions of the Maros Pleistocene sites clearly do not favour charcoal preservation.

724: Was only one 14C date obtained for Phase II? Would it be possible to date more shells from different layers? How about tooth enamel? Same for Phase I where several teeth with enamel are reported. It might at least provide additional support for an age >50ka.

**REPLY:** Multiple 14C dates for shell from Phase II deposits are reported in previous papers which we cite in this study. We discussed the possibility of 14C-dating of tooth enamel from the site with the leading expert in this method, Rachel Wood (ANU); she informed us that the method is still experimental and thus far is only appropriate for Holocene samples.

956 ff: It is difficult to follow the parts on the U-series and OSL dating in this chapter and the SI. For instance, Layer 5 is dated to 40-52ka. How can this date be confirmed by the luminescence dates when they range between 24-46ka? The minimum dates of 40ka and 24ka, respectively, are 16,000 years apart. In the following, always the older limits are used, so at 968f: “The U-series-dated bovid tooth from layer 5 provides an in-sequence minimum age of 51.2 ka for the lower part of this stratum”. Shouldn’t it say 40-51.2ka?

**REPLY:** The 40-52 ka date the reviewer is alluding to for layer 5 is stated in the text as being the U-series derived age range for layer 5 (l. 958-960). At the end of the paragraph (after introducing the optical ages) we give the final ages for Layer 5: "a maximum age of between 40 to 46.3 ka for the mid-to-upper part of layer 5" (l. 968f) and "a minimum age of 51.2 ka for the lower part of this stratum" (l. 970). Additionally, the reviewer suggests replacing "51.2 ka" with "40-51.2 ka" in line 970. However, the 40 ka U-series age is from the mid-to-upper part of layer 5, not the lower part for which we are providing a date in this sentence, as stated. In general, we find that an age of "~40 ka", as used in the final discussion (e.g., l. 1486), accurately summarizes the available chronological data obtained for the transition from Phase I to Phase II, taking into consideration the uncertainties associated with each estimate: optical ages of 33.5±9.1 and 40.1±6.2 as well as U-series ages of 39.8±0.2 ka and 51.8±0.6 ka. In sum, we have reported the dating accurately. The minimum U-series age of 51.2 ka for the bovid tooth from layer 5 is indeed in-sequence with (i.e., older than) dates from overlying strata.

In general, the age ranges appear quite wide which also raises questions about the reliability of the U-series and luminescence dating.

**REPLY:** We have not tried to hide the challenges associated with dating the Pleistocene deposits at this site, especially the ‘deep deposits’. We have worked carefully over the past decade to resolve these problems using the dating methods available to us, including spending years developing a site-specific approach to optical dating of weakly-luminescent feldspar grains. We have now published this method which we innovated for the Leang Bulu Bettue site in a December 2024 paper in *Quaternary Geochronology* (https://www.sciencedirect.com/science/article/pii/S1871101424001420?via%3Dihub)

975ff: This continues for Layers 9-11: Two pIRIR ages between 132-204ka (another wide range for two connecting layers) are apparently supported by U-series dates from the fauna of around 40-81ka. How so? They seem quite different and far apart to me, and do seem to contradict the U-series dates.

**REPLY:** The U-series (> 40-81 ka) and optical ages (132-204 ka) are not in contradiction because the U-series are only minimum ages, as stated, e.g., in lines 985-987, meaning any age higher than 40 ka is in agreement with the U-series age. The large uncertainty is represented in our interpretations: "... the lowest stone artefact excavated thus far (layer 10a) dates to between 132.3–208.4 ka" (l. 997-998).

987ff: Here, other faunal samples from Layers 8-11 that were used for U-series dating are mentioned as ranging between 17-200ka. Why were they considered to be reworked from older deposits and excluded from the analysis, but not the dates from the molars from the same deposits mentioned in the previous paragraph?

**REPLY:** We explained this in the manuscript. These samples were excluded because of their distinct ^234^U/^238^U ratios, while others have clearly experienced recent U-uptake (i.e., overprinting).

1041: It is pointed out here that the layers 6-13 were formed by fluvial processes and their contents were reworked. It appears that reworking is generally a significant issue for the accumulation of the cave deposits and the faunal elements that were used for dating.

**REPLY:** The depositional regimes inside tropical limestone karst caves are complex and episodes of reworking/erosion-and-reinfilling are found in virtually all sites we know of.

I am not saying that the dates are wrong, but the sections on Chronology and dating require further elaboration and more plausible arguments as to why some dates have been accepted as reliable and others coming from the same contexts rejected. As they stand, they leave many questions unanswered.

**REPLY:** We disagree, as per our explanations above.

1325: Is the “perforator” shown in Figure 24? Why is it called that, does it show signs of use traces consistent with perforating action?

**REPLY:** We concur that the label is problematic as it implies use, however it is in line with common terminology used to describe a flake with two unifacially retouched lateral margins that converge to form a point (artefact is now shown in fig. 21H). We have now added some qualifiers to clarify this point.

1348ff: For such simple pebble/cobble assemblages, the question is always how to distinguish between artefact and geofact? Particularly when the raw material is local and if there is considerable reworking potentially causing post-depositional modifications. How was this taken into account in the analysis? Especially the limestone cores shown in Figure 26 could be natural products.

**REPLY:** One of the world’s most experienced lithic analysts (Prof. Mark Moore) identified and analysed these artefacts; they are clearly distinguishable from natural stones.

1608ff: It is also problematic that the oldest known rock art sites contain no archaeological evidence of human occupation, since currently the earliest known cave settlements in Sulawesi and Wallacea date to just over 40ka and almost 10000 years younger than the earliest rock art in the region. Another possibility is therefore that the published rock art dates are simply too old. Not just for this paper but in general, I wish there was more critical and contextual discussion of early dates, whether for rock art, fossils, or human occupation in the region.

**REPLY:** We believe the published dates for the rock art of the region are secure, as we have no reason to doubt the multiple peer-reviewed *Nature* publications. It is beyond the scope of this paper to become a rebuttal of the rock art dates, though if this paper allows for future work to explore the issue more deeply then we will welcome its role in providing data for facilitating robust scientific debates.

1640: “…no early coastal sites with which to test this hypothesis…”. What about Topogaro 2, the authors mention the site earlier. Topogaro may also be of interest for this paper from a geographical perspective: It is located on Central Sulawesi’s east coast and across the island from Maros and Leang Bule Bettue. Do the authors consider a potential connection between the two sites? Fauna and lithic assemblages are not that different from LBB Phase II.

**REPLY:** Now cited and discussed in more detail; see above.

1683f: “…the lack of evidence for ochre processing and use in the uppermost layers of Phase I (especially layer 5) is difficult to explain”. Why would that matter, and can this be explained simply as a result of reworking? Since ochre residues were identified on lithic artefacts.

**REPLY:** The meaning of this comment is unclear.

References:

Fuentes, R. et al., 2021. Inferring human activities from the Late Pleistocene to Holocene in Topogaro 2, Central Sulawesi through use-wear analysis. Journal of Archaeological Science: Reports 37: 102905. https://doi.org/10.1016/j.jasrep.2021.102905.

Larena, M. et al., 2021, Philippine Ayta possess the highest level of Denisovan ancestry in the world. Current Biology 31, 1–12.

Larena, M. et al. 2021, Multiple migrations to the Philippines during the last 50,000 years. PNAS 118(13), e2026132118.

Ono, R. et al., 2021. Development of bone and lithic technologies by anatomically modern humans during the late Pleistocene to Holocene in Sulawesi and Wallacea. Quaternary International, https://doi.org/10.1016/j.quaint.2020.12.045.

Ono, R. et al., 2023. The Goa Topogaro Complex: Human Migration and Mortuary Practice in Sulawesi during the Late Pleistocene and Holocene. L’Anthropologie, 103155.

Pawlik, A. and Piper, P.J., 2019: The Philippines from c. 14000 – 4000 cal BP in regional context. Cambridge Archaeological Journal, 29(1): 1-22.

**JOURNAL REQUIREMENTS (EDITORIAL COMMENTS)**

1. Please ensure that your manuscript meets PLOS ONE's style requirements, including those for file naming. The PLOS ONE style templates can be found at

https://journals.plos.org/plosone/s/file?id=wjVg/PLOSOne_formatting_sample_main_body.pdf and

https://journals.plos.org/plosone/s/file?id=ba62/PLOSOne_formatting_sample_title_authors_affiliations.pdf

**REPLY:** Done.

2. We note that the grant information you provided in the ‘Funding Information’ and ‘Financial Disclosure’ sections do not match.

When you resubmit, please ensure that you provide the correct grant numbers for the awards you received for your study in the ‘Funding Information’ section.

**REPLY:** Done.

3. Thank you for stating in your Funding Statement:

"Australian Research Council grants DE130101560 and FT160100119 awarded to A.B. and DE160100703 awarded to I.M., with additional support provided by the Wenner Gren Foundation (Post-Ph.D. grant) and Griffith University."

Please provide an amended statement that declares *all* the funding or sources of support (whether external or internal to your organization) received during this study, as detailed online in our guide for authors at http://journals.plos.org/plosone/s/submit-now. Please also include the statement “There was no additional external funding received for this study.” in your updated Funding Statement.

Please include your amended Funding Statement within your cover letter. We will change the online submission form on your behalf.

**REPLY:** Done.

4. Thank you for stating the following in the Acknowledgments Section of your manuscript:

"The project was funded by Australian Research Council grants DE130101560 and FT160100119 awarded to A.B., with additional support provided by the Wenner Gren Foundation (Post-Ph.D. grant) and Griffith University. The Indonesian fieldwork was authorised by Badan Riset dan Inovasi Nasional (BRIN). For facilitating this research, we thank former Puslit Arkenas (now BRIN) director I.M. Geria and current director H. Jogaswara, as well as the director of Makassar’s Balai Pelestarian Kebudayaan (BPK), L. Aksa. I.M. was supported by Australian Research Council grant DE160100703. Fieldworkers included: D. Susanti, R. Salempang, H. Arsyad, Muhtar, Sungkar, R. Ali, L. Lantik, Asri, H. Lahab, O. Amrullah, Idrus, M. Husain, Busran, and A. Agus. For advice and assistance, we thank I. Glover, J. Joordens, W. Roebroeks, C. Little, D. Bulbeck, P. Piper, X. Zhihong, A. Abdul, F. Petchey, U. Pietrzak, L. Kinsley, J. Hellstrom, P. Bajo, S. Wroe, M. Young and R. Klaebe."

We note that you have provided funding information that is currently declared in your Funding Statement. However, funding information should not appear in the Acknowledgments section or other areas of your manuscript. We will only publish funding information present in the Funding Statement section of the online submission form.

Please remove any funding-related text from the manuscript and let us know how you would like to update your Funding Statement. Currently, your Funding Statement reads as follows:

"Australian Research Council grants DE130101560 and FT160100119 awarded to A.B. and DE160100703 awarded to I.M., with additional support provided by the Wenner Gren Foundation (Post-Ph.D. grant) and Griffith University."

Please include your amended statements within your cover letter; we will change the online submission form on your behalf."

**REPLY:** Done.

5. Please provide a complete Data Availability Statement in the submission form, ensuring you include all necessary access information or a reason for why you are unable to make your data freely accessible. If your research concerns only data provided within your submission, please write "All data are in the manuscript and/or supporting information files" as your Data Availability Statement.

**REPLY:** Done.

6. Please note that your Data Availability Statement is currently missing [Archaeological assemblages excavated from Leang Bulu Bettue, which form the basis of the current study, are under the permanent curation of Indonesian authorities at Badan Riset dan Inovasi Nasional (BRIN) at Jakarta, Indonesia. The data are not from a third party. Requests to access collections for study, including databases and catalogs of finds, should be directed in the first instance to the directors of the Pusat Riset Arkeologi Lingkungan, Maritim, dan Budaya Berkelanjutan, BRIN.]. If your manuscript is accepted for publication, you will be asked to provide these details on a very short timeline. We therefore suggest that you provide this information now, though we will not hold up the peer review process if you are unable.

**REPLY:** Done.

7. When completing the data availability statement of the submission form, you indicated that you will make your data available on acceptance. We strongly recommend all authors decide on a data sharing plan before acceptance, as the process can be lengthy and hold up publication timelines. Please note that, though access restrictions are acceptable now, your entire data will need to be made freely accessible if your manuscript is accepted for publication. This policy applies to all data except where public deposition would breach compliance with the protocol approved by your research ethics board. If you are unable to adhere to our open data policy, please kindly revise your statement to explain your reasoning and we will seek the editor's input on an exemption. Please be assured that, once you have provided your new statement, the assessment of your exemption will not hold up the peer review process.

**REPLY:** Done.

8. We note that you have referenced (Moore MW. Preliminary report on the stone artefact aequence at Leang Burung 2, 2012 field season, squares D10 and D11. Unpublished report; 2016.) which has currently not yet been accepted for publication. Please remove this from your References and amend this to state in the body of your manuscript: (ie “Bewick et al. [Unpublished]”) as detailed online in our guide for authors

http://journals.plos.org/plosone/s/submission-guidelines#loc-reference-style

**REPLY:** Done.

9. We note that Figures 1 and 3 in your submission contain [map/satellite] images which may be copyrighted. All PLOS content is published under the Creative Commons Attribution License (CC BY 4.0), which means that the manuscript, images, and Supporting Information files will be freely available online, and any third party is permitted to access, download, copy, distribute, and use these materials in any way, even commercially, with proper attribution. For these reasons, we cannot publish previously copyrighted maps or satellite images created using proprietary data, such as Google software (Google Maps, Street View, and Earth). For more information, see our copyright guidelines: http://journals.plos.org/plosone/s/licenses-and-copyright.

We require you to either (1) present written permission from the copyright holder to publish these figures specifically under the CC BY 4.0 license, or (2) remove the figures from your submission:

**REPLY:** Figure 3 was generated entirely by us and does not contain copyrighted data. Figure 1 was produced using GEBCO data, which is free, publicly-available, open source map data, which has been attributed accordingly.

a. You may seek permission from the original copyright holder of Figures 1 and 3 to publish the content specifically under the CC BY 4.0 license.

We recommend that you contact the original copyright holder with the Content Permission Form (http://journals.plos.org/plosone/s/file?id=7c09/content-permission-form.pdf) and the following text:

“I request permission for the open-access journal PLOS ONE to publish XXX under the Creative Commons Attribution License (CCAL) CC BY 4.0 (http://creativecommons.org/licenses/by/4.0/). Please be aware that this license allows unrestricted use and distribution, even commercially, by third parties. Please reply and provide explicit written permission to publish XXX under a CC BY license and complete the attached form.”

Please upload the completed Content Permission Form or other proof of granted permissions as an ""Other"" file with your submission.

**REPLY:** Not applicable, as explained above.

In the figure caption of the copyrighted figure, please include the following text: “Reprinted from [ref] under a CC BY license, with permission from [name of publisher], original copyright [original copyright year].”

**REPLY:** This has been done where applicable.

b. If you are unable to obtain permission from the original copyright holder to publish these figures under the CC BY 4.0 license or if the copyright holder’s requirements are incompatible with the CC BY 4.0 license, please either i) remove the figure or ii) supply a replacement figure that complies with the CC BY 4.0 license. Please check copyright information on all replacement figures and update the figure caption with source information. If applicable, please specify in the figure caption text when a figure is similar but not identical to the original image and is therefore for illustrative purposes only.

The following resources for replacing copyrighted map figures may be helpful:

USGS National Map Viewer (public domain): http://viewer.nationalmap.gov/viewer/

The Gateway to Astronaut Photography of Earth (public domain): http://eol.jsc.nasa.gov/sseop/clickmap/

Maps at the CIA (public domain): https://www.cia.gov/library/publications/the-world-factbook/index.html and https://www.cia.gov/library/publications/cia-maps-publications/index.html

NASA Earth Observatory (public domain): http://earthobservatory.nasa.gov/

Landsat: http://landsat.visibleearth.nasa.gov/

USGS EROS (Earth Resources Observatory and Science (EROS) Center) (public domain): http://eros.usgs.gov/#

Natural Earth (public domain): http://www.naturalearthdata.com/

**REPLY:** Not applicable, as explained above.

10. We note that Figures 2, 4, 5, 6, 7, 8, 9, 10, 11, 12, 13, 14, 16, 17, 19, 20, 21, 22, 24 and 26 in your submission contain copyrighted images. All PLOS content is published under the Creative Commons Attribution License (CC BY 4.0), which means that the manuscript, images, and Supporting Information files will be freely available online, and any third party is permitted to access, download, copy, distribute, and use these materials in any way, even commercially, with proper attribution. For more information, see our copyright guidelines: <http://journals.plos.org/plosone/s/licenses-and-copyright>.

**REPLY:** All of the figures referred to were created using our project data over which we and we alone hold IP and copyright. Elements of *some* images might have been published elsewhere, but just components of them and even in those cases we have modified all elements of these images to produce new figures for the present paper.

We require you to either (1) present written permission from the copyright holder to publish these figures specifically under the CC BY 4.0 license, or (2) remove the figures from your submission:

a. You may seek permission from the original copyright holder of Figures 2, 4, 5, 6, 7, 8, 9, 10, 11, 12, 13, 14, 16, 17, 19, 20, 21, 22, 24 and 26 to publish the content specifically under the CC BY 4.0 license.

We recommend that you contact the original copyright holder with the Content Permission Form (http://journals.plos.org/plosone/s/file?id=7c09/content-permission-form.pdf) and the following text:

“I request permission for the open-access journal PLOS ONE to publish XXX under the Creative Commons Attribution License (CCAL) CC BY 4.0 (http://creativecommons.org/licenses/by/4.0/). Please be aware that this license allows unrestricted use and distribution, even commercially, by third parties. Please reply and provide explicit written permission to publish XXX under a CC BY license and complete the attached form.”

**REPLY:** Not applicable, as explained above.

Please upload the completed Content Permission Form or other proof of granted permissions as an ""Other"" file with your submission.

In the figure caption of the copyrighted figure, please include the following text: “Reprinted from [ref] under a CC BY license, with permission from [name of publisher], original copyright [original copyright year].”

b. If you are unable to obtain permission from the original copyright holder to publish these figures under the CC BY 4.0 license or if the copyright holder’s requirements are incompatible with the CC BY 4.0 license, please either i) remove the figure or ii) supply a replacement figure that complies with the CC BY 4.0 license. Please check copyright information on all replacement figures and update the figure caption with source information. If applicable, please specify in the figure caption text when a figure is similar but not identical to the original image and is therefore for illustrative purposes only.

**REPLY:** Not applicable, as explained above.

11. Please include captions for your Supporting Information files at the end of your manuscript, and update any in-text citations to match accordingly. Please see our Supporting Information guidelines for more information: <http://journals.plos.org/plosone/s/supporting-information>.

**REPLY:** Done.
